# Mitochondria and myocardial ischemia/reperfusion injury: Effects of Chinese herbal medicine and the underlying mechanisms

**DOI:** 10.1016/j.jpha.2024.101051

**Published:** 2024-07-23

**Authors:** Chuxin Zhang, Xing Chang, Dandan Zhao, Yu He, Guangtong Dong, Lin Gao

**Affiliations:** a Beijing University of Chinese Medicine, Beijing, 100029, China; b Guang'anmen Hospital of Chinese Academy of Traditional Chinese Medicine, Beijing, 100053, China

**Keywords:** Myocardial ischemia-reperfusion injury, Mitochondria, Mitochondrial quality control, Oxidative stress, Traditional Chinese medicine

## Abstract

Ischemic heart disease (IHD) is associated with high morbidity and mortality rates. Reperfusion therapy is the best treatment option for this condition. However, reperfusion can aggravate myocardial damage through a phenomenon known as myocardial ischemia/reperfusion (I/R) injury, which has recently gained the attention of researchers. Several studies have shown that Chinese herbal medicines and their natural monomeric components exert therapeutic effects against I/R injury. This review outlines the current knowledge on the pathological mechanisms through which mitochondria participate in I/R injury, focusing on the issues related to energy metabolism, mitochondrial quality control disorders, oxidative stress, and calcium. The mechanisms by which mitochondria mediate cell death have also been discussed. To develop a resource for the prevention and management of clinical myocardial I/R damage, we compiled the most recent research on the effects of Chinese herbal remedies and their monomer components.

## Highlights

• The process of myocardial ischemia-reperfusion causes significant harm.
• The mitochondria operates as both regulators and substrates for damage during I/R.
• ROS, Ca^2+^, energy metabolism, and mitochondrial quality control were included.
• TCMs target mitochondria-mediated alterations and cell death to produce efficacy.

## Graphical abstract

**Figure:**
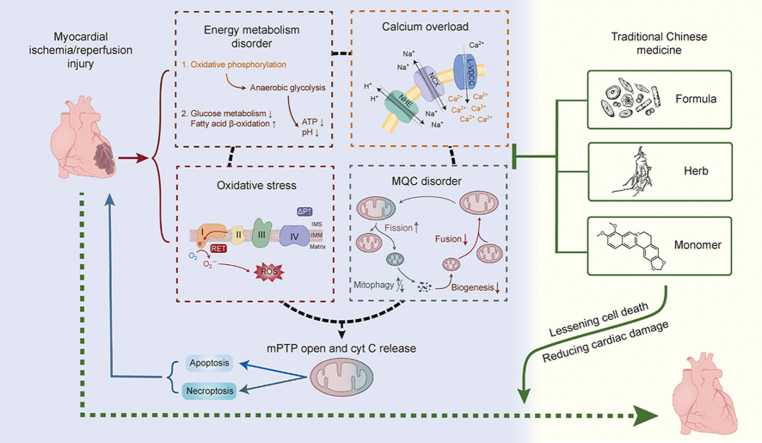

## 1 Introduction

Ischemic heart disease (IHD) is a cardiovascular condition and a leading cause of morbidity and mortality [1]. Reperfusion therapy is the most efficient method for rescuing at-risk heart muscles during myocardial ischemia, particularly in acute myocardial infarction. However, severe damage can occur with the reperfusion of the ischemic myocardial tissue [2,3]. Laboratory experiments have shown that most myocardial cells die within the first few minutes after reperfusion [4,5]. The term myocardial ischemia/reperfusion (I/R) injury is used to describe this condition.

Cardiomyocytes contain abundant mitochondria, which generate energy to sustain continuous muscle contraction and relaxation. They preserve intracellular Ca^2+^ homeostasis and regulate cell death [6]. They play a role in the progression and exacerbation of myocardial I/R injury [7]. During ischemia, a reduction in O_2_ and adenosine triphosphate (ATP) levels disrupts ion homeostasis in myocardial cells, leading to mitochondrial depolarization and cytoplasmic Ca^2+^ accumulation [8]. After reperfusion and mitochondrial membrane potential (MMP; ΔΨm) recovery, excessive Ca^2+^ enters the mitochondria, thereby disrupting mitochondrial quality control (MQC), increasing reactive oxygen species (ROS), activating mitochondrial permeability transition pores (mPTPs), and releasing cytochrome C (cyt C). These phenomena trigger myocardial cell death through apoptosis, necroptosis, and autophagy [[9], [10], [11], [12]]. Hence, identifying therapeutic goals and strategies for treating I/R damage necessitates an understanding of the pathogenic mechanisms of mitochondrial participation in I/R and the cell death pathways they activate. For more than 2000 years, traditional Chinese medicine (TCM), with its own system and abundant resources, has been practiced in China. Over the past two decades, TCM formulas and their monomers have been shown to improve mitochondrial abnormalities and mitigate myocardial injury through several different pathways, such as reducing calcium overload, improving energy metabolism, modulating MQC, and mitigating oxidative stress.

**Fig. 1:**
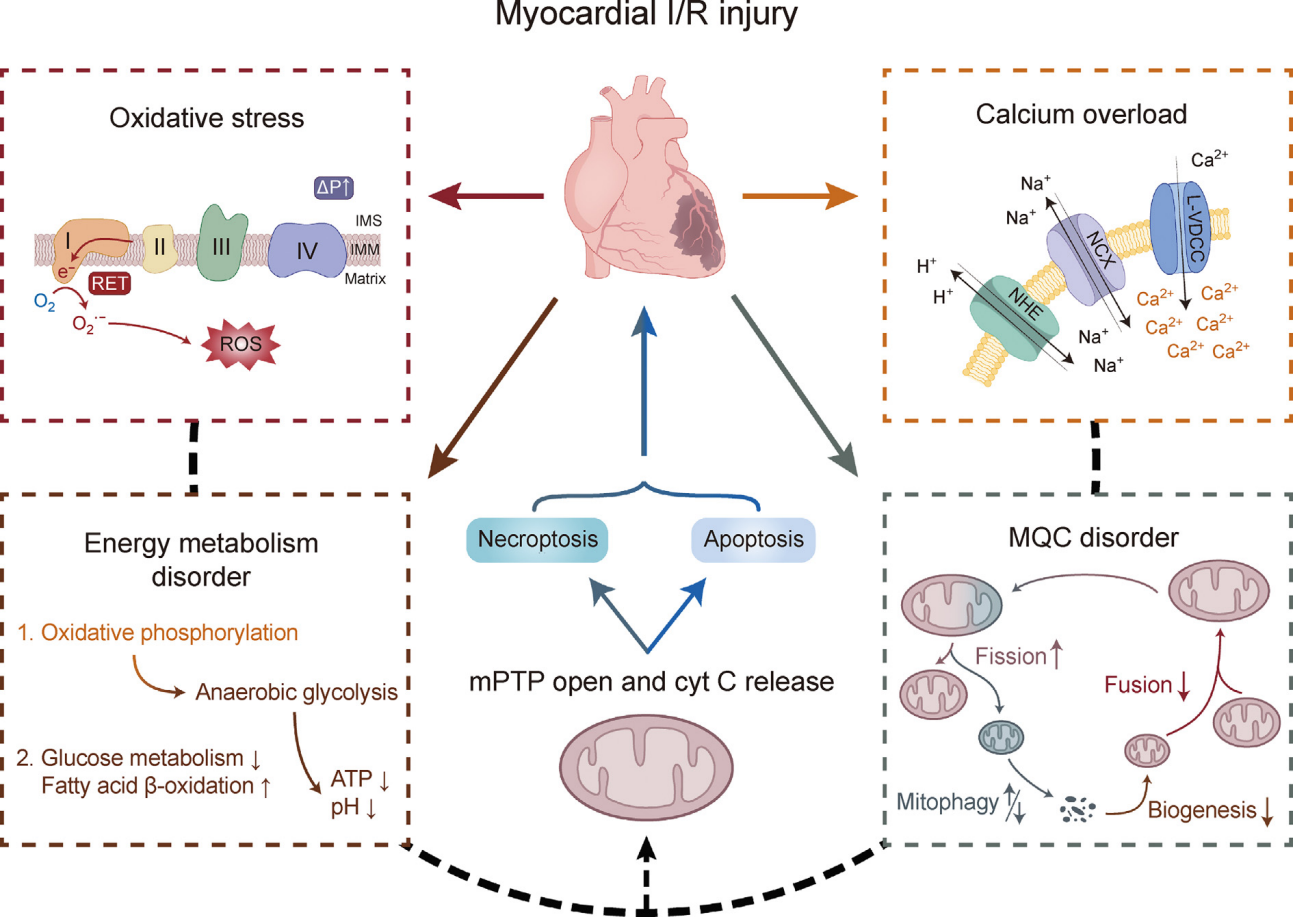
Mechanisms of myocardial ischemia/reperfusion (I/R) injury. ΔP: protonmotive force; IMS: intermembrane space; IMM: inner membrane; RET: reverse electron transport; ROS: reactive oxygen species; NHE: Na^+^/H^+^-exchanger; NCX: Na^+^/Ca^2+^-exchanger; L-VDCC: L-type voltage-dependent Ca^2+^ channel; mPTP: mitochondrial permeability transition pore; cyt C: cytochrome C; ATP: adenosine triphosphate; MQC: mitochondrial quality control.

With emphasis on oxidative stress, calcium overload, energy metabolism, and MQC disorders, this review outlines the current understanding of the pathogenic processes through which mitochondria contribute to myocardial I/R damage. The mechanisms by which mitochondria mediate cell death are shown in Fig. 1. Furthermore, the current effects of Chinese herbal medicines and their monomer components are summarized to create a resource for the prevention and management of clinical cardiac I/R damage.

## 2 Pathological processes in myocardial I/R injury mediated by mitochondria

### 2.1 Oxidative stress

Oxidative stress is the process by which cells are exposed to pathogenic stimuli, resulting in the excessive synthesis of highly active molecules such as ROS and reactive nitrogen species. Adverse reactions that result from an imbalance between the antioxidant and oxidative processes generated by oxidative stress cause tissue damage and ultimately lead to cell death. This is the main cause of cardiac injury during I/R [13]. Excessive ROS formation and a relative deficiency of antioxidant enzymes, including glutathione reductase (GR), reduced glutathione (GSH), and superoxide dismutase (SOD), are major causes of oxidative stress. Mitochondria are both significant generators of ROS and the primary sites of ROS damage [14,15]. Under physiological conditions, low to moderate ROS concentrations are crucial for signaling [16]. Excess ROS production during myocardial I/R is caused by four complex mechanisms: reverse electron transport (RET), ROS-induced ROS release (RIRR), mitochondrial calcium overload, and enzymes of the nicotinamide adenine dinucleotide phosphate (NADPH) oxidase (NOX) family. RET is the main mechanism of the mitochondrial ROS burst during reperfusion [17]. During ischemia, the loss of O_2_ (a terminal electron acceptor) decreases the number of electrons in the coenzyme Q (CoQ) pool. The reverse is true for succinate dehydrogenase (SDH), which uses electrons from the CoQ pool to convert fumarate to succinate, allowing fumarate to function as a terminal electron acceptor [[18], [19], [20]]. After reperfusion, a significant concentration of O_2_ is reintroduced. SDH oxidizes accumulated succinate and returns to normoxic levels [21]. The proton-motive force (ΔP) generates a high proton force, which is activated by complexes III and IV, and the electron transfer chain is restored. Under physiological conditions, ATP synthase uses ΔP to provide energy for the synthesis and release of ATP by shuttling protons across the inner membrane (IMM) into the matrix. However, the early phases of reperfusion are marked by an insufficiency of adenine nucleotides, which limits ATP production and produces a high level of ΔP because adenine nucleotides are degraded during ischemia [22,23]. Elevated ΔP levels hinder the subsequent electron transport from succinate oxidation into the CoQ pool, resulting in the generation of RET. The bulk of proximal ROS is produced at the flavin site of mitochondrial complex I [14]. Additionally, mitochondrial complex III triggers ROS generation partly via redox reactions between O_2_ and ubisemiquinone (UQ^•−^) [24,25]. Finally, the activation of the mPTP is triggered by the superoxide that bursts during reperfusion [26,27], and the breakdown of MMP exacerbates the release and bursting of ROS through the RIRR mechanism [28]. Cell death is triggered by mitochondrial enlargement and rupture, resulting in a chain of subsequent events [29,30].

### 2.2 Calcium overload

Ca^2+^ regulates cellular activity and is mainly distributed in the endoplasmic reticulum and mitochondria. Physiological cellular processes, including cell growth and differentiation, energy metabolism, and excitation-contraction coupling in cardiomyocytes, are controlled by Ca^2+^ in the endoplasmic reticulum and mitochondria, which influences cell activity. It controls physiological activities within cells, including energy consumption, cell division and development, and the excitation-contraction coupling in cardiomyocytes [[31], [32], [33]]. Under physiological conditions, the cell membrane Na^+^/H^+^ exchanger (NHE) maintains equilibrium between Na^+^ and H^+^ both within and outside the cell, whereas the cell membrane Na^+^/Ca^2+^ exchanger (NCX) maintains low levels of internal Ca^2+^ [34,35]. Consequently, the ischemic myocardial metabolism changes from aerobic to anaerobic glycolysis, producing lactic acid and H^+^ [36,37]. As a result of this buildup, the intracellular pH drops, causing the NHE to input Na^+^ and output H^+^. Subsequently, NCX is activated, leading to the exit of excess Na^+^ and entry of extracellular Ca^2+^ into the cells. Through increased NHE activation induced by reperfusion, the cell produces internal H^+^ and rapidly lowers the intracellular pH. Under the action of NCX, cytoplasmic Ca^2+^ levels further increase [38,39]. Overstimulation of Ca^2+^ triggers the activation of Ca^2+^/calmodulin-dependent protein kinase II (CaMKII) self-phosphorylation or allows Ca^2+^ to enter the mitochondria by activating the mitochondrial Ca^2+^ uniporter (MCU). Cell death is induced by mitochondrial dysfunction and excessive mPTP opening [12,40].

Myocardial I/R damage may also be associated with L-type voltage-dependent Ca^2+^ channels (L-VDCCs); however, most studies demonstrating this are inconsistent. In some studies, L-VDCC blockers did not treat I/R damage [41,42]. However, SEA400 (a selective NCX inhibitor) protects the myocardium by reducing calcium overload [41,43]. Furthermore, by breaking down phospholipid components and stimulating external Ca^2+^ flow, ROS buildup can worsen Ca^2+^ overload and damage the cell membrane structure. In addition, ROS can damage the ability of the sarcoplasmic reticulum to absorb Ca^2+^ and increase intracellular Ca^2+^ levels [44].

### 2.3 Mitochondrial energy metabolism disorder

Anaerobic glycolysis replaces oxidative phosphorylation as the primary energy metabolism pathway in mitochondria during ischemia due to hypoxia, which interferes with the ability of myocardial cells to produce ATP. This exacerbates the calcium overload by increasing intracellular lactate levels and lowering pH [36,37,45]. After reperfusion, the breakdown of Caspase acids through β-oxidation accelerates. The recovery of cardiac function is reduced when ATP production increases with an increase in oxygen use [[46], [47], [48]]. During cardiac I/R, the suppression of fatty acid β-oxidation provides protection [49,50]. Additionally, there has been a growing focus on the role of endoplasmic reticulum stress (ERS) in I/R. Unfolded protein response (UPR) has been shown to be initiated by the accumulation of misfolded proteins in the endoplasmic reticulum lumen. UPR mediates mitochondrial energy metabolism disorders during I/R, leading to impaired mitochondrial Ca^2+^ uptake, inhibition of mitochondrial oxidative phosphorylation, and metabolic remodeling [51,52].

### 2.4 Mitochondrial quality control disorder

Mitophagy, mitochondrial biogenesis, fission, and fusion in mitochondria are regulated by MQC, which is an adaptive response. Maintenance of mitochondrial homeostasis is essential for appropriate functioning of the cardiomyocytes [53,54]. Mitochondrial fission and fusion maintain the appropriate balance of mitochondrial components and guarantee the prompt separation of damaged mitochondria. The damaged mitochondria are recycled and broken down via mitophagy and mitochondrial biogenesis. MQC deficiency is mostly associated with anomalies in mitophagy and mitochondrial biogenesis as well as an excessive increase in mitochondrial fission and a reduction in mitochondrial fusion in myocardial I/R damage [[55], [56], [57], [58]].

#### 2.4.1 Mitochondrial fission and fusion

Mitochondria undergo constant reshaping through fission and fusion. To maintain optimal mitochondrial function, mitochondrial fission eliminates damaged or malfunctioning mitochondria from the mitochondrial network [56,59]. By integrating several mitochondrial fragments into filamentous mitochondria via mitochondrial fusion, apoptosis is avoided, and the appropriate proportion of mitochondrial components is maintained. In addition, mitochondrial fusion enables the production of a lengthy and shared electrochemical potential by the mitochondrial network, which guarantees the prompt identification of damaged mitochondrial populations [[60], [61], [62]].

Dynamin-related protein 1 (DRP1) and its receptors control mitochondrial fission, including mitochondrial fission factor (MFF), mitochondrial fission 1 protein (FIS1), mitochondrial dynamic protein 49 kDa (MID49), and MID51 [63,64]. DRP1 mostly dissociates in an inactive state in the cytoplasm under physiological conditions and does not bind to receptors that are linked to the outer mitochondrial membrane (OMM). Therefore, under physiological conditions, mitochondrial fission is inhibited. When DRP1 is under stress, post-transcriptional modifications, such as ubiquitination, acetylation, and phosphorylation, expose its binding site [57,65,66]. Subsequently, it is transported to the mitochondrial surface where it binds to its receptor, thereby triggering mitochondrial fission.

Mitochondrial fusion involves two mechanisms. Mitofusins 1 and 2 (MFN1 and MFN2) are found on the OMM, where they promote homotypic or heterotypic coordination to promote OMM fusion [62]. In contrast, optic atrophy 1 (OPA1) facilitates IMM fusion. Under the influence of yeast mitochondrial escape 1 like 1 ATPase (YME1L1) and overlap with m-AAA 1 zinc metallopeptidase (OMA1), the long isomer of OPA1 (L-OPA1) dissociates into a short isomer (S-OPA1) [67,68]. The equilibrium between the S- and L-OPA1 coordinates mitochondrial fusion in the endometrium [69,70].

Excessive mitochondrial fission occurs during myocardial I/R [71,72]. Other pathogenic alterations induced by excessive mitochondrial fission include a drop in ATP levels, the release of cyt C into the cytoplasm from the mitochondrial intermembrane space (IMS), opening of the mPTP, and dissipation of MMP [[73], [74], [75]]. *In vivo* studies have shown that the level of mitochondrial division is inversely associated with indicators of heart function, such as the left ventricular ejection fraction, and is directly linked to the size of myocardial infarction [76,77]. These findings demonstrate that mitochondrial fission promotes I/R. In contrast, mitochondrial fusion is blocked during I/R. Nevertheless, the role of fusion-related components in this process remains to be elucidated. Previous studies have indicated that hearts with little or no MFN2 display severe mitochondrial DNA (mtDNA) breakage and mitochondrial damage, whereas hearts lacking MFN1 appear to be protected [[78], [79], [80], [81]]. Furthermore, MFN1 knockout cardiomyocytes showed increased activity and decreased mPTP opening in an H_2_O_2_-induced oxidative stress microenvironment [82]. In addition, based on a previous study, hearts lacking MFN1 and MFN2 are less vulnerable to acute I/R damage even under severe mitochondrial dysfunction [83]. Based on the aforementioned data, it appears that although MFN1 and MFN2 promote mitochondrial fusion in a similar manner, their involvement in myocardial I/R damage appears to differ. Additional functions may not have been recorded and thus require further investigation. OPA1 affects mitochondrial function and cardiomyocyte fate. Moreover, OPA1 activation prevents mitochondrial fission and myocardial cell death, whereas OPA1 expression is decreased during myocardial I/R damage [84]. L-OPA1 transforms into S-OPA1 more rapidly when reperfusion triggers self-cleavage and activation of OMA1. This results in an imbalance OPA1 processing, which induces apoptosis, cyt C release, and mitochondrial rupture [85]. Silencing or weakening OPA1 expression leads to mitochondrial damage, promotes superoxide production, and increases infarct size [86,87]. According to these research, myocardial ischemia-reperfusion is protected by mitochondrial fusion, although this protective effect is suppressed.

#### 2.4.2 Mitophagy and mitochondrial biogenesis

Mitophagy, a form of targeted organelle self-degradation, prevents the buildup of faulty mitochondria [88]. To maintain the ongoing development and division of the mitochondrial network and to fulfill the energy demands of cells, amino acids, fatty acids, and other materials are recycled through mitochondrial biogenesis after mitochondrial breakdown [89]. By cycling mitochondrial components, mitophagy and mitochondrial biogenesis cooperate to maintain a healthy mitochondrial network [90].

Recent studies have identified four mitophagy initiation pathways: phosphatase and tensin homolog (PTEN)-induced putative kinase 1 (PINK1)/Parkin, B-cell lymphoma-2 (Bcl-2)/adenovirus E1B 19 kDa protein interacting protein 3 (BNIP3)/nip3-like protein X (NIX), FUN14 domain containing 1 (FUNDC1), and cardiolipin. OMM-expressed BNIP3, FUNDC1, and NIX are receptor-dependent mitophagy initiators [91,92]. PINK1 is mainly located in the cytoplasm, where it forms clusters in mitochondria that have lost their polarization, leading to the phosphorylation of TANK-binding kinase 1 (TBK1) and Parkin [93,94]. Activated Parkin subsequently ubiquitinates several mitochondrial outer-membrane proteins. Further phosphorylation of mitophagy receptors by activated TBK1 occurs in conjunction with microtubule-associated protein 1A/1B light chain 3 (LC3) and ubiquitin interaction areas, including optopurin (OPTN), nuclear dot protein 52 (NDP52), and p62. This process facilitates the crosslinking of ubiquitinated mitochondria with autophagosomes [93,95]. Cardiolipin is located within the IMM. When oxidized, it redistributes and externalizes the surface of damaged mitochondria, thereby initiating mitophagy [96]. LC3 is processed by cysteine proteases into cytoplasmic LC3I, which then binds to phosphatidylethanolamine on the inner and outer membranes of phagophores to form LC3II [97,98]. LC3II interacts with mitophagy receptors to trigger mitophagy. The target mitochondria are engulfed by phagosomes to form autophagosomes. To re-establish equilibrium, autophagosomal proteins, nucleic acids, carbohydrates, and lipids are broken down by lysosomes and retrieved by cells. Studies have shown that the adenosine monophosphate (AMP)-activated protein kinase (AMPK)/Unc-51 like autophagy activating kinase 1 (ULK1) is the main signaling pathway that regulates the progression of mitophagy [92,99,100].

Peroxisome proliferator-activated receptor (PPAR) γ coactivator 1α (PGC-1α) is a key regulating factor in mitochondrial biogenesis. It is induced by an increased energy demand or decreased ATP production, thereby enhancing the expression of many transcription factors [101,102], including nuclear respiratory factor 1 and 2 (NRF1 and NRF2), PPARs, and estrogen-related receptors (ERRs). Transcription of mtDNA is performed by nuclear-encoded mitochondrial transcription factor A (Tfam), which is expressed in response to NRF1/2 [103,104]. PPARs and ERRs produce nuclear proteins that affect glucose uptake, the tricarboxylic acid (TCA) cycle, fatty acid transport and oxidation, and oxidative phosphorylation [[105], [106], [107], [108]].

Overactive autophagy can significantly diminish the mass of mitochondria, resulting in ATP depletion and ultimately cell death. When the number of damaged mitochondria exceeds the ability of mitophagy to eliminate them, or when mitophagy is blocked, mitochondrial quality decreases, leading to cell death. Both mitophagy disorders are induced by pathological conditions. Thus, the effect of mitophagy on cell death remains unclear, as mitophagy plays a role in I/R. For example, Bi et al. [109] demonstrated that mitophagy controlled by PINK1/Parkin is crucial for protecting the heart as it diminishes the beneficial effects of triiodothyronine during I/R and hinders the contractile function of rat hearts. In contrast, research has indicated that following myocardial I/R, excessive autophagy stimulates cardiomyocyte death, which can be prevented by Parkin knockdown [110]. Weakening BNIP3 activity can effectively inhibit mitophagy and cardiomyocyte necrosis [111]. FUNDC1- and cardiolipin-mediated mitophagy differ from the aforementioned pathways and exert cardioprotective effects against I/R [[112], [113], [114], [115]]. These findings could be associated with the duration of I/R, different processing nodes, different activating factors upstream of mitophagy, and the interactions and compensatory effects between different mediating pathways [84,[116], [117], [118]]. Therefore, further studies are warranted.

Mitochondrial network expansion is interrupted during cardiac I/R due to restricted mitochondrial biogenesis, as indicated by decreased expression of PGC-1α and downstream transcription factors [119,120]. During I/R, the reduced levels of PGC-1α and its associated proteins, such as NRF1 and Tfam, are linked to ERS [51]. In addition, mitochondrial biogenesis, mitochondrial dynamics, and mitophagy interact to control each other. For example, PGC-1α participates in regulating mitochondrial fission by affecting DRP1 levels [121]. Its expression can increase and counteract I/R-induced reduction in Mfn1/2 expression in cardiomyocytes [122]. Mitophagy controls mitochondrial biogenesis during I/R, which in turn modulates mitochondrial abundance and homeostasis [123].

## 3 Cell death mechanisms in myocardial I/R injury mediated by mitochondria

The two main mechanisms through which mitochondria cause cardiac cell death during I/R are necroptosis and apoptosis.

### 3.1 Apoptosis

During myocardial I/R, Bid, Bcl-2, and Bax mediate mitochondrial depolarization and cyt C release, which are key steps in activating apoptosis signaling [124]. Bax increases the mitochondrial outer membrane permeability (MOMP), whereas Bcl-2 inhibits this process [125]. Under physiological conditions, heterodimer formation between Bcl-2 and Bax inhibits the pro-apoptotic activity of Bax. However, oxidative stress and calcium overload activate cell death receptors (Fas and tumor necrosis factor (TNF) receptor type 1 (TNF-R1)), upregulate Bax transcription, and increase its cytoplasmic levels. Bax homodimerization promotes the cleavage of Bid into t-Bid. Subsequently, the release of cyt C is affected by the migration and permeation of Bax homodimers into the OMM [126,127]. Cyt C causes conformational alterations and stimulates caspase-9 activation by binding to the C-terminal domain of apoptotic protease activator factor-1 (Apaf-1). Following caspase-9 activation, caspase-3 is cleaved and activated, resulting in nuclear DNA lysis, cell contraction, chromatin condensation, bubbling without rupturing the plasma membrane, apoptotic body production, and cytoskeleton disintegration [128].

### 3.2 Necroptosis

The mPTP-mediated IMM opening is a crucial component of necroptosis-mediated cell death, which is implicated in I/R [129,130]. DRs (TNF-R1, Fas, and TNF related apoptosis inducing ligand receptor (TRAILR)) are activated during the reperfusion phase by oxidative stress and excess calcium levels. Subsequently, downstream molecules are activated, including caspase-8 and caspase-10, Fas-related death domain protein (FADD)/TNF receptor type 1 related death domain protein (TRADD), and receptor-interacting serine/threonine-protein kinases 1 and 3 (RIPK1 and RIPK3) [131]. They interact and produce protein complexes that result in the formation of necrotic complexes including RIPK1, RIPK3, and mixed linear kinase domain like protein (MLKL). Then, the mPTP opens and creates nonspecific pores [[132], [133], [134]], which cause swelling in the mitochondria, malfunctioning of the mitochondrial electron transport chain, and termination of the TCA cycle [132]. Due to ATP depletion, cells experience swelling, plasma membrane disintegration, and organelle rupture, leading to cardiomyocyte death [133].

## 4 The protective effect of Chinese herbal medicine on I/R injury

**Fig. 2:**
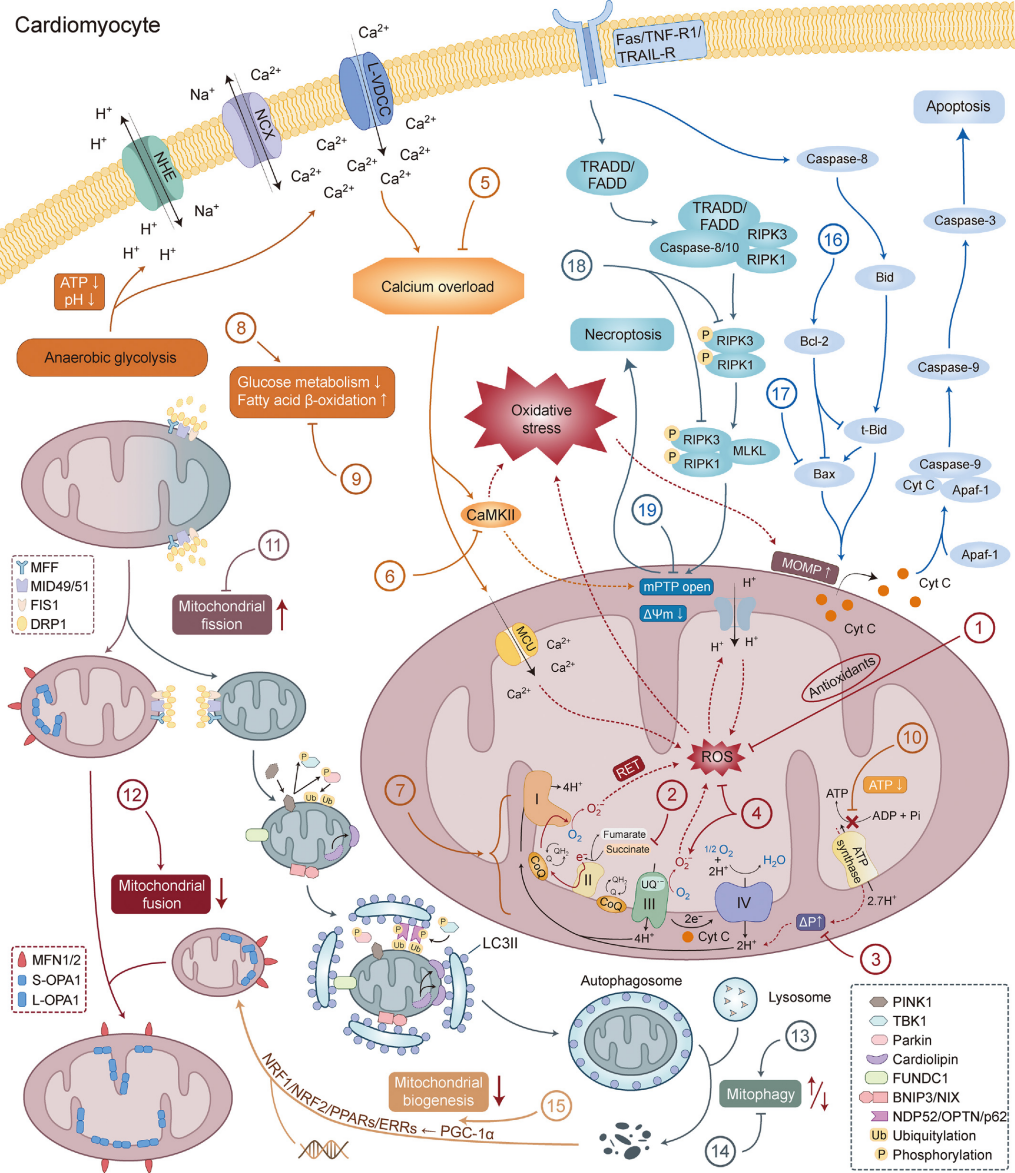
Mitochondria-mediated pathological processes and the targets of Chinese herbal medicine in myocardial ischemia/reperfusion (I/R). 1) Increase antioxidant levels: emodin, oleanolic acid, kaempferol, tanshinone IIA and I, esculetin, geniposide, curcumin, tilianin, tetrahydrocurcumin, aqueous extract of *Cortex Dictamni* and Huangzhi oral liquid. 2) Reduce succinate accumulation: ginsenoside Rb1. 3) Dissipate the proton gradient: ursolic acid. 4) Trigger low level of protective reactive oxygen species (ROS) and eliminate excess ROS: baicalein. 5) Inhibit calcium overload: (−)-epicatechin, (−)-epigallocatechin-3-gallate, eriodictyol, rhynchophylline, hirsutine, naringenin, and Shenmai injection. 6) Inhibit Ca^2+^/calmodulin-dependent protein kinase II (CaMKII) activation: tilianin and hirsutine. 7) Promote mitochondrial respiration: (−)-epicatechin, gypenoside and gypenoside XVII. 8) Promote glucose metabolism: (−)-epicatechin and ginsenoside Rb1. 9) Reduce fatty acid β-oxidation: ginsenoside Rb1 and Zishen Huoxue recipe. 10) Increase adenosine triphosphate (ATP) production: notoginsenoside NR1, tilianin, QiShenYiQi pill, and Zishen Huoxue recipe. 11) Inhibit excessive mitochondrial fission: baicalin, salidroside, crocetin, vitexin, hirsutine, hydroxysafflor yellow A, Shenmai injection, Tongmai formula, and Shen Yuan Dan. 12) Enhance mitochondrial fusion: gypenoside XVII, vitexin, hirsutine, Shenmai injection, Tongmai formula, and Shen Yuan Dan. 13) Promote mitophagy: gerontoxanthone I, macluraxanthone, asiatic acid, *Panax Notoginseng* saponins, hydroxysafflor yellow A, Yiqi Huoxue prescription, Tongxinluo, and Shenmai injection. 14) Reduce mitophagy: Shuangshen Ningxin capsule and Shen Yuan Dan. 15) Promote mitochondrial biogenesis: naringenin and Shen Yuan Dan. 16) Increase the level of B-cell lymphoma-2 (Bcl-2): astragaloside IV, geniposide, eriodictyol, maslinic acid, ginsenosides Rd and Rc, *Panax quinquefolium* saponin, aqueous extract of *Cortex Dictamni*, hydroxytyrosol, asiatic acid, YiQiFuMai powder injection, and Huangzhi oral liquid. 17) Reduce the activation of Bax: parishin J and B and schisandrol A. 18) Dowregulate the expression of receptor-interacting serine/threonine-protein kinase 1 (RIPK1), RIPK3, and mixed linear kinase domain like protein (MLKL): arctiin, tanshinone I, ginsenoside Rg2, and maslinic acid. 19) Inhibit the excessive opening of mitochondrial permeability transition pore (mPTP): tanshinone IIA, picroside II, hydroxysafflor yellow A, rhynchophylline, curculigoside, and sappanone A. NHE: Na^+^/H^+^-exchanger; NCX: Na^+^/Ca^2+^-exchanger; L-VDCC: L-type voltage-dependent Ca^2+^ channel; MFF: mitochondrial fission factor; MID49/51: mitochondrial dynamic protein 49/51 kDa; FIS1: mitochondrial fission 1 protein; DRP1: dynamin-related protein 1; MFN1/2: mitofusin 1/2; OPA1: optic atrophy 1; S-OPA1: short isomer of OPA1; L-OPA1: long isomer of OPA1; NRF1/2: nuclear respiratory factor 1/2; PPARs: peroxisome proliferator-activated receptors; ERRs: estrogen-related receptors; PGC1-α: PPAR γ coactivator 1α; LC3II: microtubule-associated protein 1A/1B light chain 3 II; TNF-R1: tumor necrosis factor (TNF) receptor type 1; TRAIL-R: TNF-related apoptosis-inducing ligand receptor; TRADD: TNF receptor type 1 related death domain protein; FADD: Fas-related death domain protein; MCU: mitochondrial Ca^2+^ uniporter; cyt C: cytochrome C; Apaf-1: apoptotic protease activator factor-1; MOMP: mitochondrial outer membrane permeability; ΔΨm (MMP): mitochondrial membrane potential; RET: reverse electron transport; ADP: adenosine diphosphate; ΔP: protonmotive force; CoQ: coenzyme Q; UQ: ubiquinone; UQ^•−^: ubisemiquinone; PINK1: phosphatase and tensin homolog (PTEN)-induced putative kinase 1; TBK1: TANK-binding kinase 1; FUNDC1: FUN14 domain containing 1; BNIP3: Bcl-2/adenovirus E1B 19 kDa protein interacting protein 3; NIX: Nip3 like protein X; NDP52: nuclear dot protein; OPTN: optopurin.

TCM preparations target mitochondria-mediated alterations and cell death processes to produce therapeutic benefits against I/R damage, as shown in Table 1 [[10], [11], [12],^48^,^55^,^75^,^88^,^127^,^128^,[134], [135], [136], [137], [138], [139], [140], [141], [142], [143], [144], [145], [146], [147], [148], [149], [150], [151], [152], [153], [154], [155], [156], [157], [158], [159], [160], [161], [162], [163], [164], [165], [166], [167], [168], [169], [170], [171], [172], [173], [174], [175], [176], [177], [178], [179], [180], [181], [182], [183], [184], [185], [186], [187], [188], [189]] and Fig. 2.

**Table 1:** Effects of Chinese herbal medicine on myocardial ischemia/reperfusion (I/R) injury.

Category	Name	Classification	Major plant source	Mechanism	Refs.
Formula	Tongmai formula	–	–	**Inhibiting mitochondrial fission:** inhibiting the expression of DRP1. **Promoting mitochondrial fusion:** increasing the expression of MFN2.	[10]
Monomer	Gypenoside XVII	Saponin	*Gynostemma pentaphyllum* (Thunb.) Makino	**Improving energy metabolism:** increasing the basic and maximum oxygen consumption rates and mitochondrial reserve capacity. **Promoting mitochondrial fusion:** increasing the expression of MFN2.	[11,168]
Monomer	Hirsutine	Alkaloid	*Uncaria rhynchophylla* (Miq.) Miq. ex Havil.	**Reducing calcium overload:** reducing cellular Ca^2+^ levels and CaMKII phosphorylation. **Inhibiting mitochondrial fission:** inhibiting the expression of DRP1. **Promoting mitochondrial fusion:** increasing the expression of MFN2.	[12]
Monomer	Tilianin	Flavonoid	*Dracocephalum moldivaca* L.	**Anti-oxidative stress:** increasing mitochondrial SOD levels. **Reducing calcium overload:** Inhibiting the expression of *p*-CaMKII and ox-CaMKII, and activating the JNK/NF-κB signaling pathway. **Improving energy metabolism:** increasing ATP and NAD^+^ content and reducing ADP, AMP content, and AMP/ATP ratio via AMPK/SIRT1/PGC-1α signaling pathway.	[48,[157], [158], [159]]
Monomer	Gerontoxanthone I	Ketone	*Garcinia hanburyi* Hook.f.	**Promoting mitophagy:** through the PINK1/Parkin pathway.	[55]
Monomer	Macluraxanthone	Ketone	*Garcinia hanburyi* Hook.f.	**Promoting mitophagy:** through the PINK1/Parkin pathway.	[55]
Monomer	Crocetin	Carotenoid	*Crocus sativus* L.	**Inhibiting mitochondrial fission:** inhibiting the expression of DRP1 by activating the expression of PGC-1α.	[75]
Formula	Shuangshen Ningxin capsule	–	–	**Inhibiting mitophagy:** inhibiting the expression of PINK1, Parkin, FUNDC1, and BNIP3.	[88]
Monomer	Astragaloside IV	Saponin	*Astragalus membranaceus* (Fisch.) Bunge	**Inhibiting apoptosis:** increasing Bcl-2 levels.	[127]
Monomer	Hydroxytyrosol	Polyphenol	*Ligustrum lucidum* W.T.Aiton	**Inhibiting apoptosis:** increasing Bcl-2/Bax ratio, reducing cyt C release and inhibiting the activation of caspase-9 and caspase-3.	[128]
Monomer	Arctiin	Lignan	*Arctium lappa* L.	**Inhibiting necroptosis:** inhibiting the expression of RIPK1, *p*-RIPK1, RIPK3, *p*-RIPK3, MLKL, and *p*-MLKL.	[134]
Monomer	Asiatic acid	Triterpenoid acid	*Centella asiatica* (L.) Urb.	**Promoting mitophagy:** increasing LC3II level via the AMPK signaling pathway. **Inhibiting apoptosis:** increasing Bcl-2/Bax ratio, reducing cyt C release and inhibiting the activation of caspase-9 and caspase-3 via p38-MAPK and JNK-MAPK signaling pathways.	[135,136]
Monomer	Baicalein	Flavonoid	*Scutellaria baicalensis* Georgi	**Anti-oxidative stress:** Low concentrations (10 μM) triggered low-level mitochondrial ROS with protective effects during ischemia. High concentrations (100 μM) cleared out excess ROS produced during reperfusion.	[[137], [138], [139]]
Monomer	Baicalin	Flavonoid	*Scutellaria baicalensis* Georgi	**Inhibiting mitochondrial fission:** inhibiting the expression of DRP1.	[140]
Monomer	Curculigoside	Saponin	*Curculigo orchioides* Gaertn.	**Inhibiting cell death:** reducing the degree of mPTP opening via Apaf-1, to inhibit the expression of caspase-3 and caspase-9 and the release of cyt C.	[141]
Monomer	Emodin	Anthraquinone	*Rheum palmatum* L.	**Anti-oxidative stress:** enhance the activity and levels of GSH, α-TOC, GR, and SOD	[142,143]
Monomer	Oleanolic acid	Triterpenoid acid	*Eriobotrya japonica* (Thunb.) Lindl. *Cornus officinalis* Siebold et Zucc. *Crataegus pinnatifida* Bunge	**Anti-oxidative stress:** enhance the activity and levels of GSH, α-TOC, GR, and SOD	[143]
Monomer	Esculetin	Coumarin	*Fraxinus rhynchophylla* Hance *Viola yedoensis* Makino	**Anti-oxidative stress:** increasing mitochondrial SOD levels.	[144]
Monomer	Eriodictyol	Flavonoid	*Drynaria fortunei* (Kunze) J.Sm.	**Reducing calcium overload:** reducing cellular Ca^2+^ levels. **Inhibiting apoptosis:** increasing Bcl-2 levels and weakening Bax and caspase-3 expression.	[145]
Monomer	(−)-Epicatechin	Polyphenol	*Acacia catechu* (L.f.) Willd.	**Reducing calcium overload:** reducing mitochondrial Ca^2+^ levels. **Improving energy metabolism:** triggering maximum respiratory rate, activating mitochondrial respiration, improving mitochondrial pyruvate transport to maintain glucose oxidation.	[146]
Monomer	(−)-Epigallocatechin-3-gallate	Polyphenol	*Camellia sinensis* (L.) Kuntze	**Reducing calcium overload:** reducing cellular Ca^2+^ levels.	[147]
Monomer	Ursolic acid	Triterpenoid acid	*Eriobotrya japonica* (Thunb.) Lindl. *Cornus officinalis* Siebold et Zucc. *Crataegus pinnatifida* Bunge	**Anti-oxidative stress:** increasing UCP2 levels and eliminating proton gradients to release respiratory chain coupling via the p38 signaling pathway.	[148]
Monomer	Maslinic acid	Triterpenoid acid	*Eriobotrya japonica* (Thunb.) Lindl. *Crataegus pinnatifida* Bunge	**Inhibiting apoptosis:** increasing Bcl-2 expression and reducing the expression of Bax, cleaved caspase-9 and caspase-3 by promoting autophagy via interacting with LAMP2. **Inhibiting necroptosis:** inhibiting the expression of *p*-RIPK1, *p*-RIPK3 and *p*-MLKL by promoting autophagy via interacting with LAMP2.	[149]
Monomer	Vitexin	Flavonoid	*Crataegus pinnatifida* Bunge	**Inhibiting mitochondrial fission:** inhibiting the expression of DRP1. **Promoting mitochondrial fusion:** increasing the expression of MFN2.	[150]
Monomer	Kaempferol	Flavonoid	*Carthamus tinctorius* L. *Sophora japonica* L. *Uncaria rhynchophylla* (Miq.) Miq. ex Havil.	**Anti-oxidative stress:** increasing SOD levels and the GSH/GSSG ratio	[151]
Monomer	Rhynchophylline	Alkaloid	*Uncaria rhynchophylla* (Miq.) Miq. ex Havil.	**Reducing calcium overload:** reducing cellular Ca^2+^ levels. **Inhibiting cell death:** reducing the degree of mPTP opening to inhibit the expression of caspase-3 and caspase-9 mRNA and the release of cyt C.	[152]
Monomer	Hydroxysafflor Yellow A	Flavonoid	*Carthamus tinctorius* L.	**Promoting mitophagy:** through the HIF-1α/BNIP3 signaling pathway. **Inhibiting mitochondrial fission:** inhibiting the expression of MFF and FIS1. **Inhibiting cell death:** reducing the degree of mPTP opening.	[153,154]
Monomer	Curcumin	Polyphenol/curdione	*Curcuma Longa* L.	**Anti-oxidative stress:** increasing mitochondrial SOD levels	[155]
Monomer	Tetrahydrocurcumin	Polyphenol/curdione	*Curcuma Longa* L.	**Anti-oxidative stress:** inhibiting MDA production and activating antioxidant enzymes SOD and CAT	[156]
Monomer	Tanshinone I	Diterpene quinone	*Salvia miltiorrhiza* Bunge	**Anti-oxidative stress:** increasing mitochondrial antioxidant enzymes NQO-1, HO-1, and SOD levels. **Inhibiting necroptosis:** inhibiting the expression of p-RIP1, *p*-RIP3, and *p*-MLKL.	[160]
Monomer	Tanshinone IIA	Diterpene quinone	*Salvia miltiorrhiza* Bunge.	**Anti-oxidative stress:** increasing mitochondrial SOD levels. **Inhibiting cell death:** reducing mitochondrial permeability transition via the PI3K/Akt signaling pathway	[161,162]
Monomer	Ginsenoside Rb1	Saponin	*Panax ginseng* C. A. Mey.	**Anti-oxidative stress:**inhibiting SDH activity and prevent succinate accumulation. **Improving energy metabolism:** protecting intracellular pyruvate dehydrogenase activity and maintaining glucose oxidation by blocking HIF-1α activation; preventing the entry of free fatty acids into mitochondria by inhibiting CPT1 function, and inhibiting fatty acid oxidation by down-regulating β-oxidation rate-limiting enzymes FACD and KCT.	[163]
Monomer	Ginsenoside Rc	Saponin	*Panax ginseng* C. A. Mey.	**Inhibiting apoptosis:** increasing Bcl-2/Bax ratio, reducing cyt C release and inhibiting the activation of caspase-9 and caspase-3 via SIRT1/FOXO1/Bcl-2 signaling pathway.	[164]
Monomer	Ginsenoside Rd	Saponin	*Panax ginseng* C. A. Mey.	**Inhibiting apoptosis:** increasing Bcl-2/Bax ratio, reducing cyt C release and inhibiting the activation of caspase-9 and caspase-3 via PI3K/Akt signaling pathway.	[165]
Monomer	Ginsenoside Rg2	Saponin	*Panax ginseng C*. A. Mey.	**Inhibiting necroptosis:** inhibiting the expression of RIP1, RIP3, and MLKL and preventing the formation of RIP1/RIP3 complexes.	[166]
Monomer	Gypenoside	Saponin	*Gynostemma pentaphyllum* (Thunb.) Makino	**Improving energy metabolism:** enhancing the activity of the mitochondrial respiratory chain complexes I, II, and IV and citrate synthase in the TCA cycle.	[167]
Monomer	Geniposide	Iridoid glycoside	*Gardenia jasminoides* J.Ellis	**Anti-oxidative stress:** increasing mitochondrial SOD levels. **Inhibiting apoptosis:** increasing Bcl-2 levels and weakening Bax and Caspase-3 expression via PI3K/Akt signaling pathway.	[169]
Monomer	Naringenin	Flavonoid	*Citrus grandis* (L.) Osbeck	**Reducing calcium overload:** activating mitochondrial large-conductance calcium-activated potassium channels (mitoBK). **Promoting mitochondrial biogenesis:** promoting the protein expression of NRF1, TFAM and oxidative phosphorylation complex subunits II/III/IV via the AMPK/SIRT3 signaling pathway	[170,171]
Monomer	Notoginsenoside NR1	Saponin	*Panax notoginseng* (Burkill) F.H. Chen	**Improving energy metabolism:** enhancing the activity of mitochondrial ATP synthase δ subunit.	[172]
Monomer	*Panax Notoginseng* Saponins	Saponin	*Panax notoginseng* (Burkill) F.H. Chen	**Promoting mitophagy:** increasing LC3 expression and LC3II/LC3I ratio via HIF-1α/BNIP3 signaling pathway.	[173]
Monomer	*Panax quinquefolium* saponins	Saponin	*Panax quinquefolius* L.	**Inhibiting apoptosis:** increasing Bcl-2/Bax ratio, reducing cyt C release and inhibiting the activation of caspase-9 and caspase-3.	[174]
Monomer	Salidroside	Alcoholic glycoside	*Rhodiola crenulata* (Hook. f. et Thomson) H. Ohba	**Inhibiting mitochondrial fission:** inhibiting the expression of DRP1 via the AMPK pathway	[175]
Monomer	Parishin B	Phenol	*Gastrodia elata* Blume	**Inhibiting apoptosis:** promoting the phosphorylation of 14-3-3 proteins and increasing their binding to Bax by downregulating the phosphorylation of JNK1, c-Jun and ATF2.	[176]
Monomer	Parishin J	Phenol	*Gastrodia elata* Blume	**Inhibiting apoptosis:** promoting the phosphorylation of 14-3-3 proteins and increasing their binding to Bax by downregulating the phosphorylation of JNK1, c-Jun and ATF2.	[176]
Monomer	Picroside II	Iridoid glycoside	*Picrorhiza scrophulariiflora* Pennell	**Inhibiting cell death:** reducing the degree of mPTP opening, increasing MMP, and inhibiting the release of mitochondrial cyt C	[177]
Monomer	Schisandrol A	Lignan	*Schisandra chinensis* (Turcz.) Baill.	**Inhibiting apoptosis:** increasing Bcl-2/Bax ratio and reducing cleaved caspase-3 levels by upregulating 14-3-3 θ protein.	[178]
Monomer	Sappanone A	Flavonoid	*Caesalpinia sappan* L.	**Inhibiting cell death:** reducing the degree of mPTP opening via PI3K/Akt/Gsk-3β signal pathway, to inhibit the expression of caspase-3 and caspase-9 and the release of cyt C.	[179]
Herb	The aqueous extract of *Cortex Dictamni*	–	*Dictamnus dasycarpus* Turcz.	**Anti-oxidative stress:** increasing mitochondrial SOD levels. **Inhibiting apoptosis:** increasing Bcl-2/Bax ratio, reducing cyt C release and inhibiting the activation of caspase-9 and caspase-3 via PI3K/Akt signaling pathway.	[180]
Formula	Huangzhi Oral Liquid	–	–	**Anti-oxidative stress:** increasing mitochondrial SOD levels. **Inhibiting apoptosis:** increasing Bcl-2 levels and weakening Bax and Caspase-3 expression via p53.	[181]
Formula	QiShenYiQi Pill	–	–	**Improving energy metabolism:** enhancing the expression of ATP synthase subunit, ATP5D, and ATP levels.	[182,183]
Formula	Shenmai Injection	–	–	**Reducing calcium overload:** reducing cellular Ca^2+^ levels. **Promoting mitophagy:** enhancing the expression of PINK1, Parkin, and LC3. **Inhibiting mitochondrial fission:** inhibiting the expression of DRP1 and FIS1. **Promoting mitochondrial fusion:** increasing the expression of MFN1, MFN2, and OPA1.	[184]
Formula	Shen Yuan Dan	–	–	**Inhibiting mitophagy:** through the PINK1/Parkin pathway. **Inhibiting mitochondrial fission:** inhibiting the expression of DRP1 and FIS1. **Promoting mitochondrial fusion:** increasing the expression of MFN1, MFN2, and OPA1. **Promoting mitochondrial biogenesis:** increasing the expression of PGC-1α.	[185]
Formula	Tongxinluo	–	–	**Promoting mitophagy:** through the PINK1/Parkin pathway.	[186]
Formula	YiQiFuMai Powder Injection	–	–	**Inhibiting apoptosis:** increasing Bcl-2 levels and weakening Bax and Caspase-3 expression via AMPK signaling pathway.	[187]
Formula	Yiqi Huoxue prescription	–	–	**Promoting mitophagy:** increasing LC3BII/LC3BI ratio via the PINK1/Parkin and BNIP3/Nix pathways.	[188]
Formula	Zishen Huoxue recipe	–	–	**Improving energy metabolism:** inhibiting fatty acid oxidation and increasing ATP production by activating mTORC1 and inhibiting the expression of 4E-BP.	[189]

−: no data. DRP1: dynamin-related protein 1; MFN2: mitofusin 2; CaMKII: Ca^2+^/calmodulin-dependent protein kinase II; SOD: superoxide dismutase; p-CaMKII: phospho-CaMKII; ox-CaMKII: oxidized-CaMKII; JNK: c-Jun N-terminal kinase; NF-κB: nuclear factor kappaB; ATP: adenosine triphosphate; NAD: nicotinamide adenine dinucleotide; ADP: adenosine diphosphate; AMP: adenosine monophosphate; AMPK: AMP-activated protein kinase; SIRT1: silent mating type information regulation 2 homolog-1; PGC-1α: peroxisome proliferator-activated receptor γ coactivator 1α; PINK1: phosphatase and tensin homolog (PTEN)-induced putative kinase 1; FUNDC1: FUN14 domain containing 1; BNIP3: B-cell lymphoma-2 (Bcl-2)/adenovirus E1B 19kDa protein interacting protein 3; cyt C: cytochrome C; RIPK1: receptor-interacting serine/threonine-protein kinases 1; MLKL: mixed linear kinase domain like protein; LC3II: microtubule-associated protein 1A/1B light chain 3 II; MAPK: p38-mitogen-activated protein kinase; ROS: reactive oxygen species; mPTP: mitochondrial permeability transition pore; Apaf-1: apoptotic protease activator factor-1; GSH: reduced glutathione; α-TOC: alpha-tocopherol; GR: glutathione reductase; UCP2: uncoupling protein 2; LAMP2: lysosomal-associated membrane protein; GSSG: glutathione disulfide; HIF-1α: hypoxia-inducible factor-1; MFF: mitochondrial fission factor; FIS1: mitochondrial fission 1 protein; MDA: malondialdehyde; CAT: catalase; PI3K/Akt: phosphoinositol 3-kinase/protein kinase B; SDH: succinate dehydrogenase; CPT1: carnitine palmityl transferase 1; FACD: fatty acyl CoA dehydrogenase; KCT: 3-ketoacyl CoA thiolase; FOXO1: forkhead box protein O1; TCA: tricarboxylic acid cycle; NRF1: nuclear respiratory factor 1; TFAM: nuclear-encoded mitochondrial transcription factor A; LC3: microtubule-associated protein 1A/1B light chain 3; ATF2: AMP-dependent transcription factor 2; MMP: mitochondrial membrane potential; Gsk-3β: glycogen synthase kinase 3β; ATP5D: ATP synthase subunit delta; OPA1: optic atrophy 1; mTORC1: mammalian target of rapamycin complex 1; 4E-BP: 4E binding protein.

### 4.1 Protection against oxidative stress

Oxidative stress that results in myocardial damage in I/R can be mitigated by increasing antioxidant enzyme activity and preventing ROS production.

Earlier studies have mainly focused on the promoting effects of natural medicinal ingredients on antioxidant levels and activity, which are the basic defense mechanisms against oxidative bursts. For instance, emodin and oleanolic acid increase the activity and concentrations of antioxidant substances such as GSH, α-tocopherol (α-TOC), GR, and SOD [142,143]. Kaempferol confers cardiac protection by increasing SOD levels and the GSH/GSH disulfide (GSSG) ratio and reducing the levels of myocardial injury markers creatine kinase (CK) and lactate dehydrogenase (LDH) [151]. Tanshinone IIA, esculetin, geniposide, curcumin, tilianin, the aqueous extract of *Cortex Dictamni*, and the TCM formula Huangzhi oral liquid increase mitochondrial SOD levels and function as antioxidants during myocardial I/R [48,144,155,162,169,180,181]. The hydrogenation metabolite of curcumin, tetrahydrocurcumin, stimulates the antioxidant enzymes SOD and CAT in H9c2 cardiomyocytes produced by hypoxia/reoxygenation (H/R), inhibits malondialdehyde (MDA) formation, and increases the antioxidant capacity of cardiomyocytes [156]. To combat oxidative stress during I/R, tanshinone I, the main active ingredient in *Salvia miltiorrhiza* Bge, promotes the production of the antioxidant enzymes quinone oxidoreducase-1 (NQO-1), heme oxygenase-1 (HO-1), and SOD [160].

The phases of this process have been the focus of the research on drug effect as RET has been shown to contribute to the development of I/R oxidative stress [17]. Targeting complex I has been demonstrated to reduce RET superoxide production and to have a cardioprotective effect [190,191]. Nevertheless, the long-term inhibition of complex I can disrupt electron flow in the electron transfer chain, resulting in cardiac failure [17]. Therefore, inhibiting SDH is a useful strategy for avoiding succinate oxidation during reperfusion or buildup during ischemia [192,193]. Cardiomyocyte succinate buildup during hypoxia is prevented and SDH activity is inhibited by pretreatment with ginsenoside Rb1 [163]. In *in vitro* cell H/R experiments, ursolic acid increased uncoupling protein 2 (UCP2) levels, eliminated proton gradients to release respiratory chain coupling, and reduced the production of ROS. The p38 signaling pathway may be involved in this process [148].

Administering low concentrations (10 μM) of baicalein shortly before ischemia (10 min) triggered low-level mitochondrial ROS generation through respiratory complex III. These low ROS levels activated survival signaling, thereby maximizing the adaptive response of cardiomyocytes. During reperfusion, MMP activity was restored, mitochondrial integrity and function were maintained, and cardiomyocyte death was minimized. Baicalein protected the heart muscle cells from damage caused by oxygen deprivation and subsequent restoration of blood flow by eliminating surplus ROS generated during recovery when administered at high doses (100 μM) during the initial stages of oxygen deprivation or blood flow recovery, or when administered continuously for 72 h before oxygen deprivation [[137], [138], [139]]. This demonstrates the dual effect of ROS concentration mediated by I/R and emphasizes the differences in drug action concentration, action time node, and action duration on treatment efficacy. Further in-depth research is needed to clarify the administration schedule.

### 4.2 Reducing calcium overload

Excessive Ca^2+^ in the cytoplasm and mitochondria induces excessive ROS production and opening of the mPTP. Targeted inhibition of cytoplasmic and mitochondrial Ca^2+^ production is one strategy used to treat I/R injury. After reperfusion, (−)-epicatechin reduced the mitochondrial calcium overload [146]. Eriodictyol, rhynchophylline, (−)-epigallocatechin-3-gallate, hirsutine, and Shenmai injection (a TCM injection) inhibited intracellular calcium overload in cardiomyocytes [12,145,147,152,184]. Uncertainty persists regarding the precise underlying mechanisms. In the cardiomyocytes of I/R rats, naringenin activated mitochondrial large-conductance calcium-activated potassium channels (mitoBK). This is the mechanism by which naringenin prevents mitochondrial calcium excess [170].

Calcium overload, via the downstream protein CaMKII, triggers cardiac cell death by causing mitochondrial malfunction and ROS production [12,194]. Tilianin interacts with CaMKIIδ, having good binding properties and binding scores. In I/R-damaged cardiomyocytes, it suppresses the production of *p*-CaMKII and ox-CaMKII and stimulates the c-Jun N-terminal kinase (JNK)/nuclear factor kappaB (NF-κB) signaling pathway. Therefore, it is possible to prevent the initiation of apoptotic signaling pathways [158,159]. Hirsutine inhibits mitochondrial dysfunction and myocardial injury by reducing CaMKII phosphorylation during I/R and minimizing oxidative stress levels in tissues [12].

### 4.3 Improving energy metabolism

Improving myocardial metabolism during I/R mainly involves promoting glucose metabolism, inhibiting fatty acid metabolism, enhancing ATPase or mitochondrial respiratory chain complex enzyme activity, increasing oxygen consumption rate, and promoting ATP production. Optimizing energy metabolism is critical for myocardial revascularization and promotes the restoration of cardiac function by delaying ischemic myocardial necrosis.

Pretreatment with the TCM formula QiShenYiQi pill to optimize mitochondrial energy metabolism increases the expression of the mitochondrial ATP synthase subunit, ATP5D, and ATP levels in the cardiomyocytes of I/R rats [182,183]. When (−)-epicatechin was injected intravenously into rats 15 min before to reperfusion, it increased maximal respiratory rate and initiated mitochondrial respiration. It also improved mitochondrial pyruvate transport to maintain glucose oxidation and promote ATP generation [146]. Notoginsenoside NR1 inhibited Rho kinase (ROCK) expression and enhanced the activity of mitochondrial ATP synthase δ subunit in the metabolism of cardiomyocytes [172]. Pretreatment with ginsenoside Rb1 inhibited the production of hypoxia-inducible factor-1α (HIF-1α) and protected cells from intracellular pyruvate dehydrogenase activity, preserving the ability of cells to oxidize glucose. It improved energy metabolism during I/R by preventing free fatty acid entry into mitochondria by inhibiting the function of carnitine palmityl transferase 1 (CPT1) and inhibiting fatty acid oxidation by downregulating the β-oxidation rate-limiting enzymes fatty acyl CoA dehydrogenase (FACD) and 3-ketoacyl CoA thiolase (KCT) [163]. Tilianin pretreatment increased ATP and nicotinamide adenine dinucleotide (NAD)^+^ content in cardiomyocytes of I/R rats, reduced adenosine diphosphate (ADP), AMP content, and AMP/ATP ratio via the AMPK/silent mating type information regulation 2 homolog-1 (SIRT1)/PGC-1α signaling pathway. These effects could be reversed using AMPK and SIRT1 inhibitors [48,157]. The TCM formula Zishen Huoxue recipe inhibited the overexpression of downstream eukaryotic translation initiation factor 4E binding protein (4E-BP) by activating the mammalian target of rapamycin complex 1 (mTORC1), suppressing fatty acid oxidation, increasing ATP production levels, and reducing I/R [189]. Gypenoside increased ATP generation in I/R rat cardiomyocytes by activating citrate synthase in the TCA cycle and mitochondrial respiratory chain complexes I, II, and IV [167]. Pretreatment with gypenoside XVII increased the basic and maximum oxygen consumption rates of cells, as well as their mitochondrial reserve capacity. Moreover, it suppressed the production of proteins linked to the ERS response (glucose regulated protein 78kD (Grp78), protein kinase RNA-like endoplasmic reticulum kinase (PERK), inositol-requiring enzyme-1 (IRE1-α), and activating transcription factor 6 (ATF6)), which may be one mechanism by which it enhances I/R energy metabolism [11,168].

### 4.4 Restoring MQC

Mitochondrial fission, fusion, mitophagy, and biogenesis are potential targets for natural drugs in the treatment of I/R MQC disorders.

Baicalin reduced mitochondrial fission by inhibiting DRP1 synthesis, thereby protecting the myocardium [140]. Salidroside inhibited DRP1 phosphorylation and mitochondrial fission via the AMPK pathway [175]. Gypenoside XVII pretreatment increased MFN2 expression and promoted mitochondrial fusion [11]. By inhibiting excessive mitochondrial fission and promoting fusion, the natural substances vitexin and hirsutine, as well as the TCM formula Tongmai, upregulated the expression of the mitochondrial fusion protein MFN2 and downregulated the expression of the mitochondrial fission regulatory protein DRP1. Thus, they alleviated damage to the mitochondrial ultrastructure caused by H/R [10,12,150]. In H/R-treated H9c2 cardiomyocytes, crocetin decreased DRP1 expression by activating PGC-1α, which could be eliminated by blocking PGC-1α expression [75]. Therefore, PGC-1α participates in both cell mitochondrial fission and mitochondrial biogenesis [195], and is a potential candidate for the treatment of MQC.

Regarding mitophagy and mitochondrial biogenesis, *Panax Notoginseng* saponins significantly increased LC3 expression and LC3II/LC3I ratio in rat myocardium by activating the HIF-1α/BNIP3 signaling pathway [173]. The natural compounds gerontoxanthone I, macluraxanthone, and the TCM formula *Tongxinluo* activated mitophagy via the PINK1/Parkin pathway to degrade dysfunctional mitochondria and ameliorate I/R, both *in vitro* and *in vivo* [55,186]. Asiatic acid increased LC3II levels to promote mitophagy through the AMPK signaling pathway [135]. Yiqi Huoxue enhanced the LC3BII/LC3BI ratio and triggered mitophagy via the PINK1/Parkin and BNIP3/Nix pathways [188]. The ancient Chinese remedy Shuangshen Ningxin capsule, significantly suppressed the activity of PINK1, Parkin, FUNDC1, and BNIP3 in heart muscle cells of miniature pigs with I/R injury. Thus, Shuangshen Ningxin capsules exerted cardioprotective effects by inhibiting mitophagy [88]. Naringenin promoted the protein expression of NRF1, TFAM, and oxidative phosphorylation complex subunits II, III, and IV through the AMPK/SIRT3 signaling pathway, which enhanced mitochondrial biogenesis [171].

Shenmai injection stimulated the expression of PINK1, PARKIN, and LC3, which triggered mitophagy. Moreover, it promoted mitochondrial fusion and suppressed excessive fission. Consequently, *in vitro* mitochondrial homeostasis was preserved in cardiomyocytes subjected to H/R [184]. Hydroxysafflor yellow A inhibited mitochondrial fission by reducing the expression of MFF and FIS1 in the mitochondria and enhanced mitophagy by inducing the HIF-1α/BNIP3 signaling pathway [154]. Pretreatment with Shen Yuan Dan, a TCM formula, hindered mitophagy via the PINK1/Parkin pathway and suppressed the production of DRP1 and FIS1 messenger RNA (mRNA), which in turn prevented mitochondrial fission. It also increased the levels of MFN1/2, OPA1, and PGC-1α mRNA and thereby encouraged mitochondrial fusion and mitochondrial biogenesis [185].

In summary, encouraging mitochondrial fusion, controlling mitophagy, limiting excessive mitochondrial fission, and increasing mitochondrial biogenesis are all aspects of natural medication treatments for MQC. Mitophagy regulates myocardial cell death. The regulation of mitophagy by drugs varies during I/R. The precise regulation of mitophagy is a key issue that requires further research.

### 4.5 Inhibiting cell apoptosis or necroptosis

Bcl-2 is a target of mitochondria-mediated apoptosis. Bcl-2 activation prevents the activation of caspase-9 and caspase-3, which disrupt apoptosis by blocking the Bax-mediated release of mitochondrial cyt C. Astragaloside IV pretreatment significantly increased Bcl-2 levels in cardiomyocytes, thereby inhibiting apoptosis. The selective inhibition of Bcl-2 led to a decrease in its myocardial protective effects [127]. *In vitro* and *in vivo* experiments using geniposide, eriodictyol, and YiQiFuMai powder injection showed that they may decrease the expression of Bax and caspase-3 and increase the levels of Bcl-2 protein. The effect of Huangzhi oral liquid may be mediated by P53. The effect of YiQiFuMai powder injection was mediated by AMPK activation. The phosphoinositol 3-kinase (PI3K)/protein kinase B (Akt) signaling pathway mediated the effects of geniposide [145,169,181,187]. Ginsenosides Rd and Rc, *Panax quinquefolium* saponins, aqueous extracts of *Cortex Dictamni*, hydroxytyrosol, and asiatic acid have all been reported to increase the Bcl-2/Bax ratio, lower cyt C release, and prevent the activation of caspase-9 and caspase-3 after I/R damage [128,136,164,165,174,180]. The PI3K/Akt signaling pathway may be involved in the action of ginsenoside Rd and the aqueous extract of *Cortex Dictamni*. The action of ginsenoside Rc may be mediated by the activation of the SIRT1/forkhead box protein O1 (FOXO1)/Bcl-2 signaling pathway. And the action of asiatic acid has been shown to be mediated by the inhibition of the p38-mitogen-activated protein kinase (MAPK) and JNK-MAPK signaling pathways in *in vitro* and *in vivo* experiments.

Bax is also a candidate inhibitor of apoptosis. 14-3-3 is a cytoplasmic Bax anchoring protein phosphorylation of 14-3-3 leads to the dissociation of Bax from 14-3-3 by triggering the JNK signaling pathway. Bax is transported to the mitochondria to initiate apoptosis [196]. Research has shown that parishin J and B, the active ingredients of the plant *Gastrodia elata*, can reduce the phosphorylation of 14-3-3 proteins and increase their binding to Bax by downregulating the phosphorylation levels of JNK1 and its downstream transcription factors c-Jun and cyclic AMP-dependent transcription factor 2 (ATF2), which exert an inhibitory effect on H/R-induced cardiomyocyte apoptosis [176]. Schisandrol A, the active component of *Schisandra chinensis*, reduces cleaved caspase-3 expression and increases Bcl-2/Bax ratio in addition to upregulating 14-3-3 β protein to mitigate myocardial damage. Furthermore, it easily binds to 14-3-3θ, which is its possible mechanism of action [178].

To reduce necroptosis in I/R cardiomyocytes, current research has mostly concentrated on the targeted effects of medicines on RIPK1, RIPK3, and MLKL, or the regulatory effects of the cascade signals constituting these three [197,198]. Arctiin suppressed the activation of RIPK1, *p*-RIPK1, RIPK3, *p*-RIPK3, MLKL, and *p*-MLKL both *in vivo* and *in vitro*, ultimately inhibiting necroptosis in cardiomyocytes. Furthermore, using the Molecular Operating Environment program, researchers have hypothesized that RIPK1 and MLKL are potential targets of arctiin [134]. After inhibiting *p*-RIP1, *p*-RIP3, and *p*-MLKL expression, tanshinone I demonstrated myocardial protective effects both *in vitro and in vivo* [160]. In H/R cardiomyocytes, ginsenoside Rg2 suppressed RIP1, RIP3, and MLKL phosphorylation and prevented RIP1/RIP3 complex formation [166]. Additionally, by interacting with lysosomal-associated membrane protein 2 (LAMP2), maslinic acid induced apoptosis by downregulating the expression of Bax, cleaved caspase-9, and cleaved caspase-3, increasing the expression of Bcl-2, and reducing the activity of the RIPK1/RIPK3/MLKL axis. Thus, maslinic acid inhibited both I/R cell apoptosis and necroptosis [149].

One strategy to delay cardiomyocyte death by apoptosis or necroptosis is to inhibit excessive opening of the mPTP during I/R. Thus, mPTP is not only a key link in the initiation of necroptosis but also a mediator of oxidative stress bursts and mitochondrial cyt C release in I/R injury [28,29]. A study demonstrated that by triggering the PI3K/Akt signaling pathway, intravenous tanshinone IIA injection prior to reperfusion reduced mitochondrial permeability transition [161]. Picroside II pretreatment reduced the degree of mPTP opening in H/R cardiomyocytes, increased MMP, and inhibited the release of mitochondrial cyt C [177]. Hydroxysafflor yellow A prevented mPTP opening in hypoxic cardiomyocytes, thereby protecting them from damage during reoxygenation [153]. Its cardioprotective effect was similar to that of cyclosporine A (inhibitor of mPTP opening) [199]. Rhynchophylline, the primary active component of *Uncaria rhynchophylla*, inhibited mitochondria-mediated cardiomyocyte apoptosis during oxygen-glucose depletion/reoxygenation by downregulating the expression of caspase-3 and caspase-9 mRNA and cytoplasmic cyt C [152]. Sappanone A and curculigoside both prevented the mPTP from excessive opening, which in turn reduced the release of mitochondrial cyt C and the cleavage of caspase-9 and caspase-3 [141,179]. The protective effects of curculigoside were accompanied by decreased Apaf-1 expression. Sappanone A inhibited mPTP via the PI3K/Akt/glycogen synthase kinase 3β (Gsk-3β) signaling pathway.

## 5 Conclusion and perspectives

Myocardial I/R damage presents a challenge for medical professionals and researchers. Reperfusion, the most effective way to combat IHD, can cause further and more severe injury to the damaged myocardium. Mitochondria operate as indispensable regulators of pathological alterations and targets of damage and functional loss. Ischemia and hypoxia affect mitochondrial energy metabolism, decrease ATP production, and impair mitochondrial function. The resupply of O_2_ after reperfusion increases superoxide production and triggers an influx of Ca^2+^ into cardiomyocytes and mitochondria, further damaging mitochondrial structure and function. Disturbances occur in the MQC mechanism, loss of MMP, stimulation of mPTP opening, and release of cyt C into the cytoplasm. These modifications activate signaling pathways associated with cell fate, resulting in cell death.

However, recent findings on the effect of TCM preparations on calcium overload during I/R are limited to observations, that is, changes in Ca^2+^ levels in cardiomyocytes or mitochondria. Few studies have explored the specific mechanisms involved. Therefore, the potential effects on the expression, activity, and structure of related calcium channel proteins should be further studied. Exploring the potential molecules or signaling pathways involved in the regulatory mechanism is a potential way to increase the understanding of the effect of TCM on calcium overload.

Owing to the complexity of their chemical compositions, the effects of TCM preparations on I/R are often multi-targeted and interwoven. Although this is one of the benefits of using Chinese medicine for therapy, it also poses challenges for pharmacological studies and broad use in myocardial I/R damage. Therefore, few Chinese medicines have advanced to the point of clinical investigation for treating myocardial I/R damage. Further investigations are required to resolve this issue. More specifically, area for further research include the analysis of TCM composition, pharmacological research, toxicological research, screening of single active ingredients, and the development of new drugs. Component analysis techniques, such as high performance liquid chromatography-mass spectrometry (HPLC-MS) and quality evaluation based on quality markers (Q-markers), can be used to identify the active ingredients of TCM preparations. Identification and screening of TCM action targets can be performed using target analysis techniques, such as network pharmacology and molecular docking, or multi-omics high-throughput techniques, such as metabolomics and proteomics. Highly efficient extraction of Chinese medicinal monomers can be achieved using technologies such as macroporous resin adsorption and separation, supercritical fluid extraction, and ultrasonic extraction. Moreover, pharmacokinetic and toxicological studies should be conducted to identify the effective monomer components with fewer adverse effects. In addition, new technologies, such as computer-aided drug design, nano- and microsphere modification, and nanodrug delivery systems, can be used to modify the active monomer structure and optimize the dosage form and administration route of TCM, which is a promising strategy for developing new efficient drugs for myocardial I/R injury. In this review, saponins and flavonoids have been identified as the main TCM monomers known to enhance mitochondrial abnormalities in myocardial I/R injury. While flavonoids are highly effective in scavenging free radicals, saponins primarily improve energy metabolism and suppress apoptosis. These compounds are widely found in plant-derived TCMs. In TCM theory, herbs high in flavonoids are thought to have the properties of “heat-clearing” and “activating blood” whereas herbs high in saponins are thought to have the benefits of “invigorating Qi” and “nourishing Yin”. Therefore, screening for saponins and flavonoids from herbs with the above effects should be considered. This approach will provide a faster and more effective method to screen for effective monomer drugs and develop new drugs against myocardial I/R injury.

## CRediT authorship contribution statement

**Chuxin Zhang:** Conceptualization, Investigation, Writing – original draft. **Xing Chang:** Conceptualization, Writing – original draft, Writing – review & editing. **Dandan Zhao:** Visualization, Writing – original draft. **Yu He:** Visualization, Writing – original draft. **Guangtong Dong:** Writing – original draft, Writing – review & editing. **Lin Gao:** Investigation, Supervision, Writing – original draft.

## Declaration of competing interest

The authors declare that there are no conflicts of interest.
