# Supplementary material for: Mitochondria and myocardial ischemia/reperfusion injury: Effects of Chinese herbal medicine and the underlying mechanisms
Source: J Pharm Anal. 2024 Jul 23;15(2):101051. doi: 10.1016/j.jpha.2024.101051 (PMC11808734; doi:10.1016/j.jpha.2024.101051)
Supplement: Multimedia component 1 [file mmc1.pdf]

20240426163610981274636548206592

WORD COUNT 14519

TIME SUBMITTED 26-APR-2024 04:36PM

PAPER ID 108550193

Mitochondria and myocardial ischemia/reperfusion injury: the effects of Chinese herbal medicine and underlying mechanisms

Abstract

Ischemic heart disease is associated with high morbidity and mortality rates. Reperfusion therapy is the best treatment option for this condition. However, reperfusion can aggravate myocardial damage through a phenomenon known as myocardial ischemia/reperfusion (I/R) injury, which has recently gained the attention of researchers. Several studies have shown that Chinese herbal medicines and their natural monomeric components exert therapeutic effects against I/R injury. This review outlines the current knowledge on the pathological mechanisms through which mitochondria participate in I/R injury, focusing on the issues related to energy metabolism, mitochondrial quality control disorders, oxidative stress, and calcium. The mechanisms by which mitochondria mediate cell death have also been discussed. To develop a resource for the prevention and management of clinical myocardial I/R damage, we compiled the most recent research on the effects of Chinese herbal remedies and their monomer components.

Keywords

Myocardial ischemia-reperfusion injury, Mitochondria, Mitochondrial quality control, Oxidative stress, Traditional Chinese medicine

1. Introduction

Ischemic heart disease (IHD) is a cardiovascular condition and a leading cause of morbidity and mortality [1]. Reperfusion therapy is the most efficient method for rescuing at-risk heart muscles during myocardial ischemia, particularly in acute myocardial infarction. However, severe damage can occur with the reperfusion of the ischemic myocardial tissue [2,3]. Laboratory experiments have shown that most myocardial cells die within the first few minutes after reperfusion [4,5]. The term myocardial ischemia/reperfusion (I/R) injury is used to describe this condition.

Cardiomyocytes contain abundant mitochondria, which generate energy to sustain continuous muscle contraction and relaxation. They preserve intracellular Ca^{2+} homeostasis and regulate cell death. [6]. They play a role in the progression and exacerbation of myocardial I/R injury [7]. During ischemia, a reduction in O_2 and ATP levels disrupts ion homeostasis in myocardial cells, leading to mitochondrial depolarization and cytoplasmic Ca^{2+} accumulation [8]. After reperfusion and mitochondrial membrane potential (MMP, $\Delta\Psi m$) recovery, excessive Ca^{2+} enters the mitochondria, thereby disrupting mitochondrial quality control (MQC), increasing reactive oxygen species (ROS), activating mitochondrial permeability transition pores (mPTP), and releasing cytochrome C (cyt C). These phenomena trigger myocardial

cell death through apoptosis, necroptosis, and autophagy [9–12]. Hence, identifying therapeutic goals and strategies for treating I/R damage necessitates an understanding of the pathogenic mechanisms of mitochondrial participation in I/R and the cell death pathways they activate. For more than 2,000 years, traditional Chinese medicine (TCM), with its own system and abundant resources, has been practiced in China. Over the past two decades, TCM formulas and their monomers have been shown to improve mitochondrial abnormalities and mitigate myocardial injury through several different pathways, such as reducing calcium overload, improving energy metabolism, modulating mitochondrial quality control, and mitigating oxidative stress.

With emphasis on oxidative stress, calcium overload, energy metabolism, and MQC disorders, this review outlines the current understanding of the pathogenic processes through which mitochondria contribute to myocardial I/R damage. The mechanisms by which mitochondria mediate cell death are shown in Fig. 1. Furthermore, the current effects of Chinese herbal medicines and their monomer components are summarized to create a resource for the prevention and management of clinical cardiac I/R damage.

2. Pathological processes in myocardial I/R injury mediated by mitochondria

2.1 Oxidative stress

Oxidative stress is the process by which cells are exposed to pathogenic stimuli, resulting in the excessive synthesis of highly active molecules such as reactive oxygen species (ROS) and reactive nitrogen species (RNS). Adverse reactions that result from an imbalance between the antioxidant and oxidative processes generated by oxidative stress cause tissue damage and ultimately lead to cell death. This is the main cause of cardiac injury during I/R [13]. Excessive ROS formation and a relative deficiency of antioxidant enzymes, including glutathione reductase (GR), reduced glutathione (GSH), and superoxide dismutase (SOD), are major causes of oxidative stress. Mitochondria are both significant generators of ROS and the primary sites of ROS damage [14,15]. Under physiological conditions, low to moderate ROS concentrations are crucial for signaling [16]. Excess ROS production during myocardial I/R is caused by four complex mechanisms: reverse electron transport (RET), ROS-induced ROS release (RIRR), mitochondrial calcium overload, and enzymes of the nicotinamide adenine dinucleotide phosphate (NADPH) oxidase (NOX) family. RET is the main mechanism of the mitochondrial ROS burst during reperfusion [17]. During ischemia, the loss of O_2 (a terminal electron acceptor) decreases the number of electrons in the coenzyme Q pool. The reverse is true for succinate dehydrogenase (SDH), which uses electrons from the coenzyme Q pool to convert fumarate to succinate, allowing fumarate to function as a terminal electron

acceptor [18–20]. After reperfusion, a significant concentration of O_2 is reintroduced. SDH oxidizes accumulated succinate and returns to normoxic levels [21]. The proton-motive force (ΔP) generates a high proton force, which is activated by complexes III and IV, and the electron transfer chain is restored. Under physiological conditions, ATP synthase uses ΔP to provide energy for the synthesis and release of ATP by shuttling protons across the inner membrane (IMM) into the matrix. However, the early phases of reperfusion are marked by an insufficiency of adenine nucleotides, which limits ATP production and produces a high level of ΔP because adenine nucleotides are degraded during ischemia [22,23]. Elevated ΔP levels hinder the subsequent electron transport from succinate oxidation into the coenzyme Q pool, resulting in the generation of RET. The bulk of proximal ROS is produced at the flavin site of mitochondrial complex I [14]. Additionally, mitochondrial complex III triggers ROS generation partly via redox reactions between O_2 and ubiquinone ($UQ^{\bullet -}$) [24,25]. Finally, the activation of the mPTP is triggered by the superoxide that bursts during reperfusion [26,27], and the breakdown of MMP exacerbates the release and bursting of ROS through the RIRR mechanism [28]. Cell death is triggered by mitochondrial enlargement and rupture, resulting in a chain of subsequent events [29,30].

2.2 Calcium overload

Ca^{2+} regulates cellular activity and is mainly distributed in the endoplasmic reticulum and mitochondria. Physiological cellular processes, including cell growth and differentiation, energy metabolism, and excitation-contraction coupling in cardiomyocytes, are controlled by Ca^{2+} in the endoplasmic reticulum and mitochondria, which influences cell activity. It controls physiological activities within cells, including energy consumption, cell division and development, and the excitation-contraction coupling in cardiomyocytes [31–33]. Under physiological conditions, the cell membrane Na^+/H^+ exchanger (NHE) maintains equilibrium between Na^+ and H^+ both within and outside the cell, whereas the cell membrane Na^+/Ca^{2+} exchanger (NCX) maintains low levels of internal Ca^{2+} [34,35]. Consequently, the ischemic myocardial metabolism changes from aerobic to anaerobic glycolysis, producing lactic acid and H^+ [36,37]. As a result of this buildup, the intracellular pH drops, causing the NHE to input Na^+ and output H^+. Subsequently, NCX is activated, leading to the exit of excess Na^+ and entry of extracellular Ca^{2+} into the cells. Through increased NHE activation induced by reperfusion, the cell produces internal H^+ and rapidly lowers the intracellular pH. Cytoplasmic Ca^{2+} levels further increase as a result of NCX [38,39]. Overstimulation of Ca^{2+} triggers the activation of Ca^{2+}/calmodulin-dependent protein kinase II (CaMKII) self-phosphorylation or allows Ca^{2+} to enter the mitochondria by activating the mitochondrial Ca^{2+} uniporter

(MCU). Cell death is induced by mitochondrial dysfunction and excessive mPTP opening [12,40].

Myocardial I/R damage may also be associated with L-type voltage-dependent Ca^{2+} channels (L-VDCCs); however, most studies demonstrating this are inconsistent. In some studies, L-VDCC blockers did not treat I/R damage [41,42]. However, SEA400 (a selective NCX inhibitor) protects the myocardium by reducing calcium overload [41,43]. Furthermore, by breaking down phospholipid components and stimulating external Ca^{2+} flow, ROS buildup can worsen Ca^{2+} overload and damage the cell membrane structure. In addition, ROS can damage the ability of the sarcoplasmic reticulum to absorb Ca^{2+} and increase intracellular Ca^{2+} levels [44].

2.3 Mitochondrial energy metabolism disorder

Anaerobic glycolysis replaces oxidative phosphorylation as the primary energy metabolism pathway in mitochondria during ischemia due to hypoxia, which interferes with the ability of myocardial cells to produce ATP. This exacerbates the calcium overload by increasing intracellular lactate levels and lowering pH [36,37,45]. After reperfusion, the breakdown of fatty acids through β-oxidation accelerates. The recovery of cardiac function is reduced when ATP production increases with an increase in oxygen use [46–48]. During cardiac I/R, the suppression of fatty acid β-oxidation provides protection [49,50]. Additionally, there has been a growing focus on the role of endoplasmic reticulum stress (ERS) in I/R. Unfolded protein response (UPR) has been shown to be initiated by the accumulation of misfolded proteins in the endoplasmic reticulum lumen. UPR mediates mitochondrial energy metabolism disorders during I/R, leading to impaired mitochondrial Ca^{2+} uptake, inhibition of mitochondrial oxidative phosphorylation, and metabolic remodeling [51,52].

2.4 Mitochondrial quality control disorder

Mitophagy, mitochondrial biogenesis, fission, and fusion in mitochondria are regulated by MQC, which is an adaptive response. Maintenance of mitochondrial homeostasis is essential for appropriate functioning of the cardiomyocytes [53,54]. Mitochondrial fission and fusion maintain the appropriate balance of mitochondrial components and guarantee the prompt separation of damaged mitochondria. The damaged mitochondria are recycled and broken down via mitophagy and mitochondrial biogenesis. MQC deficiency is mostly associated with anomalies in mitophagy and mitochondrial biogenesis as well as an excessive increase in mitochondrial fission and a reduction in mitochondrial fusion in myocardial I/R damage [55–58].

2.4.1 *Mitochondrial fission and fusion*

Mitochondria undergo constant reshaping through fission and fusion. To maintain optimal mitochondrial function, mitochondrial fission eliminates damaged or malfunctioning mitochondria from the mitochondrial network [56,59]. By integrating several mitochondrial fragments into filamentous mitochondria via mitochondrial fusion, apoptosis is avoided, and the appropriate proportion of mitochondrial components is maintained. In addition, mitochondrial fusion enables the production of a lengthy and shared electrochemical potential by the mitochondrial network, which guarantees the prompt identification of damaged mitochondrial populations [60–62].

Dynamin-related protein 1 (DRP1) and its receptors control mitochondrial fission, including mitochondrial fission factor (MFF), mitochondrial fission 1 protein (FIS1), mitochondrial dynamic protein 49 kDa (MID49), and mitochondrial dynamic protein 51 kDa (MID51) [63,64]. DRP1 mostly dissociates in an inactive state in the cytoplasm under physiological conditions and does not bind to receptors that are linked to the outer mitochondrial membrane (OMM). Therefore, under physiological conditions, mitochondrial fission is inhibited. When DRP1 is under stress, post-transcriptional modifications, such as ubiquitination, acetylation, and phosphorylation expose its binding site [57,65,66]. Subsequently, it is transported to the mitochondrial surface where it binds to its receptor, thereby triggering mitochondrial fission.

Mitochondrial fusion involves two mechanisms. Mitofusins 1 and 2 (MFN1 and MFN2) are found on the OMM, where they promote homotypic or heterotypic coordination to promote OMM fusion [62]. In contrast, optic atrophy 1 (OPA1) facilitates IMM fusion. Under the influence of yeast mitochondrial escape 1 like 1 ATPase (YME1L1) and overlap with m-AAA 1 zinc metallopeptidase (OMA1), the long isomer of OPA1 (L-OPA1) dissociates into a short isomer (S-OPA1) [67,68]. The equilibrium between the S- and L-OPA1 coordinates mitochondrial fusion in the endometrium [69,70].

Excessive mitochondrial fission occurs during myocardial I/R [71,72]. Other pathogenic alterations induced by excessive mitochondrial fission include a drop in ATP levels, the release of cyt C into the cytoplasm from the mitochondrial intermembrane space (IMS), opening of the mPTP, and dissipation of MMP [73–75]. *In vivo* studies have shown that the level of mitochondrial division is inversely associated with indicators of heart function, such as the left ventricular ejection fraction, and is directly linked to the size of myocardial infarction [76,77]. These findings demonstrate that mitochondrial fission promotes I/R. In contrast, mitochondrial fusion is blocked during I/R. Nevertheless, the role of fusion-related components in this process remains to be elucidated. Previous studies have indicated that hearts with little or no MFN2 display severe mtDNA breakage and mitochondrial

damage, whereas hearts lacking MFN1 appear to be protected [78–81]. Furthermore, MFN1 knockout cardiomyocytes showed increased activity and decreased mPTP opening in an H_2O_2-induced oxidative stress microenvironment [82]. In addition, based on a previous study, hearts lacking MFN1 and MFN2 are less vulnerable to acute I/R damage even under severe mitochondrial dysfunction [83]. Based on the aforementioned data, it appears that although MFN1 and MFN2 promote mitochondrial fusion in a similar manner, their involvement in myocardial I/R damage appears to differ. Additional functions may not have been recorded and thus require further investigation. OPA1 affects mitochondrial function and cardiomyocyte fate. Moreover, OPA1 activation prevents mitochondrial fission and myocardial cell death, whereas OPA1 expression is decreased during myocardial I/R damage [84]. L-OPA1 transforms into S-OPA1 more rapidly when reperfusion triggers self-cleavage and activation of OMA1. This results in an imbalance OPA1 processing, which induces apoptosis, cyt C release, and mitochondrial rupture [85]. Silencing or weakening OPA1 expression leads to mitochondrial damage, promotes superoxide production, and increases infarct size [86,87]. According to these studies, myocardial ischemia-reperfusion is protected by mitochondrial fusion; however, this protective effect is suppressed.

2.4.2 Mitophagy and mitochondrial biogenesis

Mitophagy, a form of targeted organelle self-degradation, prevents the build-up of faulty mitochondria [88]. To maintain the ongoing development and division of the mitochondrial network and to fulfill the energy demands of cells, amino acids, fatty acids, and other materials are recycled through mitochondrial biogenesis after mitochondrial breakdown [89]. By cycling mitochondrial components, mitophagy and mitochondrial biogenesis cooperate to maintain a healthy mitochondrial network [90].

Recent studies have identified four mitophagy initiation pathways: PTEN-induced putative kinase 1 (PINK1)/Parkin, BCL2/adenovirus E1B 19kDa protein interacting protein 3 (BNIP3)/NIP3 like protein X (NIX), FUN14 domain containing 1 (FUNDC1), and cardiolipin. OMM-expressed BNIP3, FUNDC1, and NIX are receptor-dependent mitophagy initiators [91,92]. PINK1 is mainly located in the cytoplasm, where it forms clusters in mitochondria that have lost their polarization, leading to the phosphorylation of TBK1 and Parkin [93,94]. Activated Parkin subsequently ubiquitinates several mitochondrial outer-membrane proteins. Further phosphorylation of mitophagy receptors by activated TBK1 occurs in conjunction with microtubule-associated protein 1A/1B light chain 3 (LC3) and ubiquitin interaction areas, including optopurin (OPTN), nuclear dot protein 52 (NDP52), and p62. This process facilitates the crosslinking of ubiquitinated mitochondria with autophagosomes [93,95]. Cardiolipin is located within the IMM. When oxidized, it

redistributes and externalizes the surface of damaged mitochondria, thereby initiating mitophagy [96]. LC3 is processed by cysteine proteases into cytoplasmic LC3I, which then binds to phosphatidylethanolamine on the inner and outer membranes of phagophores to form LC3II [97,98]. LC3II interacts with mitophagy receptors to trigger mitophagy. The target mitochondria are engulfed by phagosomes to form autophagosomes. To re-establish equilibrium, autophagosomal proteins, nucleic acids, carbohydrates, and lipids are broken down by lysosomes and retrieved by cells. Studies have shown that the AMP-activated protein kinase (AMPK)/Unc-51 like autophagy activating kinase 1 (ULK1) is the main signaling pathway that regulates the progression of mitophagy [92,99,100].

Peroxisome proliferator-activated receptor γ coactivator 1α (PGC-1α) is a key regulating factor in mitochondrial biogenesis. It is induced by an increased energy demand or decreased ATP production, thereby enhancing the expression of many transcription factors [101,102], including nuclear respiratory factor 1 and 2 (NRF1 and NRF2), peroxisome proliferator-activated receptors (PPARs) and estrogen-related receptors (ERRs). Transcription of mtDNA is performed by nuclear-encoded mitochondrial transcription factor A (Tfam), which is expressed in response to NRF1/2 [103,104]. PPARs and ERRs produce nuclear proteins that affect glucose uptake, the tricarboxylic acid cycle, fatty acid transport and oxidation, and oxidative phosphorylation [105–108].

Overactive autophagy can significantly diminish the mass of mitochondria, resulting in ATP depletion and ultimately, cell death. When the number of damaged mitochondria exceeds the ability of mitophagy to eliminate them, or when mitophagy is blocked, mitochondrial quality decreases, leading to cell death. Both mitophagy disorders are induced by pathological conditions. Thus, the effect of mitophagy on cell death remains unclear, as mitophagy plays a role in I/R. For example, Bi et al. [109] demonstrated that mitophagy controlled by PINK1/Parkin is crucial for protecting the heart as it diminishes the beneficial effects of triiodothyronine during I/R and hinders the contractile function of rat hearts. In contrast, research has indicated that following myocardial I/R, excessive autophagy stimulates cardiomyocyte death, which can be prevented by Parkin knockdown [110]. Weakening BNIP3 activity can effectively inhibit mitophagy and cardiomyocyte necrosis [111]. FUNDC1- and cardiolipin-mediated mitophagy differ from the aforementioned pathways and exert cardioprotective effects against I/R [112–115]. These findings could be associated with the duration of I/R, different processing nodes, different activating factors upstream of mitophagy, and the interactions and compensatory effects between different mediating pathways [84,116–118]. Therefore, further studies are warranted.

Mitochondrial network expansion is interrupted during cardiac I/R due to restricted mitochondrial biogenesis, as indicated by decreased expression of PGC-1α

and downstream transcription factors [119,120]. During I/R, the reduced levels of PGC-1α and its associated proteins, such as NRF1 and Tfam, are linked to endoplasmic reticulum stress [51]. In addition, mitochondrial biogenesis, mitochondrial dynamics, and mitophagy interact to control each other. For example, PGC-1α participates in regulating mitochondrial fission by affecting DRP1 levels [121]. Its expression can increase and counteract I/R-induced reduction in Mfn1/2 expression in cardiomyocytes [122]. Mitophagy controls mitochondrial biogenesis during I/R, which in turn modulates mitochondrial abundance and homeostasis [123].

3 Cell death mechanisms in myocardial I/R injury mediated by mitochondria

The two main mechanisms through which mitochondria cause cardiac cell death during I/R are necroptosis and apoptosis.

3.1 Apoptosis

During myocardial I/R, Bid, Bcl-2, and Bax mediate mitochondrial depolarization and cyt C release, which are key steps in activating apoptosis signaling [124]. Bax increases the mitochondrial outer membrane permeability (MOMP), whereas Bcl-2 inhibits this process [125]. Under physiological conditions, heterodimer formation between Bcl-2 and Bax inhibits the pro-apoptotic activity of Bax. However, oxidative stress and calcium overload activate cell death receptors (Fas and TNF-R1), upregulate Bax transcription, and increase its cytoplasmic levels. Bax homodimerization promotes the cleavage of Bid into t-Bid. Subsequently, the release of cyt C is affected by the migration and permeation of Bax homodimers into the OMM [126,127]. Cyt C causes conformational alterations and stimulates caspase-9 activation by binding to the C-terminal domain of apoptotic protease activator factor-1 (Apaf-1). Following caspase-9 activation, caspase-3 is cleaved and activated, resulting in nuclear DNA lysis, cell contraction, chromatin condensation, bubbling without rupturing the plasma membrane, apoptotic body production, and cytoskeleton disintegration [128].

3.2 Necroptosis

The mPTP-mediated IMM opening is a crucial component of necroptosis-mediated cell death, which is implicated in I/R [129,130]. DRs (TNF-R1, FAS, and TRAILR) are activated during the reperfusion phase by oxidative stress and excess calcium levels. Subsequently, downstream molecules are activated, including caspase-8 and -10, Fas-related death domain protein (FADD)/TNF receptor type 1 related death domain protein (TRADD), and receptor-interacting

serine/threonine-protein kinases 1 and 3 (RIPK1 and RIPK3) [131]. They interact and produce protein complexes that result in the formation of necrotic complexes including RIPK1, RIPK3, and mixed linear kinase domain like protein (MLKL). Then, the mPTP opens and creates non-specific pores [132–134], which cause swelling in the mitochondria, malfunctioning of the mitochondrial electron transport chain, and termination of the tricarboxylic acid cycle [132]. Due to ATP depletion, cells experience swelling, plasma membrane disintegration, and organelle rupture, leading to cardiomyocyte death [133].

4 The protective effect of Chinese herbal medicine on I/R injury

4.1 Protection against oxidative stress

Oxidative stress that results in myocardial damage in I/R can be mitigated by increasing antioxidant enzyme activity and preventing ROS production. Earlier studies have mainly focused on the promoting effects of natural medicinal ingredients on antioxidant levels and activity, which are the basic defense mechanisms against oxidative bursts. For instance, emodin and oleanolic acid, increase the activity and concentrations of antioxidant substances such GSH, α-tocopherol (α-TOC), GR, and SOD [134,135]. Kaempferol confers cardiac protection by increasing SOD levels and the GSH/glutathione disulfide (GSSG) ratio and reducing the levels of myocardial injury markers CK and LDH [136]. Tanshinone IIA, esculetin, geniposide, curcumin, tilianin, the aqueous extract of *Cortex Dictamni* and the TCM formula *Huangzhi oral liquid* increase mitochondrial SOD levels and function as antioxidants during myocardial I/R [48,137–142]. The hydrogenation metabolite of curcumin, tetrahydrocurcumin, stimulates the antioxidant enzymes SOD and CAT in H9c2 cardiomyocytes produced by hypoxia/reoxygenation (H/R), inhibits MDA formation, and increases the antioxidant capacity of cardiomyocytes [143]. To combat oxidative stress during I/R, tanshinone I, the main active ingredient in *Salvia miltiorrhiza Bge*, promotes the production of the antioxidant enzymes quinone oxidoreducase-1 (NQO-1), heme oxygenase-1 (HO-1), and SOD [144].

The phases of this process have been the focus of the research on drug effect as RET has been shown to contribute to the development of I/R oxidative stress [17]. Targeting complex I has been demonstrated to reduce RET superoxide production and to have a cardioprotective effect [145,146]. Nevertheless, the long-term inhibition of complex I can disrupt electron flow in the electron transfer chain, resulting in cardiac failure [17]. Therefore, inhibiting SDH is a useful strategy for avoiding succinate oxidation during reperfusion or buildup during ischemia [147,148]. Cardiomyocyte succinate buildup during hypoxia is prevented and SDH activity is inhibited by pretreatment with ginsenoside Rb1 [149]. In *in vitro* cell H/R experiments, ursolic

acid increased uncoupling protein 2 (UCP2) levels, eliminated proton gradients to release respiratory chain coupling, and reduced the production of reactive oxygen species. The p38 signaling pathway may be involved in this process [150].

Administering low concentrations (10 μM) of baicalin shortly before ischemia (10 min) triggered low-level mitochondrial ROS generation through respiratory complex III. These low ROS levels activated survival signaling, thereby maximizing the adaptive response of cardiomyocytes. During reperfusion, MMP activity was restored, mitochondrial integrity and function were maintained, and cardiomyocyte death was minimized. Baicalin protected the heart muscle cells from damage caused by oxygen deprivation and subsequent restoration of blood flow by eliminating surplus reactive oxygen species generated during recovery when administered at high doses (100 μM) during the initial stages of oxygen deprivation or blood flow recovery, or when administered continuously for 72 h before oxygen deprivation [151–153]. This demonstrates the dual effect of ROS concentration mediated by I/R, and emphasizes the differences in drug action concentration, action time node, and action duration on treatment efficacy. Further in-depth research is needed to clarify the administration schedule.

4.2 Reducing calcium overload

Excessive Ca^{2+} in the cytoplasm and mitochondria induces excessive ROS production and opening of the mPTP. Targeted inhibition of cytoplasmic and mitochondrial Ca^{2+} production is one strategy used to treat I/R injury. After reperfusion, (-)-epicatechin reduced the mitochondrial calcium overload [154]. Eriodictyol, rhynchophylline, (-)-epigallocatechin-3-gallate, hirsutine, and *Shenmai* injection (TCM) inhibited intracellular calcium overload in cardiomyocytes [12,155–158]. Uncertainty persists regarding the precise underlying mechanisms. In the cardiomyocytes of I/R rats, naringenin activated mitochondrial large-conductance calcium-activated potassium channels (mitoBK). This is the mechanism by which naringenin prevents mitochondrial calcium excess [159].

Calcium overload, via the downstream protein CaMKII, triggers cardiac cell death by causing mitochondrial malfunction and ROS production [12,160]. Tilianin interacts with CaMKIIδ having good binding properties and binding scores. In I/R-damaged cardiomyocytes, it suppresses the production of p-CaMKII and ox-CaMKII and stimulates the JNK/NF-κb signaling pathway. Therefore, it is possible to prevent the initiation of apoptotic signaling pathways [161,162]. Hirsutine inhibits mitochondrial dysfunction and myocardial injury by reducing CaMKII phosphorylation during I/R and minimizing oxidative stress levels in tissues [12].

4.3 Improving energy metabolism

Improving myocardial metabolism during I/R mainly involves promoting glucose metabolism, inhibiting fatty acid metabolism, enhancing ATPase or mitochondrial respiratory chain complex enzyme activity, increasing oxygen consumption rate, and promoting ATP production. Optimizing energy metabolism is critical for myocardial revascularization and promotes the restoration of cardiac function by delaying ischemic myocardial necrosis.

Pretreatment with the TCM formula QiShenYiQi pill to optimize mitochondrial energy metabolism increases the expression of the mitochondrial ATP synthase subunit, ATP5D, and ATP levels in the cardiomyocytes of I/R rats [163,164]. When (-)-epicatechin was injected intravenously into rats 15 min before to reperfusion, it increased maximal respiratory rate and initiated mitochondrial respiration. It also improved mitochondrial pyruvate transport to maintain glucose oxidation and promote ATP generation [154]. Notoginsenoside NR1 inhibited Rho kinase (ROCK) expression and enhanced the activity of mitochondrial ATP synthase δ subunit in the metabolism of cardiomyocytes [165]. Pretreatment with ginsenoside Rb1 inhibited the production of hypoxia-inducible factor-1 (HIF-1α) and protected cells from intracellular pyruvate dehydrogenase activity, preserving the ability of cells to oxidize glucose. It improved energy metabolism during I/R by preventing free fatty acid entry into mitochondria by inhibiting the function of carnitine palmityl transferase I (CPT1) and inhibiting fatty acid oxidation by downregulating the β-oxidation rate-limiting enzymes fatty acyl CoA dehydrogenase (FACD) and 3-ketoacyl CoA thiolase (KCT) [149]. Tilianin pretreatment increased ATP and NAD^+ content in cardiomyocytes of I/R rats, reduced ADP, AMP content, and AMP/ATP ratio via the AMPK/Silent mating type information regulation 2 homolog-1 (SIRT1)/PGC-1α signaling pathway. These effects could be reversed using AMPK and SIRT1 inhibitors [48,166]. The TCM formula *Zishen Huoxue recipe* inhibited the overexpression of downstream eukaryotic translation initiation factor 4E binding protein (4E-BP) by activating the mammalian target of rapamycin complex 1 (mTORC1), suppressing fatty acid oxidation, increasing ATP production levels, and reducing I/R [167]. Gypenoside increased ATP generation in I/R rat cardiomyocytes by activating citrate synthase in the tricarboxylic acid cycle and mitochondrial respiratory chain complexes I, II, and IV [168]. Pretreatment with Gyp XVII increased the basic and maximum oxygen consumption rates of cells, as well as their mitochondrial reserve capacity. Moreover, it suppressed the production of proteins linked to the endoplasmic reticulum stress response (Grp78, PERK, IRE1-α, and ATF6), which may be one mechanism by which it enhances I/R energy metabolism [11,169].

4.4 Restoring MQC

Mitochondrial fission, fusion, mitophagy, and biogenesis are potential targets for natural drugs in the treatment of I/R MQC disorders.

Baicalin reduced mitochondrial fission by inhibiting DRP1 synthesis, thereby protecting the myocardium [170]. Salidroside inhibited DRP1 phosphorylation and mitochondrial fission via the AMPK pathway [171]. Gyp XVII pretreatment increased MFN2 expression and promoted mitochondrial fusion [11]. By inhibiting excessive mitochondrial fission and promoting fusion, the natural substances vitexin and hirsutine, as well as the TCM formula Tongmai, upregulated the expression of the mitochondrial fusion protein MFN2 and downregulated the expression of the mitochondrial fission regulatory protein DRP1. Thus, they alleviated damage to the mitochondrial ultrastructure caused by H/R [10,12,172]. In H/R-treated H9c2 cardiomyocytes, crocetin decreased DRP1 expression by activating PGC-1α, which could be eliminated by blocking PGC-1α expression [75]. Therefore, PGC-1α participates in both cell mitochondrial fission and mitochondrial biogenesis [173], and is a potential candidate for the treatment of MQC.

Regarding mitophagy and mitochondrial biogenesis, *Panax Notoginseng* saponins significantly increased LC3 expression and LC3II/LC3I ratio in rat myocardium by activating the HIF-1α/BNIP3 signaling pathway [174]. The natural compounds gerontoxanthone I, macluraxanthone, and the TCM formula *Tongxinluo* activated mitophagy via the PINK1/Parkin pathway to degrade dysfunctional mitochondria and ameliorate I/R, both *in vitro* and *in vivo* [55,175]. Asiatic acid increased LC3II levels to promote mitophagy through the AMPK signaling pathway [176]. *Yiqi Huoxue* enhanced the LC3BII/LC3BI ratio and triggered mitophagy via the PINK1/Parkin and BNIP3/Nix pathways [177]. The ancient Chinese remedy *Shuangshen Ningxin capsule*, significantly suppressed the activity of PINK1, Parkin, FUNDC1, and BNIP3 in heart muscle cells of miniature pigs with I/R injury. Thus, *Shuangshen Ningxin capsules* exerted cardioprotective effects by inhibiting mitophagy [88]. Naringenin promoted the protein expression of NRF1, TFAM, and oxidative phosphorylation complex subunits II, III, and IV through the AMPK/SIRT3 signaling pathway, which enhanced mitochondrial biogenesis [178].

Shenmai injection stimulated the expression of PINK1, PARKIN, and LC3, which triggered mitophagy. Moreover, it promoted mitochondrial fusion and suppressed excessive fission. Consequently, *in vitro* mitochondrial homeostasis was preserved in cardiomyocytes subjected to H/R [157]. Hydroxysafflor yellow A inhibited mitochondrial fission by reducing the expression of MFF and FIS1 in the mitochondria and enhanced mitophagy by inducing the HIF-1α/BNIP3 signaling pathway [179]. Pretreatment with *Shen Yuan Dan*, a TCM formula, hindered mitophagy via the PINK1/Parkin pathway and suppressed the production of DRP1 and FIS1 mRNA, which in turn prevented mitochondrial fission. It also increased the levels of MFN1/2, OPA1, and PGC-1α mRNA and thereby encouraged mitochondrial

fusion and mitochondrial biogenesis [180].

In summary, encouraging mitochondrial fusion, controlling mitophagy, limiting excessive mitochondrial fission, and increasing mitochondrial biogenesis are all aspects of natural medication treatments for MQC. Mitophagy regulates myocardial cell death. The regulation of mitophagy by drugs varies during I/R. The precise regulation of mitophagy is a key issue that requires further research.

4.5 Inhibiting cell apoptosis or necroptosis

Bcl-2 is a target of mitochondria-mediated apoptosis. Bcl-2 activation prevents the activation of caspase-9 and caspase-3, which disrupt apoptosis by blocking the Bax-mediated release of mitochondrial cyt C. Astragaloside IV pretreatment significantly increased Bcl-2 levels in cardiomyocytes, thereby inhibiting apoptosis. The selective inhibition of Bcl-2 led to a decrease in its myocardial protective effects [127]. *In vitro* and *in vivo* experiments using geniposide, eriodictyol, and *YiQiFuMai powder injection* showed that they may decrease the expression of Bax and caspase-3 and increase the levels of Bcl-2 protein. The effect of Huangzhi Oral Liquid may be mediated by P53. The effect of *YiQiFuMai powder injection* was mediated by AMPK activation. The phosphoinositol 3-kinase (PI3K)/protein kinase B (Akt) signaling pathway mediated the effects of geniposide [139,140,155,181]. Ginsenosides Rd and Rc, *Panax quinquefolium* saponins, aqueous extracts of *Cortex Dictamni*, hydroxytyrosol, and asiatic acid have all been reported to increase the Bcl-2/Bax ratio, lower cyt C release, and prevent the activation of caspase-9 and caspase-3 after I/R damage [128,142,182–185]. The PI3K/Akt signaling pathway may be involved in the action of ginsenoside Rd and the aqueous extract of *Cortex Dictamni*. The action of ginsenoside Rc may be mediated by the activation of the SIRT1/forkhead box protein O1 (FOXO1)/Bcl-2 signaling pathway. And the action of asiatic acid has been shown to be mediated by the inhibition of the p38-mitogen-activated protein kinase (MAPK) and c-Jun N-terminal kinase (JNK)-MAPK signaling pathways in *in vitro* and *in vivo* experiments.

Bax is also a candidate inhibitor of apoptosis. 14-3-3 is a cytoplasmic Bax anchoring protein Phosphorylation of 14-3-3 leads to the dissociation of Bax from 14-3-3 by triggering the JNK signaling pathway. Bax is transported to the mitochondria to initiate apoptosis [186]. Research has shown that parishin J and B, the active ingredients of the plant *Gastrodia elata*, can reduce the phosphorylation of 14-3-3 proteins and increase their binding to Bax by downregulating the phosphorylation levels of JNK1 and its downstream transcription factors c-Jun and cyclic AMP-dependent transcription factor 2 (ATF2), which exert an inhibitory effect on H/R-induced cardiomyocyte apoptosis [187]. Schisandrol A, the active component of *Schisandra chinensis*, reduces cleaved caspase-3 expression and increases

Bcl-2/Bax ratio in addition to upregulating 14-3-3 β protein to mitigate myocardial damage. Furthermore, it easily binds to 14-3-3θ, which is its possible mechanism of action [188].

To reduce necroptosis in I/R cardiomyocytes, current research has mostly concentrated on the targeted effects of medicines on RIPK1, RIPK3, and MLKL, or the regulatory effects of the cascade signals constituting these three [189,190]. Arctiin suppressed the activation of RIPK1, p-RIPK1, RIPK3, p-RIPK3, MLKL, and p-MLKL both *in vivo* and *in vitro*, ultimately inhibiting necroptosis in cardiomyocytes. Furthermore, using the Molecular Operating Environment program, researchers have hypothesized that RIPK1 and MLKL are potential targets of arctiin [191]. After inhibiting p-RIP1, p-RIP3, and p-MLKL expression, tanshinone I demonstrated myocardial protective effects both *in vitro and in vivo* [144]. In H/R cardiomyocytes, ginsenoside Rg2 suppressed RIP1, RIP3, and MLKL phosphorylation and prevented RIP1/RIP3 complex formation [192]. Additionally, by interacting with lysosomal-associated membrane protein 2 (LAMP2), maslinic acid induced apoptosis by downregulating the expression of Bax, cleaved caspase-9 and caspase-3; increasing the expression of Bcl-2; and reducing the activity of the RIPK1/RIPK3/MLKL axis. Thus, maslinic acid inhibited both I/R cell apoptosis and necroptosis [193].

One strategy to delay cardiomyocyte death by apoptosis or necroptosis is to inhibit excessive opening of the mPTP during I/R. Thus, mPTP is not only a key link in the initiation of necroptosis but also a mediator of oxidative stress bursts and mitochondrial cyt C release in I/R injury [28,29]. A study demonstrated that by triggering the PI3K/Akt signaling pathway, intravenous tanshinone IIA injection prior to reperfusion reduced mitochondrial permeability transition [194]. Picroside II pretreatment reduced the degree of mPTP opening in H/R cardiomyocytes, increased MMP, and inhibited the release of mitochondrial cyt C [195]. Hydroxysafflor yellow A prevented mPTP opening in hypoxic cardiomyocytes, thereby protecting them from damage during reoxygenation [196]. Its cardioprotective effect was similar to that of cyclosporine A (inhibitor of mPTP opening) [197]. Rhynchophylline, the primary active component of *Uncaria rhynchophylla*, inhibited mitochondria-mediated cardiomyocyte apoptosis during oxygen-glucose depletion/reoxygenation (OGD/R) by downregulating the expression of caspase-3 and caspase-9 mRNA and cytoplasmic cyt C [156]. Sappanone A and curculigoside both prevented the mPTP from excessive opening, which in turn reduced the release of mitochondrial cyt C and the cleavage of caspase-9 and caspase-3 [198,199]. The protective effects of curculigoside were accompanied by decreased Apaf-1 expression. Sappanone A inhibited mPTP via the PI3K/Akt/glycogen synthase kinase 3 β (Gsk-3 β) signaling pathway.

5 Conclusion and perspectives

Myocardial I/R damage presents a challenge for medical professionals and researchers. Reperfusion, the most effective way to combat ischemic heart disease, can cause further and more severe injury to the damaged myocardium. Mitochondria operate as indispensable regulators of pathological alterations and targets of damage and functional loss. Ischemia and hypoxia affect mitochondrial energy metabolism, decrease ATP production, and impair mitochondrial function. The resupply of O_2 after reperfusion increases superoxide production and triggers an influx of Ca^{2+} into cardiomyocytes and mitochondria, further damaging mitochondrial structure and function. Disturbances occur in the MQC mechanism, loss of MMP, stimulation of mPTP opening, and release of cyt C into the cytoplasm. These modifications activate signaling pathways associated with cell fate, resulting in cell death. TCM preparations target mitochondria-mediated alterations and cell death processes to produce therapeutic benefits against I/R damage, as shown in Table 1 [10–12,48,55,75,88,127,128,134,135,135–144,149–159,161–172,174–185,187,188,191–196,198,199].

However, recent findings on the effect of TCM preparations on calcium overload during I/R are limited to observations, that is, changes in Ca^{2+} levels in cardiomyocytes or mitochondria. Few studies have explored the specific mechanisms involved. Therefore, the potential effects on the expression, activity, and structure of related calcium channel proteins should be further studied. Exploring the potential molecules or signaling pathways involved in the regulatory mechanism is a potential way to increase the understanding of the effect of TCM on calcium overload.

Owing to the complexity of their chemical compositions, the effects of TCM preparations on I/R are often multi-targeted and interwoven (Fig. 2). Although this is one of the benefits of using Chinese medicine for therapy, it also poses challenges for pharmacological studies and broad use in myocardial I/R damage. Therefore, few Chinese medicines have advanced to the point of clinical investigation for treating myocardial I/R damage. Further investigations are required to resolve this issue. More specifically, area for further research include the analysis of TCM composition, pharmacological research, toxicological research, screening of single active ingredients, and the development of new drugs. Component analysis techniques, such as high-performance liquid chromatography-mass spectrometry (HPLC-MS) and quality evaluation based on quality markers (Q-Markers), can be used to identify the active ingredients of TCM preparations. Identification and screening of TCM action targets can be performed using target analysis techniques, such as network pharmacology and molecular docking, or multi-omics high-throughput techniques, such as metabolomics and proteomics. Highly efficient extraction of Chinese medicinal monomers can be achieved using technologies such as macroporous resin adsorption and separation, supercritical fluid extraction, and ultrasonic extraction.

Moreover, pharmacokinetic and toxicological studies should be conducted to identify the effective monomer components with fewer adverse effects. In addition, new technologies, such as computer-aided drug design, nano- and microsphere modification, and nanodrug delivery systems, can be used to modify the active monomer structure and optimize the dosage form and administration route of TCM, which is a promising strategy for developing new efficient drugs for myocardial I/R injury. In this review, saponins and flavonoids have been identified as the main TCM monomers known to enhance mitochondrial abnormalities in myocardial I/R injury. While flavonoids are highly effective in scavenging free radicals, saponins primarily improve energy metabolism and suppress apoptosis. These compounds are widely found in plant-derived TCMs. In TCM theory, herbs high in flavonoids are thought to have the properties of "heat-clearing" and "activating blood," whereas herbs high in saponins are thought to have the benefits of "invigorating Qi" and "nourishing Yin." Therefore, screening for saponins and flavonoids from herbs with the above effects should be considered. This approach will provide a faster and more effective method to screen for effective monomer drugs and develop new drugs against myocardial I/R injury.

References

[1] A.N. Nowbar, M. Gitto, J.P. Howard, D.P. Francis, R. Al-Lamee, Mortality From Ischemic Heart Disease: Analysis of Data From the World Health Organization and Coronary Artery Disease Risk Factors From NCD Risk Factor Collaboration, Circ. Cardiovasc. Qual. Outcomes 12 (2019) e005375. https://doi.org/10.1161/CIRCOUTCOMES.118.005375.

[2] D.J. Hausenloy, H.E. Botker, T. Engstrom, D. Erlinge, G. Heusch, B. Ibanez, R.A. Kloner, M. Ovize, D.M. Yellon, D. Garcia-Dorado, Targeting reperfusion injury in patients with ST-segment elevation myocardial infarction: trials and tribulations, Eur. Heart J. (2016) ehw145. https://doi.org/10.1093/eurheartj/ehw145.

[3] S.M. Davidson, S. Arjun, M.V. Basalay, R.M. Bell, D.I. Bromage, H.E. Bøtker, R.D. Carr, J. Cunningham, A.K. Ghosh, G. Heusch, B. Ibanez, P. Kleinbongard, S. Lecour, H. Maddock, M. Ovize, M. Walker, M. Wiart, D.M. Yellon, The 10th Biennial Hatter Cardiovascular Institute workshop: cellular protection—evaluating new directions in the setting of myocardial infarction, ischaemic stroke, and cardio-oncology, Basic Res. Cardiol. 113 (2018) 43. https://doi.org/10.1007/s00395-018-0704-z.

[4] W.W. Brooks, C.H. Conrad, J.P. Morgan, Reperfusion induced arrhythmias following ischaemia in intact rat heart: role of intracellular calcium, Cardiovasc. Res. 29 (1995) 536–542.

[5] J. Wang, H. Zhou, Mitochondrial quality control mechanisms as molecular targets in cardiac ischemia–reperfusion injury, Acta Pharm. Sin. B 10 (2020) 1866–1879.

https://doi.org/10.1016/j.apsb.2020.03.004.
[6] D.P. Del Re, D. Amgalan, A. Linkermann, Q. Liu, R.N. Kitsis, Fundamental Mechanisms of Regulated Cell Death and Implications for Heart Disease, Physiol. Rev. 99 (2019) 1765–1817. https://doi.org/10.1152/physrev.00022.2018.
[7] Kuznetsov, Javadov, Margreiter, Grimm, Hagenbuchner, Ausserlechner, The Role of Mitochondria in the Mechanisms of Cardiac Ischemia-Reperfusion Injury, Antioxidants 8 (2019) 454. https://doi.org/10.3390/antiox8100454.
[8] E.J. Griffiths, Mitochondria and Heart Disease, Adv. Mitochondrial Med. 942 (2012) 249–267. https://doi.org/10.1007/978-94-007-2869-1_11.
[9] S. Antonucci, J.F. Mulvey, N. Burger, M. Di Sante, A.R. Hall, E.C. Hinchy, S.T. Caldwell, A.V. Gruszczyk, S. Deshwal, R.C. Hartley, N. Kaludercic, M.P. Murphy, F. Di Lisa, T. Krieg, Selective mitochondrial superoxide generation *in vivo* is cardioprotective through hormesis, Free Radic. Biol. Med. 134 (2019) 678–687. https://doi.org/10.1016/j.freeradbiomed.2019.01.034.
[10] Y. Zhao, R. Guo, L. Li, S. Li, G. Fan, X. Zhao, Y. Wang, Tongmai formula improves cardiac function via regulating mitochondrial quality control in the myocardium with ischemia/reperfusion injury, Biomed. Pharmacother. 132 (2020) 110897. https://doi.org/10.1016/j.biopha.2020.110897.
[11] S. Su, J. Wang, J. Wang, R. Yu, L. Sun, Y. Zhang, L. Song, W. Pu, Y. Tang, Y. Yu, K. Zhou, Cardioprotective effects of gypenoside XVII against ischemia/reperfusion injury: Role of endoplasmic reticulum stress, autophagy, and mitochondrial fusion fission balance, Phytother. Res. 36 (2022) 2982–2998. https://doi.org/10.1002/ptr.7493.
[12] W. Jiang, Y. Zhang, W. Zhang, X. Pan, J. Liu, Q. Chen, J. Chen, Hirsutine ameliorates myocardial ischemia-reperfusion injury through improving mitochondrial function via CaMKII pathway, Clin. Exp. Hypertens. 45 (2023) 2192444. https://doi.org/10.1080/10641963.2023.2192444.
[13] C.J.A. Ramachandra, S. Hernandez-Resendiz, G.E. Crespo-Avilan, Y.-H. Lin, D.J. Hausenloy, Mitochondria in acute myocardial infarction and cardioprotection, EBioMedicine 57 (2020) 102884. https://doi.org/10.1016/j.ebiom.2020.102884.
[14] Z. Yin, N. Burger, D. Kula-Alwar, D. Aksentijević, H.R. Bridges, H.A. Prag, D.N. Grba, C. Viscomi, A.M. James, A. Mottahedin, T. Krieg, M.P. Murphy, J. Hirst, Structural basis for a complex I mutation that blocks pathological ROS production, Nat. Commun. 12 (2021) 707. https://doi.org/10.1038/s41467-021-20942-w.
[15] K.-N. Shi, P.-B. Li, H.-X. Su, J. Gao, H.-H. Li, MK-886 protects against cardiac ischaemia/reperfusion injury by activating proteasome-Keap1-NRF2 signalling, Redox Biol. 62 (2023) 102706. https://doi.org/10.1016/j.redox.2023.102706.
[16] T. Wu, T. Jing, Y. Lu, F. Zhang, P. He, In Situ Investigation of Intercellular Signal Transduction Based on Detection of Extracellular pH and ROS by Scanning Electrochemical Microscopy, Anal. Chem. 95 (2023) 7468–7474.

https://doi.org/10.1021/acs.analchem.2c04655.

[17] E.T. Chouchani, V.R. Pell, E. Gaude, D. Aksentijević, S.Y. Sundier, E.L. Robb, A. Logan, S.M. Nadtochiy, E.N.J. Ord, A.C. Smith, F. Eyassu, R. Shirley, C.-H. Hu, A.J. Dare, A.M. James, S. Rogatti, R.C. Hartley, S. Eaton, A.S.H. Costa, P.S. Brookes, S.M. Davidson, M.R. Duchen, K. Saeb-Parsy, M.J. Shattock, A.J. Robinson, L.M. Work, C. Frezza, T. Krieg, M.P. Murphy, Ischaemic accumulation of succinate controls reperfusion injury through mitochondrial ROS, Nature 515 (2014) 431–435. https://doi.org/10.1038/nature13909.

[18] K.R. Pryde, J. Hirst, Superoxide Is Produced by the Reduced Flavin in Mitochondrial Complex I, J. Biol. Chem. 286 (2011) 18056–18065. https://doi.org/10.1074/jbc.M110.186841.

[19] E.L. Robb, A.R. Hall, T.A. Prime, S. Eaton, M. Szibor, C. Viscomi, A.M. James, M.P. Murphy, Control of mitochondrial superoxide production by reverse electron transport at complex I, J. Biol. Chem. 293 (2018) 9869–9879. https://doi.org/10.1074/jbc.RA118.003647.

[20] A.V. Gruszczyk, A.M. Casey, A.M. James, H.A. Prag, N. Burger, G.R. Bates, A.R. Hall, F.M. Allen, T. Krieg, K. Saeb-Parsy, M.P. Murphy, Mitochondrial metabolism and bioenergetic function in an anoxic isolated adult mouse cardiomyocyte model of *in vivo* cardiac ischemia-reperfusion injury, Redox Biol. 54 (2022) 102368. https://doi.org/10.1016/j.redox.2022.102368.

[21] N.I. Markevich, M.H. Galimova, L.N. Markevich, Hysteresis and bistability in the succinate-CoQ reductase activity and reactive oxygen species production in the mitochondrial respiratory complex II, Redox Biol. 37 (2020) 101630. https://doi.org/10.1016/j.redox.2020.101630.

[22] J.L. Martin, A.S.H. Costa, A.V. Gruszczyk, T.E. Beach, F.M. Allen, H.A. Prag, E.C. Hinchy, K. Mahbubani, M. Hamed, L. Tronci, E. Nikitopoulou, A.M. James, T. Krieg, A.J. Robinson, M.M. Huang, S.T. Caldwell, A. Logan, L. Pala, R.C. Hartley, C. Frezza, K. Saeb-Parsy, M.P. Murphy, Succinate accumulation drives ischaemia-reperfusion injury during organ transplantation, Nat. Metab. 1 (2019) 966–974. https://doi.org/10.1038/s42255-019-0115-y.

[23] A.S. Milliken, S.M. Nadtochiy, P.S. Brookes, Inhibiting Succinate Release Worsens Cardiac Reperfusion Injury by Enhancing Mitochondrial Reactive Oxygen Species Generation, J. Am. Heart Assoc. 11 (2022) e026135. https://doi.org/10.1161/JAHA.122.026135.

[24] R.D. Guzy, B. Hoyos, E. Robin, H. Chen, L. Liu, K.D. Mansfield, M.C. Simon, U. Hammerling, P.T. Schumacker, Mitochondrial complex III is required for hypoxia-induced ROS production and cellular oxygen sensing, Cell Metab. 1 (2005) 401–408. https://doi.org/10.1016/j.cmet.2005.05.001.

[25] N. Kuksal, D. Gardiner, D. Qi, R.J. Mailloux, Partial loss of complex I due to NDUFS4 deficiency augments myocardial reperfusion damage by increasing

mitochondrial superoxide/hydrogen peroxide production, Biochem. Biophys. Res. Commun. 498 (2018) 214–220. https://doi.org/10.1016/j.bbrc.2018.02.208.

[26] C.P. Baines, R.A. Kaiser, N.H. Purcell, N.S. Blair, H. Osinska, M.A. Hambleton, E.W. Brunskill, M.R. Sayen, R.A. Gottlieb, G.W. Dorn, J. Robbins, J.D. Molkentin, Loss of cyclophilin D reveals a critical role for mitochondrial permeability transition in cell death, Nature 434 (2005) 658–662. https://doi.org/10.1038/nature03434.

[27] J. Li, C. Bai, J. Guo, W. Liang, J. Long, NDUFA 4L2 protects against ischaemia/reperfusion-induced cardiomyocyte apoptosis and mitochondrial dysfunction by inhibiting complex I, Clin. Exp. Pharmacol. Physiol. 44 (2017) 779–786. https://doi.org/10.1111/1440-1681.12768.

[28] J. Park, J. Lee, C. Choi, Mitochondrial Network Determines Intracellular ROS Dynamics and Sensitivity to Oxidative Stress through Switching Inter-Mitochondrial Messengers, PLoS ONE 6 (2011) e23211. https://doi.org/10.1371/journal.pone.0023211.

[29] X. Li, P. Jia, Z. Huang, S. Liu, J. Miao, Y. Guo, N. Wu, D. Jia, Lycopene protects against myocardial ischemia-reperfusion injury by inhibiting mitochondrial permeability transition pore opening, Drug Des. Devel. Ther. Volume 13 (2019) 2331–2342. https://doi.org/10.2147/DDDT.S194753.

[30] Z. Todorović, S. Đurašević, M. Stojković, I. Grigorov, S. Pavlović, N. Jasnić, T. Tosti, J.B. Macut, C. Thiemermann, J. Đorđević, Lipidomics Provides New Insight into Pathogenesis and Therapeutic Targets of the Ischemia—Reperfusion Injury, Int. J. Mol. Sci. 22 (2021) 2798. https://doi.org/10.3390/ijms22062798.

[31] T.W. Dorey, M. Mackasey, H.J. Jansen, M.D. McRae, L.J. Bohne, Y. Liu, D.D. Belke, L. Atkinson, R.A. Rose, Natriuretic peptide receptor B maintains heart rate and sinoatrial node function via cyclic GMP-mediated signalling, Cardiovasc. Res. 118 (2022) 1917–1931. https://doi.org/10.1093/cvr/cvab245.

[32] L. Livshits, S. Peretz, A. Bogdanova, H. Zoabi, H. Eitam, G. Barshtein, C. Galindo, Y. Feldman, I. Pajić-Lijaković, A. Koren, M. Gassmann, C. Levin, The Impact of Ca2+ on Intracellular Distribution of Hemoglobin in Human Erythrocytes, Cells 12 (2023) 2280. https://doi.org/10.3390/cells12182280.

[33] L. Wehrli, I. Galdadas, L. Voirol, M. Smieško, Y. Cambet, V. Jaquet, S. Guerrier, F.L. Gervasio, S. Nef, R. Rahban, The action of physiological and synthetic steroids on the calcium channel CatSper in human sperm, Front. Cell Dev. Biol. 11 (2023) 1221578. https://doi.org/10.3389/fcell.2023.1221578.

[34] D. Sacks, B. Baxter, B.C.V. Campbell, J.S. Carpenter, C. Cognard, D. Dippel, M. Eesa, U. Fischer, K. Hausegger, J.A. Hirsch, M. Shazam Hussain, O. Jansen, M.V. Jayaraman, A.A. Khalessi, B.W. Kluck, S. Lavine, P.M. Meyers, S. Ramee, D.A. Rüfenacht, C.M. Schirmer, D. Vorwerk, Multisociety Consensus Quality Improvement Revised Consensus Statement for Endovascular Therapy of Acute Ischemic Stroke, Int. J. Stroke Off. J. Int. Stroke Soc. 13 (2018) 612–632.

https://doi.org/10.1177/1747493018778713.
[35]O. Lozano, A. Lázaro-Alfaro, C. Silva-Platas, Y. Oropeza-Almazán, A. Torres-Quintanilla, J. Bernal-Ramírez, H. Alves-Figueiredo, G. García-Rivas, Nanoencapsulated Quercetin Improves Cardioprotection during Hypoxia-Reoxygenation Injury through Preservation of Mitochondrial Function, Oxid. Med. Cell. Longev. 2019 (2019) 1–14. https://doi.org/10.1155/2019/7683051.
[36]Y.-C. Cui, C.-S. Pan, L. Yan, L. Li, B.-H. Hu, X. Chang, Y.-Y. Liu, J.-Y. Fan, K. Sun, Q. -Li, J.-Y. Han, Ginsenoside Rb1 protects against ischemia/reperfusion-induced myocardial injury via energy metabolism regulation mediated by RhoA signaling pathway, Sci. Rep. 7 (2017) 44579. https://doi.org/10.1038/srep44579.
[37]P. Alegre, L. Mathias, M.A. Lourenço, P.P.D. Santos, A. Gonçalves, A.A. Fernandes, P.S.A. Gaiolla, M.F. Minicucci, L. Zornoff, S.A.R. Paiva, B.F. Polegato, Euterpe Oleracea Mart. (Açaí) Reduces Oxidative Stress and Improves Energetic Metabolism in Myocardial Ischemia-Reperfusion Injury in Rats, Arq. Bras. Cardiol. 114 (2020) 78–86. https://doi.org/10.36660/abc.20180140.
[38]H. Liu, P.M. Cala, S.E. Anderson, Na/H Exchange Inhibition Protects Newborn Heart From Ischemia/Reperfusion Injury by Limiting Na+-dependent Ca2+ Overload, J. Cardiovasc. Pharmacol. 55 (2010) 227–233. https://doi.org/10.1097/FJC.0b013e3181cb599f.
[39]X. Lu, X. Bi, X. He, M. Zhao, M. Xu, X. Yu, Z. Zhao, W. Zang, Activation of M_3 cholinoceptors attenuates vascular injury after ischaemia/reperfusion by inhibiting the Ca^{2+} /calmodulin-dependent protein kinase II pathway, Br. J. Pharmacol. 172 (2015) 5619–5633. https://doi.org/10.1111/bph.13183.
[40]P. De Koninck, H. Schulman, Sensitivity of CaM Kinase II to the Frequency of Ca^{2+} Oscillations, Science 279 (1998) 227–230. https://doi.org/10.1126/science.279.5348.227.
[41]I. Namekata, H. Shimada, T. Kawanishi, H. Tanaka, K. Shigenobu, Reduction by SEA0400 of myocardial ischemia-induced cytoplasmic and mitochondrial Ca2+ overload, Eur. J. Pharmacol. 543 (2006) 108–115. https://doi.org/10.1016/j.ejphar.2006.06.012.
[42]G. González, D. Zaldívar, E. Carrillo, A. Hernández, M. García, J. Sánchez, Pharmacological preconditioning by diazoxide downregulates cardiac L-type Ca^{2+} channels, Br. J. Pharmacol. 161 (2010) 1172–1185. https://doi.org/10.1111/j.1476-5381.2010.00960.x.
[43]A.C. Pardo, L.A. Diaz Zegarra, L.F. González Arbeláez, A.M. Ibáñez, R.G. Díaz, E.A. Aiello, S.M. Mosca, Cardioprotective effects of N-methylacetazolamide mediated by inhibition of L-type Ca^{2+} channel current, Biochim. Biophys. Acta BBA - Gen. Subj. 1866 (2022) 130098. https://doi.org/10.1016/j.bbagen.2022.130098.
[44]T. Hiroi, T. Wajima, T. Negoro, M. Ishii, Y. Nakano, Y. Kiuchi, Y. Mori, S.

Shimizu, Neutrophil TRPM2 channels are implicated in the exacerbation of myocardial ischaemia/reperfusion injury, Cardiovasc. Res. 97 (2013) 271–281. https://doi.org/10.1093/cvr/cvs332.

[45] P. Sánchez-Pérez, A. Mata, M.-K. Torp, E. López-Bernardo, C.M. Heiestad, J.M. Aronsen, A. Molina-Iracheta, L.J. Jiménez-Borreguero, P. García-Roves, A.S.H. Costa, C. Frezza, M.P. Murphy, K.-O. Stenslokken, S. Cadenas, Energy substrate metabolism, mitochondrial structure and oxidative stress after cardiac ischemia-reperfusion in mice lacking UCP3, Free Radic. Biol. Med. 205 (2023) 244–261. https://doi.org/10.1016/j.freeradbiomed.2023.05.014.

[46] A. DuSablon, J. Parks, K. Whitehurst, H. Estes, R. Chase, E. Vlahos, U. Sharma, D. Wert, J. Virag, EphrinA1-Fc attenuates myocardial ischemia/reperfusion injury in mice, PLOS ONE 12 (2017) e0189307. https://doi.org/10.1371/journal.pone.0189307.

[47] P. Zheng, Z. Xie, Y. Yuan, W. Sui, C. Wang, X. Gao, Y. Zhao, F. Zhang, Y. Gu, P. Hu, J. Ye, X. Feng, L. Zhang, Plin5 alleviates myocardial ischaemia/reperfusion injury by reducing oxidative stress through inhibiting the lipolysis of lipid droplets, Sci. Rep. 7 (2017) 42574. https://doi.org/10.1038/srep42574.

[48] L. Tian, W. Cao, R. Yue, Y. Yuan, X. Guo, D. Qin, J. Xing, X. Wang, Pretreatment with Tilianin improves mitochondrial energy metabolism and oxidative stress in rats with myocardial ischemia/reperfusion injury via AMPK/SIRT1/PGC-1 alpha signaling pathway, J. Pharmacol. Sci. 139 (2019) 352–360. https://doi.org/10.1016/j.jphs.2019.02.008.

[49] E. Liepinsh, M. Makrecka-Kuka, J. Kuka, R. Vilskersts, E. Makarova, H. Cirule, E. Loza, D. Lola, S. Grinberga, O. Pugovics, I. Kalvins, M. Dambrova, Inhibition of L-carnitine biosynthesis and transport by methyl-γ-butyrobetaine decreases fatty acid oxidation and protects against myocardial infarction, Br. J. Pharmacol. 172 (2015) 1319–1332. https://doi.org/10.1111/bph.13004.

[50] A.R. Hall, Q.G. Karwi, S. Kumar, R. Dongworth, D. Aksentijević, T.R. Altamimi, K.T. Fridianto, K. Chinda, S. Hernandez-Resendiz, M.U. Mahmood, E. Michelakis, C.J. Ramachandra, J. Ching, J.M. Vicencio, M.J. Shattock, J.-P. Kovalik, D.M. Yellon, G. Lopaschuk, D.J. Hausenloy, Fasting increases susceptibility to acute myocardial ischaemia/reperfusion injury through a sirtuin-3 mediated increase in fatty acid oxidation, Sci. Rep. 12 (2022) 20551. https://doi.org/10.1038/s41598-022-23847-w.

[51] A. Prola, Z. Nichtova, J. Pires Da Silva, J. Piquereau, K. Monceaux, A. Guilbert, M. Gressette, R. Ventura-Clapier, A. Garnier, I. Zahradnik, M. Novotova, C. Lemaire, Endoplasmic reticulum stress induces cardiac dysfunction through architectural modifications and alteration of mitochondrial function in cardiomyocytes, Cardiovasc. Res. 115 (2019) 328–342. https://doi.org/10.1093/cvr/cvy197.

[52] P. Binder, B. Nguyen, L. Collins, M. Zi, W. Liu, F. Christou, X. Luo, S.S. Hille, N. Frey, E.J. Cartwright, J. Chernoff, O.J. Müller, K. Guan, X. Wang, Pak2 Regulation of Nrf2 Serves as a Novel Signaling Nexus Linking ER Stress Response and Oxidative

Stress in the Heart, Front. Cardiovasc. Med. 9 (2022) 851419. https://doi.org/10.3389/fcvm.2022.851419.

[53] C.T. Ronayne, T.D. Jackson, C.F. Bennett, E.A. Perry, N. Kantorovic, P. Puigserver, Tetracyclines activate mitoribosome quality control and reduce ER stress to promote cell survival, EMBO Rep. 24 (2023) e57228. https://doi.org/10.15252/embr.202357228.

[54] I. Tahmaz, S. Shahmoradi Ghahe, M. Stasiak, K.P. Liput, K. Jonak, U. Topf, Prefoldin 2 contributes to mitochondrial morphology and function, BMC Biol. 21 (2023) 193. https://doi.org/10.1186/s12915-023-01695-y.

[55] Q. Xiang, M. Wu, L. Zhang, W. Fu, J. Yang, B. Zhang, Z. Zheng, H. Zhang, Y. Lao, H. Xu, Gerontoxanthone I and Macluraxanthone Induce Mitophagy and Attenuate Ischemia/Reperfusion Injury, Front. Pharmacol. 11 (2020) 452. https://doi.org/10.3389/fphar.2020.00452.

[56] S.B. Kalkhoran, J. Kriston-Vizi, S. Hernandez-Resendiz, G.E. Crespo-Avilan, A.A. Rosdah, J.G. Lees, J.R.S.D. Costa, N.X.Y. Ling, J.K. Holien, P. Samangouei, K. Chinda, E.P. Yap, J.A. Riquelme, R. Ketteler, D.M. Yellon, S.Y. Lim, D.J. Hausenloy, Hydralazine protects the heart against acute ischaemia/reperfusion injury by inhibiting Drp1-mediated mitochondrial fission, Cardiovasc. Res. 118 (2022) 282–294. https://doi.org/10.1093/cvr/cvaa343.

[57] X. Li, X. Liu, W. Liu, Y. Lin, J. Liu, Y. Peng, L. Cheng, Y. Du, Inhibition of TMEM16A improves cisplatin-induced acute kidney injury via preventing DRP1-mediated mitochondrial fission, Acta Pharmacol. Sin. 44 (2023) 2230–2242. https://doi.org/10.1038/s41401-023-01122-6.

[58] Y. Tian, H. Ge, X. Bian, Y. Wang, Y. Lai, Y. Wang, Y. Bai, X. Zhang, J. Xu, W. Tian, FUNDC1: a key mediator of adenosine A2BR activation-induced inhibition of cardiac mitophagy under ischemia/reperfusion conditions, Cardiovasc. Diagn. Ther. 12 (2023) 509–522. https://doi.org/10.21037/cdt-22-468.

[59] D.-F. Suen, D.P. Narendra, A. Tanaka, G. Manfredi, R.J. Youle, Parkin overexpression selects against a deleterious mtDNA mutation in heteroplasmic cybrid cells, Proc. Natl. Acad. Sci. 107 (2010) 11835–11840. https://doi.org/10.1073/pnas.0914569107.

[60] L. Pirzeh, V. Babapour, R. Badalzadeh, N. Panahi, Pretreatment with vildagliptin boosts ischemic-postconditioning effects on cardioprotection and expression profile of genes regulating autophagy and mitochondrial fission/fusion in diabetic heart with reperfusion injury, Naunyn. Schmiedebergs Arch. Pharmacol. 392 (2019) 1371–1382. https://doi.org/10.1007/s00210-019-01660-z.

[61] J. Gao, H.-X. Su, P.-B. Li, K.-N. Shi, H.-H. Li, TCH-165 attenuates cardiac ischaemia/reperfusion injury by balancing mitochondrial dynamics via increasing proteasome activity, Eur. J. Pharmacol. 957 (2023) 176011. https://doi.org/10.1016/j.ejphar.2023.176011.

[62]J. Noone, K.D. Rochfort, F. O'Sullivan, D.J. O'Gorman, SIRT4 is a regulator of human skeletal muscle fatty acid metabolism influencing inner and outer mitochondrial membrane-mediated fusion, Cell. Signal. 112 (2023) 110931. https://doi.org/10.1016/j.cellsig.2023.110931.
[63]W. Ke, B. Wang, Z. Liao, Y. Song, G. Li, L. Ma, K. Wang, S. Li, W. Hua, C. Yang, Matrix stiffness induces Drp1-mediated mitochondrial fission through Piezo1 mechanotransduction in human intervertebral disc degeneration, J. Transl. Med. 21 (2023) 711. https://doi.org/10.1186/s12967-023-04590-w.
[64]H. Liu, H. Lin, T. Xu, X. Shi, Y. Yao, P.A. Khoso, Z. Jiang, S. Xu, New insights into brain injury in chickens induced by bisphenol A and selenium deficiency—Mitochondrial reactive oxygen species and mitophagy-apoptosis crosstalk homeostasis, Sci. Total Environ. 905 (2023) 166890. https://doi.org/10.1016/j.scitotenv.2023.166890.
[65]Z. Hu, H. Zhang, Y. Wang, B. Li, K. Liu, J. Ran, L. Li, Exercise activates Sirt1-mediated Drp1 acetylation and inhibits hepatocyte apoptosis to improve nonalcoholic fatty liver disease, Lipids Health Dis. 22 (2023) 33. https://doi.org/10.1186/s12944-023-01798-z.
[66]Q. Wang, H. Qiu, Deubiquitinase USP16 induces gouty arthritis via Drp1-dependent mitochondrial fission and NLRP3 inflammasome activation, Arthritis Res. Ther. 25 (2023) 126. https://doi.org/10.1186/s13075-023-03095-7.
[67]F. Consolato, F. Maltecca, S. Tulli, I. Sambri, G. Casari, *m* -AAA and *i* -AAA complexes work coordinately regulating OMA1, the stress-activated supervisor of mitochondrial dynamics, J. Cell Sci. (2018) jcs.213546. https://doi.org/10.1242/jcs.213546.
[68]Y. Jian, Y. Yang, L. Cheng, X. Yang, H. Liu, W. Li, Y. Wan, D. Yang, Sirt3 mitigates LPS -induced mitochondrial damage in renal tubular epithelial cells by deacetylating YME1L1, Cell Prolif. 56 (2023) e13362. https://doi.org/10.1111/cpr.13362.
[69]Y. Ge, S. Boopathy, T.H. Nguyen, C.M. Lugo, L.H. Chao, Absence of Cardiolipin From the Outer Leaflet of a Mitochondrial Inner Membrane Mimic Restricts Opa1-Mediated Fusion, Front. Mol. Biosci. 8 (2021) 769135. https://doi.org/10.3389/fmolb.2021.769135.
[70]W. Duan, C. Liu, J. Zhou, Q. Yu, Y. Duan, T. Zhang, Y. Li, G. Fu, Y. Sun, J. Tian, Z. Xia, Y. Yang, Y. Liu, S. Xu, Upregulation of mitochondrial calcium uniporter contributes to paraquat-induced neuropathology linked to Parkinson's disease via imbalanced OPA1 processing, J. Hazard. Mater. 453 (2023) 131369. https://doi.org/10.1016/j.jhazmat.2023.131369.
[71]H. Kim, M.C. Scimia, D. Wilkinson, R.D. Trelles, M.R. Wood, D. Bowtell, A. Dillin, M. Mercola, Z.A. Ronai, Fine-Tuning of Drp1/Fis1 Availability by AKAP121/Siah2 Regulates Mitochondrial Adaptation to Hypoxia, Mol. Cell 44 (2011)

532–544. https://doi.org/10.1016/j.molcel.2011.08.045.

[72] M. Disatnik, J.C.B. Ferreira, J.C. Campos, K.S. Gomes, P.M.M. Dourado, X. Qi, D. Mochly-Rosen, Acute Inhibition of Excessive Mitochondrial Fission After Myocardial Infarction Prevents Long-term Cardiac Dysfunction, J. Am. Heart Assoc. 2 (2013) e000461. https://doi.org/10.1161/JAHA.113.000461.

[73] J. Du, H. Li, J. Song, T. Wang, Y. Dong, A. Zhan, Y. Li, G. Liang, AMPK Activation Alleviates Myocardial Ischemia-Reperfusion Injury by Regulating Drp1-Mediated Mitochondrial Dynamics, Front. Pharmacol. 13 (2022) 862204. https://doi.org/10.3389/fphar.2022.862204.

[74] Y. Li, Z. Xiong, Y. Jiang, H. Zhou, L. Yi, Y. Hu, X. Zhai, J. Liu, F. Tian, Y. Chen, Klf4 deficiency exacerbates myocardial ischemia/reperfusion injury in mice via enhancing ROCK1/DRP1 pathway-dependent mitochondrial fission, J. Mol. Cell. Cardiol. 174 (2023) 115–132. https://doi.org/10.1016/j.yjmcc.2022.11.009.

[75] Y.-L. Mou, R. Zhao, S.-Y. Lyu, Z.-Y. Zhang, M.-F. Zhu, Q. Liu, Crocetin protects cardiomyocytes against hypoxia/reoxygenation injury by attenuating Drp1-mediated mitochondrial fission via PGC-1α, J. Geriatr. Cardiol. 20 (2023) 68–82. https://doi.org/10.26599/1671-5411.2023.01.001.

[76] P. Yu, J. Zhang, S. Yu, Z. Luo, F. Hua, L. Yuan, Z. Zhou, Q. Liu, X. Du, S. Chen, L. Zhang, G. Xu, Protective Effect of Sevoflurane Postconditioning against Cardiac Ischemia/Reperfusion Injury via Ameliorating Mitochondrial Impairment, Oxidative Stress and Rescuing Autophagic Clearance, PLOS ONE 10 (2015) e0134666. https://doi.org/10.1371/journal.pone.0134666.

[77] H.-H. Su, J.-M. Liao, Y.-H. Wang, K.-M. Chen, C.-W. Lin, I.-H. Lee, Y.-J. Li, J.-Y. Huang, S.K. Tsai, J.-C. Yen, S.-S. Huang, Exogenous GDF11 attenuates non-canonical TGF-β signaling to protect the heart from acute myocardial ischemia–reperfusion injury, Basic Res. Cardiol. 114 (2019) 20. https://doi.org/10.1007/s00395-019-0728-z.

[78] K.N. Papanicolaou, R.J. Khairallah, G.A. Ngoh, A. Chikando, I. Luptak, K.M. O'Shea, D.D. Riley, J.J. Lugus, W.S. Colucci, W.J. Lederer, W.C. Stanley, K. Walsh, Mitofusin-2 Maintains Mitochondrial Structure and Contributes to Stress-Induced Permeability Transition in Cardiac Myocytes, Mol. Cell. Biol. 31 (2011) 1309–1328. https://doi.org/10.1128/MCB.00911-10.

[79] Y. Chen, G. Csordás, C. Jowdy, T.G. Schneider, N. Csordás, W. Wang, Y. Liu, M. Kohlhaas, M. Meiser, S. Bergem, J.M. Nerbonne, G.W. Dorn, C. Maack, Mitofusin 2-Containing Mitochondrial-Reticular Microdomains Direct Rapid Cardiomyocyte Bioenergetic Responses Via Interorganelle Ca^{2+} Crosstalk, Circ. Res. 111 (2012) 863–875. https://doi.org/10.1161/CIRCRESAHA.112.266585.

[80] Y. Chen, M. Sparks, P. Bhandari, S.J. Matkovich, G.W. Dorn, Mitochondrial Genome Linearization Is a Causative Factor for Cardiomyopathy in Mice and *Drosophila*, Antioxid. Redox Signal. 21 (2014) 1949–1959.

https://doi.org/10.1089/ars.2013.5432.

[81] Y. Feng, A. Imam Aliagan, N. Tombo, J.C. Bopassa, Mitofilin Heterozygote Mice Display an Increase in Myocardial Injury and Inflammation after Ischemia/Reperfusion, Antioxidants 12 (2023) 921. https://doi.org/10.3390/antiox12040921.

[82] K.N. Papanicolaou, G.A. Ngoh, E.R. Dabkowski, K.A. O'Connell, R.F. Ribeiro, W.C. Stanley, K. Walsh, Cardiomyocyte deletion of mitofusin-1 leads to mitochondrial fragmentation and improves tolerance to ROS-induced mitochondrial dysfunction and cell death, Am. J. Physiol.-Heart Circ. Physiol. 302 (2012) H167–H179. https://doi.org/10.1152/ajpheart.00833.2011.

[83] A.R. Hall, N. Burke, R.K. Dongworth, S.B. Kalkhoran, A. Dyson, J.M. Vicencio, G.W. Dorn, D.M. Yellon, D.J. Hausenloy, Hearts deficient in both Mfn1 and Mfn2 are protected against acute myocardial infarction, Cell Death Dis. 7 (2016) e2238–e2238. https://doi.org/10.1038/cddis.2016.139.

[84] L. Guan, Z. Che, X. Meng, Y. Yu, M. Li, Z. Yu, H. Shi, D. Yang, M. Yu, MCU Up-regulation contributes to myocardial ischemia-reperfusion Injury through calpain/OPA-1–mediated mitochondrial fusion/mitophagy Inhibition, J. Cell. Mol. Med. 23 (2019) 7830–7843. https://doi.org/10.1111/jcmm.14662.

[85] J. Nan, C. Nan, J. Ye, L. Qian, Y. Geng, D. Xing, M.S.U. Rahman, M. Huang, EGCG protects cardiomyocytes against hypoxia-reperfusion injury via inhibiting OMA1 activation, J. Cell Sci. (2018) jcs.220871. https://doi.org/10.1242/jcs.220871.

[86] Y. Zhang, Y. Wang, J. Xu, F. Tian, S. Hu, Y. Chen, Z. Fu, Melatonin attenuates myocardial ischemia-reperfusion injury via improving mitochondrial fusion/mitophagy and activating the AMPK-OPA1 signaling pathways, J. Pineal Res. 66 (2019) e12542. https://doi.org/10.1111/jpi.12542.

[87] M. Ding, C. Liu, R. Shi, M. Yu, K. Zeng, J. Kang, F. Fu, M. Mi, Mitochondrial fusion promoter restores mitochondrial dynamics balance and ameliorates diabetic cardiomyopathy in an optic atrophy 1-dependent way, Acta Physiol. 229 (2020) e13428. https://doi.org/10.1111/apha.13428.

[88] F. Jia, Y. Chen, G. Xin, L. Li, Z. Liu, S. Xu, J. Gao, H. Meng, Y. Shi, Y. Ma, L. Li, J. Fu, Shuangshen Ningxin capsule alleviates myocardial ischemia–reperfusion injury in miniature pigs by modulating mitophagy: network pharmacology and experiments *in vivo*, Chin. Med. 18 (2023) 120. https://doi.org/10.1186/s13020-023-00810-z.

[89] L. Wang, T. Hilander, X. Liu, H.Y. Tsang, O. Eriksson, C.B. Jackson, M. Varjosalo, H. Zhao, GTPBP8 is required for mitoribosomal biogenesis and mitochondrial translation, Cell. Mol. Life Sci. 80 (2023) 361. https://doi.org/10.1007/s00018-023-05014-0.

[90] D. Wang, L. Yang, W. Ding, Z. Chen, X. Yang, Y. Jiang, Y. Liu, Licochalcone A alleviates abnormal glucolipid metabolism and restores energy homeostasis in diet-induced diabetic mice, Phytother. Res. (2023) ptr.8044.

https://doi.org/10.1002/ptr.8044.

[91]J. Fang, X. Zou, L. Gong, J. Xi, Y. Liu, X. Yang, X. Zhang, C. Gui, Acid ground nano-realgar processed product inhibits breast cancer by inducing mitophagy via the p53/ BNIP3 / NIX pathway, J. Cell. Mol. Med. 27 (2023) 3478–3490. https://doi.org/10.1111/jcmm.17917.

[92]K. Yang, J. Wu, S. Li, S. Wang, J. Zhang, Y. Wang, Y. Yan, H. Hu, M. Xiong, C. Bai, Y. Sun, W. Chen, Y. Zeng, J. Yuan, C. Yin, NTRK1 knockdown induces mouse cognitive impairment and hippocampal neuronal damage through mitophagy suppression via inactivating the AMPK/ULK1/FUNDC1 pathway, Cell Death Discov. 9 (2023) 404. https://doi.org/10.1038/s41420-023-01685-7.

[93]P.-Y. Ke, C.-W. Chang, Y.-C. Hsiao, Baicalein Activates Parkin-Dependent Mitophagy through NDP52 and OPTN, Cells 11 (2022) 1132. https://doi.org/10.3390/cells11071132.

[94]J. Wei, J. Xie, J. He, D. Li, D. Wei, Y. Li, X. Li, W. Fang, G. Wei, K. Lai, Active fraction of Polyrhachis vicina (Roger) alleviated cerebral ischemia/reperfusion injury by targeting SIRT3-mediated mitophagy and angiogenesis, Phytomedicine 121 (2023) 155104. https://doi.org/10.1016/j.phymed.2023.155104.

[95]F. Wang, J. Bai, X. Zhang, D. Wang, X. Zhang, J. Xue, H. Chen, S. Wang, B. Chi, J. Li, X. Ma, METTL3 / YTHDF2 м6A axis mediates the progression of diabetic nephropathy through epigenetically suppressing PINK1 and mitophagy, J. Diabetes Investig. (2023) jdi.14113. https://doi.org/10.1111/jdi.14113.

[96]C.T. Chu, J. Ji, R.K. Dagda, J.F. Jiang, Y.Y. Tyurina, A.A. Kapralov, V.A. Tyurin, N. Yanamala, I.H. Shrivastava, D. Mohammadyani, K.Z. Qiang Wang, J. Zhu, J. Klein-Seetharaman, K. Balasubramanian, A.A. Amoscato, G. Borisenko, Z. Huang, A.M. Gusdon, A. Cheikhi, E.K. Steer, R. Wang, C. Baty, S. Watkins, I. Bahar, H. Bayır, V.E. Kagan, Cardiolipin externalization to the outer mitochondrial membrane acts as an elimination signal for mitophagy in neuronal cells, Nat. Cell Biol. 15 (2013) 1197–1205. https://doi.org/10.1038/ncb2837.

[97]Y. Chang, W. Yan, X. He, L. Zhang, C. Li, H. Huang, G. Nace, D.A. Geller, J. Lin, A. Tsung, miR-375 Inhibits Autophagy and Reduces Viability of Hepatocellular Carcinoma Cells Under Hypoxic Conditions, Gastroenterology 143 (2012) 177-187.e8. https://doi.org/10.1053/j.gastro.2012.04.009.

[98]S. Zhu, X. Li, B. Dang, F. Wu, C. Wang, C. Lin, Lycium Barbarum polysaccharide protects HaCaT cells from PM2.5-induced apoptosis via inhibiting oxidative stress, ER stress and autophagy, Redox Rep. 27 (2022) 32–44. https://doi.org/10.1080/13510002.2022.2036507.

[99]R.C. Laker, J.C. Drake, R.J. Wilson, V.A. Lira, B.M. Lewellen, K.A. Ryall, C.C. Fisher, M. Zhang, J.J. Saucerman, L.J. Goodyear, M. Kundu, Z. Yan, Ampk phosphorylation of Ulk1 is required for targeting of mitochondria to lysosomes in exercise-induced mitophagy, Nat. Commun. 8 (2017) 548.

https://doi.org/10.1038/s41467-017-00520-9.

[100] C.-M. Hung, P.S. Lombardo, N. Malik, S.N. Brun, K. Hellberg, J.L. Van Nostrand, D. Garcia, J. Baumgart, K. Diffenderfer, J.M. Asara, R.J. Shaw, AMPK/ULK1-mediated phosphorylation of Parkin ACT domain mediates an early step in mitophagy, Sci. Adv. 7 (2021) eabg4544. https://doi.org/10.1126/sciadv.abg4544.

[101] S.A. Chae, M. Du, M.-J. Zhu, J.S. Son, Exercise enhances placental labyrinth trophoblast development by activation of PGC-1α and FNDC5/irisin, Biol. Reprod. (2023) ioad151. https://doi.org/10.1093/biolre/ioad151.

[102] Y. Koh, S. Lin, K. Hsu, K. Nagabhushanam, C. Ho, M. Pan, Pterostilbene Enhances Thermogenesis and Mitochondrial Biogenesis by Activating the SIRT1/PGC-1α/SIRT3 Pathway to Prevent Western Diet-Induced Obesity, Mol. Nutr. Food Res. 67 (2023) 2300370. https://doi.org/10.1002/mnfr.202300370.

[103] N. Gleyzer, K. Vercauteren, R.C. Scarpulla, Control of Mitochondrial Transcription Specificity Factors (TFB1M and TFB2M) by Nuclear Respiratory Factors (NRF-1 and NRF-2) and PGC-1 Family Coactivators, Mol. Cell. Biol. 25 (2005) 1354–1366. https://doi.org/10.1128/MCB.25.4.1354-1366.2005.

[104] D. Xiang, W. Yang, Z. Fang, J. Mao, Q. Yan, L. Li, J. Tan, C. Yu, J. Qian, D. Tang, X. Pan, H. Cheng, D. Sun, Agrimol B inhibits colon carcinoma progression by blocking mitochondrial function through the PGC-1α/NRF1/TFAM signaling pathway, Front. Oncol. 12 (2022) 1055126. https://doi.org/10.3389/fonc.2022.1055126.

[105] M. Kim, J. Kim, S. Moon, B.Y. Choi, S. Kim, H.S. Jeon, S.W. Suh, Y.-M. Kim, Y.K. Choi, Korean Red Ginseng Improves Astrocytic Mitochondrial Function by Upregulating HO-1-Mediated AMPKα–PGC-1α–ERRα Circuit after Traumatic Brain Injury, Int. J. Mol. Sci. 22 (2021) 13081. https://doi.org/10.3390/ijms222313081.

[106] Z. Li, Y. Zhang, Y. Zheng, W. Liu, X. Zhang, W. Li, D. Zhang, Q. Cai, S. Wang, X. Meng, G. Huang, Intranasal 15d-PGJ2 ameliorates brain glucose hypometabolism via PPARγ-dependent activation of PGC-1α/GLUT4 signalling in APP/PS1 transgenic mice, Neuropharmacology 196 (2021) 108685. https://doi.org/10.1016/j.neuropharm.2021.108685.

[107] L.J. McMeekin, K.L. Joyce, L.M. Jenkins, B.M. Bohannon, K.D. Patel, A.S. Bohannon, A. Patel, S.N. Fox, M.S. Simmons, J.J. Day, A. Kralli, D.K. Crossman, R.M. Cowell, Estrogen-related Receptor Alpha (ERRα) is Required for PGC-1α-dependent Gene Expression in the Mouse Brain, Neuroscience 479 (2021) 70–90. https://doi.org/10.1016/j.neuroscience.2021.10.007.

[108] Y. Tian, D. Shi, H. Liao, B. Lu, Z. Pang, The role of Huidouba in regulating skeletal muscle metabolic disorders in prediabetic mice through AMPK/PGC-1α/PPARα pathway, Diabetol. Metab. Syndr. 15 (2023) 145. https://doi.org/10.1186/s13098-023-01097-8.

[109] W. Bi, J. Jia, R. Pang, C. Nie, J. Han, Z. Ding, B. Liu, R. Sheng, J. Xu, J.

Zhang, Thyroid hormone postconditioning protects hearts from ischemia/reperfusion through reinforcing mitophagy, Biomed. Pharmacother. 118 (2019) 109220. https://doi.org/10.1016/j.biopha.2019.109220.

[110] S. Cao, Y. Sun, W. Wang, B. Wang, Q. Zhang, C. Pan, Q. Yuan, F. Xu, S. Wei, Y. Chen, Poly (ADP-ribose) polymerase inhibition protects against myocardial ischaemia/reperfusion injury via suppressing mitophagy, J. Cell. Mol. Med. 23 (2019) 6897–6906. https://doi.org/10.1111/jcmm.14573.

[111] A. Dhingra, R. Jayas, P. Afshar, M. Guberman, G. Maddaford, J. Gerstein, B. Lieberman, H. Nepon, V. Margulets, R. Dhingra, L.A. Kirshenbaum, Ellagic acid antagonizes Bnip3-mediated mitochondrial injury and necrotic cell death of cardiac myocytes, Free Radic. Biol. Med. 112 (2017) 411–422. https://doi.org/10.1016/j.freeradbiomed.2017.08.010.

[112] A.B. Morton, A.J. Smuder, M.P. Wiggs, S.E. Hall, B. Ahn, J.M. Hinkley, N. Ichinoseki-Sekine, A.M. Huertas, M. Ozdemir, T. Yoshihara, N.R. Wawrzyniak, S.K. Powers, Increased SOD2 in the diaphragm contributes to exercise-induced protection against ventilator-induced diaphragm dysfunction, Redox Biol. 20 (2019) 402–413. https://doi.org/10.1016/j.redox.2018.10.005.

[113] Q. Li, Y. Liu, Q. Huang, X. Yi, F. Qin, Z. Zhong, L. Lin, H. Yang, G. Gong, W. Wu, Hypoxia Acclimation Protects against Heart Failure Postacute Myocardial Infarction via Fundc1-Mediated Mitophagy, Oxid. Med. Cell. Longev. 2022 (2022) 1–14. https://doi.org/10.1155/2022/8192552.

[114] W. Liu, L. Huang, X. Liu, L. Zhu, Y. Gu, W. Tian, L. Zhang, S. Deng, T. Yu, Urocortin I Protects against Myocardial Ischemia/Reperfusion Injury by Sustaining Respiratory Function and Cardiolipin Content via Mitochondrial ATP-Sensitive Potassium Channel Opening, Oxid. Med. Cell. Longev. 2022 (2022) 1–20. https://doi.org/10.1155/2022/7929784.

[115] Y. Yan, L. Tian, Q. Jia, Y. Han, Y. Tian, H. Chen, S. Cui, J. Xi, Y. Yao, X. Zhao, MiR-130a-3p regulates FUNDC1-mediated mitophagy by targeting GJA1 in myocardial ischemia/reperfusion injury, Cell Death Discov. 9 (2023) 77. https://doi.org/10.1038/s41420-023-01372-7.

[116] K. Mao, K. Wang, M. Zhao, T. Xu, D.J. Klionsky, Two MAPK-signaling pathways are required for mitophagy in *Saccharomyces cerevisiae*, J. Cell Biol. 193 (2011) 755–767. https://doi.org/10.1083/jcb.201102092.

[117] J. Yun, R. Puri, H. Yang, M.A. Lizzio, C. Wu, Z.-H. Sheng, M. Guo, MUL1 acts in parallel to the PINK1/parkin pathway in regulating mitofusin and compensates for loss of PINK1/parkin, eLife 3 (2014) e01958. https://doi.org/10.7554/eLife.01958.

[118] T. Saito, J. Nah, S. Oka, R. Mukai, Y. Monden, Y. Maejima, Y. Ikeda, S. Sciarretta, T. Liu, H. Li, E. Baljinnyam, D. Fraidenraich, L. Fritzky, P. Zhai, S. Ichinose, M. Isobe, C.-P. Hsu, M. Kundu, J. Sadoshima, An alternative mitophagy pathway mediated by Rab9 protects the heart against ischemia, J. Clin. Invest. 129

(2019) 802–819. https://doi.org/10.1172/JCI122035.

[119] Q. Huang, H. Su, B. Qi, Y. Wang, K. Yan, X. Wang, X. Li, D. Zhao, A SIRT1 Activator, Ginsenoside Rc, Promotes Energy Metabolism in Cardiomyocytes and Neurons, J. Am. Chem. Soc. 143 (2021) 1416–1427. https://doi.org/10.1021/jacs.0c10836.

[120] X. Shi, Y. Li, Y. Wang, T. Ding, X. Zhang, N. Wu, Pharmacological postconditioning with sappanone A ameliorates myocardial ischemia reperfusion injury and mitochondrial dysfunction via AMPK-mediated mitochondrial quality control, Toxicol. Appl. Pharmacol. 427 (2021) 115668. https://doi.org/10.1016/j.taap.2021.115668.

[121] J. Du, P. Hang, Y. Pan, B. Feng, Y. Zheng, T. Chen, L. Zhao, Z. Du, Inhibition of miR-23a attenuates doxorubicin-induced mitochondria-dependent cardiomyocyte apoptosis by targeting the PGC-1α/Drp1 pathway, Toxicol. Appl. Pharmacol. 369 (2019) 73–81. https://doi.org/10.1016/j.taap.2019.02.016.

[122] J. Subramani, V. Kundumani-Sridharan, K.C. Das, Thioredoxin protects mitochondrial structure, function and biogenesis in myocardial ischemia-reperfusion via redox-dependent activation of AKT-CREB- PGC1α pathway in aged mice, Aging 12 (2020) 19809–19827. https://doi.org/10.18632/aging.104071.

[123] J. Yang, J. He, M. Ismail, S. Tweeten, F. Zeng, L. Gao, S. Ballinger, M. Young, S.D. Prabhu, G.C. Rowe, J. Zhang, L. Zhou, M. Xie, HDAC inhibition induces autophagy and mitochondrial biogenesis to maintain mitochondrial homeostasis during cardiac ischemia/reperfusion injury, J. Mol. Cell. Cardiol. 130 (2019) 36–48. https://doi.org/10.1016/j.yjmcc.2019.03.008.

[124] X. Xue, W. Xi, W. Li, J. Xiao, Z. Wang, Y. Zhang, Hydrogen-rich saline alleviates cardiomyocyte apoptosis by reducing expression of calpain1 via miR-124-3p, ESC Heart Fail. 10 (2023) 3077–3090. https://doi.org/10.1002/ehf2.14492.

[125] R. Salie, J. Lopes, L. Kotze, R. Van Aarde, The cardioprotective effect of S. africana caerulea/Blue Sage in ischaemia and reperfusion induced oxidative stress, Front. Pharmacol. 14 (2023) 1254561. https://doi.org/10.3389/fphar.2023.1254561.

[126] J. Fang, Y. Tang, X. Cheng, L. Wang, C. Cai, X. Zhang, S. Liu, P. Li, Exenatide alleviates adriamycin-induced heart dysfunction in mice: Modulation of oxidative stress, apoptosis and inflammation, Chem. Biol. Interact. 304 (2019) 186–193. https://doi.org/10.1016/j.cbi.2019.03.012.

[127] Y. Luo, Q. Wan, M. Xu, Q. Zhou, X. Chen, D. Yin, H. He, M. He, Nutritional preconditioning induced by astragaloside IV on isolated hearts and cardiomyocytes against myocardial ischemia injury via improving Bcl-2-mediated mitochondrial function, Chem. Biol. Interact. 309 (2019) 108723. https://doi.org/10.1016/j.cbi.2019.06.036.

[128] J. Miao, Z. Huang, S. Liu, X. Li, P. Jia, Y. Guo, N. Wu, D. Jia,

Hydroxytyrosol protects against myocardial ischemia reperfusion injury by inhibiting mitochondrial permeability transition pore opening, Exp. Ther. Med. (2018). https://doi.org/10.3892/etm.2018.7016.

[129] T. Nakagawa, S. Shimizu, T. Watanabe, O. Yamaguchi, K. Otsu, H. Yamagata, H. Inohara, T. Kubo, Y. Tsujimoto, Cyclophilin D-dependent mitochondrial permeability transition regulates some necrotic but not apoptotic cell death, Nature 434 (2005) 652–658. https://doi.org/10.1038/nature03317.

[130] P. Zhu, S. Hu, Q. Jin, D. Li, F. Tian, S. Toan, Y. Li, H. Zhou, Y. Chen, Ripk3 promotes ER stress-induced necroptosis in cardiac IR injury: A mechanism involving calcium overload/XO/ROS/mPTP pathway, Redox Biol. 16 (2018) 157–168. https://doi.org/10.1016/j.redox.2018.02.019.

[131] P. Xiao, C. Wang, J. Li, H. Su, L. Yang, P. Wu, M.T. Lewno, J. Liu, X. Wang, COP9 Signalosome Suppresses RIPK1-RIPK3–Mediated Cardiomyocyte Necroptosis in Mice, Circ. Heart Fail. 13 (2020) e006996. https://doi.org/10.1161/CIRCHEARTFAILURE.120.006996.

[132] D. Pozzer, E. Varone, A. Chernorudskiy, S. Schiarea, S. Missiroli, C. Giorgi, P. Pinton, M. Canato, E. Germinario, L. Nogara, B. Blaauw, E. Zito, A maladaptive ER stress response triggers dysfunction in highly active muscles of mice with SELENON loss, Redox Biol. 20 (2019) 354–366. https://doi.org/10.1016/j.redox.2018.10.017.

[133] T. Frank, M. Tuppi, M. Hugle, V. Dötsch, S.J.L. Van Wijk, S. Fulda, Cell cycle arrest in mitosis promotes interferon-induced necroptosis, Cell Death Differ. 26 (2019) 2046–2060. https://doi.org/10.1038/s41418-019-0298-5.

[134] Y. Du, K.M. Ko, Effects of emodin treatment on mitochondrial ATP generation capacity and antioxidant components as well as susceptibility to ischemia–reperfusion injury in rat hearts: Single versus multiple doses and gender difference, Life Sci. 77 (2005) 2770–2782. https://doi.org/10.1016/j.lfs.2005.03.027.

[135] Y. Du, K.M. Ko, Effects of pharmacological preconditioning by emodin/oleanolic acid treatment and/or ischemic preconditioning on mitochondrial antioxidant components as well as the susceptibility to ischemia-reperfusion injury in rat hearts, Mol. Cell. Biochem. 288 (2006) 135–142. https://doi.org/10.1007/s11010-006-9129-3.

[136] M. Zhou, H. Ren, J. Han, W. Wang, Q. Zheng, D. Wang, Protective Effects of Kaempferol against Myocardial Ischemia/Reperfusion Injury in Isolated Rat Heart via Antioxidant Activity and Inhibition of Glycogen Synthase Kinase-3 β, Oxid. Med. Cell. Longev. 2015 (2015) 1–8. https://doi.org/10.1155/2015/481405.

[137] Y. Yang, W. Duan, Y. Lin, W. Yi, Z. Liang, J. Yan, N. Wang, C. Deng, S. Zhang, Y. Li, W. Chen, S. Yu, D. Yi, Z. Jin, SIRT1 activation by curcumin pretreatment attenuates mitochondrial oxidative damage induced by myocardial ischemia reperfusion injury, Free Radic. Biol. Med. 65 (2013) 667–679.

https://doi.org/10.1016/j.freeradbiomed.2013.07.007.

[138] J. Yang, J. Han, Y. Li, B. Dong, Esculetin inhibits the apoptosis in H9c2 cardiomyocytes via the MAPK signaling pathway following hypoxia/reoxygenation injury, Biomed. Pharmacother. 88 (2017) 1206–1210. https://doi.org/10.1016/j.biopha.2017.01.126.

[139] X. Ran, J.X. Diao, X.G. Sun, M. Wang, H. An, G.Q. Huang, X.S. Zhao, W.X. Ma, F.H. Zhou, Y.G. Yang, C.M. Miao, *Huangzhi Oral Liquid* Prevents Arrhythmias by Upregulating Caspase-3 and Apoptosis Network Proteins in Myocardial Ischemia-Reperfusion Injury in Rats, Evid. Based Complement. Alternat. Med. 2015 (2015) 1–11. https://doi.org/10.1155/2015/518926.

[140] Y.-Q. Jiang, G. Chang, Y. Wang, D.-Y. Zhang, L. Cao, J. Liu, Geniposide Prevents Hypoxia/Reoxygenation-Induced Apoptosis in H9c2 Cells: Improvement of Mitochondrial Dysfunction and Activation of GLP-1R and the PI3K/AKT Signaling Pathway, Cell. Physiol. Biochem. 39 (2016) 407–421. https://doi.org/10.1159/000445634.

[141] Q. Li, L. Shen, Z. Wang, H.-P. Jiang, L.-X. Liu, Tanshinone IIA protects against myocardial ischemia reperfusion injury by activating the PI3K/Akt/mTOR signaling pathway, Biomed. Pharmacother. 84 (2016) 106–114. https://doi.org/10.1016/j.biopha.2016.09.014.

[142] L. Li, Y. Zhou, Y. Li, L. Wang, L. Sun, L. Zhou, H. Arai, Y. Qi, Y. Xu, Aqueous extract of *Cortex Dictamni* protects H9c2 cardiomyocytes from hypoxia/reoxygenation-induced oxidative stress and apoptosis by PI3K/Akt signaling pathway, Biomed. Pharmacother. 89 (2017) 233–244. https://doi.org/10.1016/j.biopha.2017.02.013.

[143] X. Chen, Q. Xie, Y. Zhu, J. Xu, G. Lin, S. Liu, Z. Su, X. Lai, Q. Li, J. Xie, X. Yang, Cardio-protective effect of tetrahydrocurcumin, the primary hydrogenated metabolite of curcumin *in vivo* and *in vitro*: Induction of apoptosis and autophagy via PI3K/AKT/mTOR pathways, Eur. J. Pharmacol. 911 (2021) 174495. https://doi.org/10.1016/j.ejphar.2021.174495.

[144] Y. Zhuo, R. Yuan, X. Chen, J. He, Y. Chen, C. Zhang, K. Sun, S. Yang, Z. Liu, H. Gao, Tanshinone I exerts cardiovascular protective effects *in vivo* and *in vitro* through inhibiting necroptosis via Akt/Nrf2 signaling pathway, Chin. Med. 16 (2021) 48. https://doi.org/10.1186/s13020-021-00458-7.

[145] Q. Chen, S. Moghaddas, C.L. Hoppel, E.J. Lesnefsky, Reversible Blockade of Electron Transport during Ischemia Protects Mitochondria and Decreases Myocardial Injury following Reperfusion, J. Pharmacol. Exp. Ther. 319 (2006) 1405–1412. https://doi.org/10.1124/jpet.106.110262.

[146] C. Methner, E.T. Chouchani, G. Buonincontri, V.R. Pell, S.J. Sawiak, M.P. Murphy, T. Krieg, Mitochondria selective *S* -nitrosation by mitochondria-targeted *S* -nitrosothiol protects against post-infarct heart failure in mouse hearts, Eur. J. Heart

Fail. 16 (2014) 712–717. https://doi.org/10.1002/ejhf.100.

[147] L. Valls-Lacalle, I. Barba, E. Miró-Casas, M. Ruiz-Meana, A. Rodríguez-Sinovas, D. García-Dorado, Selective Inhibition of Succinate Dehydrogenase in Reperfused Myocardium with Intracoronary Malonate Reduces Infarct Size, Sci. Rep. 8 (2018) 2442. https://doi.org/10.1038/s41598-018-20866-4.

[148] Y.-P. Zhao, F. Wang, W. Jiang, J. Liu, B.-L. Liu, L.-W. Qi, W. Zhou, A mitochondrion-targeting tanshinone IIA derivative attenuates myocardial hypoxia reoxygenation injury through a SDH-dependent antioxidant mechanism, J. Drug Target. 27 (2019) 896–902. https://doi.org/10.1080/1061186X.2019.1566338.

[149] J. Li, Y.-L. Yang, L.-Z. Li, L. Zhang, Q. Liu, K. Liu, P. Li, B. Liu, L.-W. Qi, Succinate accumulation impairs cardiac pyruvate dehydrogenase activity through GRP91-dependent and independent signaling pathways: Therapeutic effects of ginsenoside Rb1, Biochim. Biophys. Acta BBA - Mol. Basis Dis. 1863 (2017) 2835–2847. https://doi.org/10.1016/j.bbadis.2017.07.017.

[150] M. Chen, X. Wang, B.O. Hu, J. Zhou, X. Wang, W. Wei, H. Zhou, Ursolic acid stimulates UCP2 expression and protects H9c2 cells from hypoxia-reoxygenation injury via p38 signaling, J. Biosci. 43 (2018) 857–865.

[151] J. Li, W.-T. Chang, C.-Q. Li, C. Lee, H.-H. Huang, C.-W. Hsu, W.-J. Chen, X. Zhu, C.-Z. Wang, T.L. Vanden Hoek, Z.-H. Shao, Baicalein Preventive Treatment Confers Optimal Cardioprotection by PTEN/Akt/NO Activation, Am. J. Chin. Med. 45 (2017) 987–1001. https://doi.org/10.1142/S0192415X17500525.

[152] J. Li, W.-T. Chang, G. Qin, K.R. Wojcik, C.-Q. Li, C.-W. Hsu, M. Han, X. Zhu, T.L. Vanden Hoek, Z.-H. Shao, Baicalein Preconditioning Cardioprotection Involves Pro-Oxidant Signaling and Activation of Pyruvate Dehydrogenase, Am. J. Chin. Med. 50 (2022) 1255–1267. https://doi.org/10.1142/S0192415X22500513.

[153] W.-T. Chang, C.-Q. Li, C.-W. Hsu, C. Lee, H.-H. Huang, C.-S. Yuan, W.-J. Chen, T.L. Vanden Hoek, Z.-H. Shao, J. Li, Baicalein Cardioprotection via Oxidant Scavenging and Akt-Nitric Oxide Signaling: Identification of Early Reperfusion Phase as the Critical Therapeutic Window, Am. J. Chin. Med. 47 (2019) 1043–1056. https://doi.org/10.1142/S0192415X19500538.

[154] K.G. Yamazaki, A.Y. Andreyev, P. Ortiz-Vilchis, S. Petrosyan, A.S. Divakaruni, S.E. Wiley, C. De La Fuente, G. Perkins, G. Ceballos, F. Villarreal, A.N. Murphy, Intravenous (−)-epicatechin reduces myocardial ischemic injury by protecting mitochondrial function, Int. J. Cardiol. 175 (2014) 297–306. https://doi.org/10.1016/j.ijcard.2014.05.009.

[155] Y. Xie, R. Ji, M. Han, Eriodictyol protects H9c2 cardiomyocytes against the injury induced by hypoxia/reoxygenation by improving the dysfunction of mitochondria, Exp. Ther. Med. (2018). https://doi.org/10.3892/etm.2018.6918.

[156] Q. Qin, L. Cui, P. Li, Y. Wang, X. Zhang, M. Guo, Rhynchophylline ameliorates myocardial ischemia/reperfusion injury through the modulation of

mitochondrial mechanisms to mediate myocardial apoptosis, Mol. Med. Rep. (2019). https://doi.org/10.3892/mmr.2019.9908.

[157] J. Yu, Y. Li, X. Liu, Z. Ma, S. Michael, J.O. Orgah, G. Fan, Y. Zhu, Mitochondrial dynamics modulation as a critical contribution for Shenmai injection in attenuating hypoxia/reoxygenation injury, J. Ethnopharmacol. 237 (2019) 9–19. https://doi.org/10.1016/j.jep.2019.03.033.

[158] Q. Tu, Q. Jiang, M. Xu, Y. Jiao, H. He, S. He, W. Zheng, EGCG decreases myocardial infarction in both I/R and MIRI rats through reducing intracellular Ca2+ and increasing TnT levels in cardiomyocytes, Adv. Clin. Exp. Med. 30 (2021) 607–616. https://doi.org/10.17219/acem/134021.

[159] L. Testai, A. Martelli, A. Marino, V. D'Antongiovanni, F. Ciregia, L. Giusti, A. Lucacchini, S. Chericoni, M.C. Breschi, V. Calderone, The activation of mitochondrial BK potassium channels contributes to the protective effects of naringenin against myocardial ischemia/reperfusion injury, Biochem. Pharmacol. 85 (2013) 1634–1643. https://doi.org/10.1016/j.bcp.2013.03.018.

[160] T. Zhang, Y. Zhang, M. Cui, L. Jin, Y. Wang, F. Lv, Y. Liu, W. Zheng, H. Shang, J. Zhang, M. Zhang, H. Wu, J. Guo, X. Zhang, X. Hu, C.-M. Cao, R.-P. Xiao, CaMKII is a RIP3 substrate mediating ischemia- and oxidative stress–induced myocardial necroptosis, Nat. Med. 22 (2016) 175–182. https://doi.org/10.1038/nm.4017.

[161] H. Jiang, J. Xing, J. Fang, L. Wang, Y. Wang, L. Zeng, Z. Li, R. Liu, Tilianin Protects against Ischemia/Reperfusion-Induced Myocardial Injury through the Inhibition of the Ca^{2+}/Calmodulin-Dependent Protein Kinase II-Dependent Apoptotic and Inflammatory Signaling Pathways, BioMed Res. Int. 2020 (2020) 1–18. https://doi.org/10.1155/2020/5939715.

[162] J. Wen, Z. Wei, Z. Yuxiang, Y. Hao, P. Xiaomei, C. Qiang, C. Junhui, Tilianin extracted from Xiangqinglan (Herba Dracocephali Moldovicae) inhibits apoptosis induced by mitochondrial pathway and endoplasmic reticulum stress in H9c2 cells after oxygen-glucose deprivation/reoxygenation, J. Tradit. Chin. Med. Chung Tsa Chih Ying Wen Pan 43 (2023) 42–50. https://doi.org/10.19852/j.cnki.jtcm.20221111.001.

[163] S.-Q. Lin, X.-H. Wei, P. Huang, Y.-Y. Liu, N. Zhao, Q. Li, C.-S. Pan, B.-H. Hu, X. Chang, J.-Y. Fan, X.-Y. Yang, C.-S. Wang, H.-N. Liu, J.-Y. Han, *QiShenYiQi* Pills® prevents cardiac ischemia–reperfusion injury via energy modulation, Int. J. Cardiol. 168 (2013) 967–974. https://doi.org/10.1016/j.ijcard.2012.10.042.

[164] J. Chen, J. Wei, L. Wang, Y. Zhu, L. Li, M. Akinyi, X. Gao, G. Fan, Cardioprotection against ischemia/reperfusion injury by *QiShenYiQi* Pill via ameliorate of multiple mitochondrial dysfunctions, Drug Des. Devel. Ther. (2015) 3051. https://doi.org/10.2147/DDDT.S82146.

[165] K. He, L. Yan, C.-S. Pan, Y.-Y. Liu, Y.-C. Cui, B.-H. Hu, X. Chang, Q. Li, K.

Sun, X.-W. Mao, J.-Y. Fan, J.-Y. Han, ROCK-dependent ATP5D modulation contributes to the protection of notoginsenoside NR1 against ischemia-reperfusion-induced myocardial injury, Am. J. Physiol.-Heart Circ. Physiol. 307 (2014) H1764–H1776. https://doi.org/10.1152/ajpheart.00259.2014.
[166] Y. Yuan, W. Cao, Y. Hong, X. Guo, Y. Wang, Y. Wang, X. Wang, P. Hu, Tilianin pretreatment prevents myocardial ischemia-reperfusion injury via preservation of mitochondrial function in rat heart, Phytomedicine 34 (2017) 106–114. https://doi.org/10.1016/j.phymed.2017.08.007.
[167] R. Liu, X. Chang, J. Li, Y. Shunyu, *Zishen Huoxue* Recipe Protecting Mitochondrial Function of Hypoxic/Reoxygenated Myocardial Cells through mTORC1 Signaling Pathway, Evid. Based Complement. Alternat. Med. 2020 (2020) 1–16. https://doi.org/10.1155/2020/8327307.
[168] H. Yu, Q. Guan, L. Guo, H. Zhang, X. Pang, Y. Cheng, X. Zhang, Y. Sun, Gypenosides alleviate myocardial ischemia-reperfusion injury via attenuation of oxidative stress and preservation of mitochondrial function in rat heart, Cell Stress Chaperones 21 (2016) 429–437. https://doi.org/10.1007/s12192-016-0669-5.
[169] Y. Yu, M. Wang, R. Chen, X. Sun, G. Sun, X. Sun, Gypenoside XVII protects against myocardial ischemia and reperfusion injury by inhibiting ER stress–induced mitochondrial injury, J. Ginseng Res. 45 (2021) 642–653. https://doi.org/10.1016/j.jgr.2019.09.003.
[170] J. Wu, H. Chen, J. Qin, N. Chen, S. Lu, J. Jin, Y. Li, Baicalin Improves Cardiac Outcome and Survival by Suppressing Drp1-Mediated Mitochondrial Fission after Cardiac Arrest-Induced Myocardial Damage, Oxid. Med. Cell. Longev. 2021 (2021) 1–14. https://doi.org/10.1155/2021/8865762.
[171] X. Tian, Y. Huang, X. Zhang, R. Fang, Y. Feng, W. Zhang, L. Li, T. Li, Salidroside attenuates myocardial ischemia/reperfusion injury via AMPK-induced suppression of endoplasmic reticulum stress and mitochondrial fission, Toxicol. Appl. Pharmacol. 448 (2022) 116093. https://doi.org/10.1016/j.taap.2022.116093.
[172] W. Xue, X. Wang, H. Tang, F. Sun, H. Zhu, D. Huang, L. Dong, Vitexin attenuates myocardial ischemia/reperfusion injury in rats by regulating mitochondrial dysfunction induced by mitochondrial dynamics imbalance, Biomed. Pharmacother. 124 (2020) 109849. https://doi.org/10.1016/j.biopha.2020.109849.
[173] M. Ding, N. Feng, D. Tang, J. Feng, Z. Li, M. Jia, Z. Liu, X. Gu, Y. Wang, F. Fu, J. Pei, Melatonin prevents Drp1-mediated mitochondrial fission in diabetic hearts through SIRT1-PGC1α pathway, J. Pineal Res. 65 (2018) e12491. https://doi.org/10.1111/jpi.12491.
[174] X.-W. Liu, M.-K. Lu, H.-T. Zhong, L.-H. Wang, Y.-P. Fu, *Panax Notoginseng* Saponins Attenuate Myocardial Ischemia-Reperfusion Injury Through the HIF-1α/BNIP3 Pathway of Autophagy, J. Cardiovasc. Pharmacol. 73 (2019) 92–99. https://doi.org/10.1097/FJC.0000000000000640.

[175] H. Yang, P. Wang, N. Wang, S. Li, M. Yang, *Tongxinluo* Ameliorates Myocardial Ischemia-Reperfusion Injury Mainly via Activating Parkin-Mediated Mitophagy and Downregulating Ubiquitin-Proteasome System, Chin. J. Integr. Med. 27 (2021) 542–550. https://doi.org/10.1007/s11655-019-3166-8.
[176] F. Qiu, Y. Yuan, W. Luo, Y. Gong, Z. Zhang, Z. Liu, L. Gao, Asiatic acid alleviates ischemic myocardial injury in mice by modulating mitophagy- and glycophagy-based energy metabolism, Acta Pharmacol. Sin. 43 (2022) 1395–1407. https://doi.org/10.1038/s41401-021-00763-9.
[177] M. Chen, G. Zhong, M. Liu, H. He, J. Zhou, J. Chen, M. Zhang, Q. Liu, G. Tong, J. Luan, H. Zhou, Integrating network analysis and experimental validation to reveal the mitophagy-associated mechanism of *Yiqi Huoxue* (YQHX) prescription in the treatment of myocardial ischemia/reperfusion injury, Pharmacol. Res. 189 (2023) 106682. https://doi.org/10.1016/j.phrs.2023.106682.
[178] L.-M. Yu, X. Dong, X.-D. Xue, J. Zhang, Z. Li, H.-J. Wu, Z.-L. Yang, Y. Yang, H.-S. Wang, Naringenin improves mitochondrial function and reduces cardiac damage following ischemia-reperfusion injury: the role of the AMPK-SIRT3 signaling pathway, Food Funct. 10 (2019) 2752–2765. https://doi.org/10.1039/C9FO00001A.
[179] S. Cheng, H. Chen, Q. Fang, Y. Wang, S. Liu, Preventive Effects of Hydroxysafflor Yellow A on Hypoxia/Reoxygenation-Induced Injury in Rat Cardiomyocytes by Promoting the HIF-1α/BNIP3 Signaling Pathway-Mediated Mitophagy, J. Biol. Regul. Homeost. Agents 37 (2023) 1473–1484. https://doi.org/10.23812/j.biol.regul.homeost.agents.20233703.146.
[180] Z. Zhang, M. Zhou, H. Liu, W. Liu, J. Chen, Protective effects of Shen Yuan Dan on myocardial ischemia-reperfusion injury via the regulation of mitochondrial quality control, Cardiovasc. Diagn. Ther. 13 (2023) 395–407. https://doi.org/10.21037/cdt-23-86.
[181] F. Li, X. Zheng, X. Fan, K. Zhai, Y. Tan, J. Kou, B. Yu, YiQiFuMai Powder Injection Attenuates Ischemia/Reperfusion-Induced Myocardial Apoptosis Through AMPK Activation, Rejuvenation Res. 19 (2016) 495–508. https://doi.org/10.1089/rej.2015.1801.
[182] Y. Wang, X. Li, X. Wang, W. Lau, Y. Wang, Y. Xing, X. Zhang, X. Ma, F. Gao, Ginsenoside Rd Attenuates Myocardial Ischemia/Reperfusion Injury via Akt/GSK-3β Signaling and Inhibition of the Mitochondria-Dependent Apoptotic Pathway, PLoS ONE 8 (2013) e70956. https://doi.org/10.1371/journal.pone.0070956.
[183] D. Li, M. Liu, T.-Q. Tao, D.-D. Song, X.-H. Liu, D.-Z. Shi, *Panax Quinquefolium* Saponin Attenuates Cardiomyocyte Apoptosis and Opening of the Mitochondrial Permeability Transition Pore in a Rat Model of Ischemia/Reperfusion, Cell. Physiol. Biochem. 34 (2014) 1413–1426. https://doi.org/10.1159/000366347.
[184] C. Yi, M. Song, L. Sun, L. Si, D. Yu, B. Li, P. Lu, W. Wang, X. Wang, Asiatic

Acid Alleviates Myocardial Ischemia-Reperfusion Injury by Inhibiting the ROS-Mediated Mitochondria-Dependent Apoptosis Pathway, Oxid. Med. Cell. Longev. 2022 (2022) 1–16. https://doi.org/10.1155/2022/3267450.

[185] Y. Xue, W. Fu, P. Yu, Y. Li, X. Yu, H. Xu, D. Sui, Ginsenoside Rc Alleviates Myocardial Ischemia-Reperfusion Injury by Reducing Mitochondrial Oxidative Stress and Apoptosis: Role of SIRT1 Activation, J. Agric. Food Chem. 71 (2023) 1547–1561. https://doi.org/10.1021/acs.jafc.2c06926.

[186] F. Tsuruta, J. Sunayama, Y. Mori, S. Hattori, S. Shimizu, Y. Tsujimoto, K. Yoshioka, N. Masuyama, Y. Gotoh, JNK promotes Bax translocation to mitochondria through phosphorylation of 14-3-3 proteins, EMBO J. 23 (2004) 1889–1899. https://doi.org/10.1038/sj.emboj.7600194.

[187] Q. Wang, Z. Li, D. Wang, S. Yang, Y. Feng, Myocardial protection properties of parishins from the roots of Gastrodia elata Bl, Biomed. Pharmacother. 121 (2020) 109645. https://doi.org/10.1016/j.biopha.2019.109645.

[188] S. Gong, J. Liu, S. Wan, W. Yang, Y. Zhang, B. Yu, F. Li, J. Kou, Schisandrol A Attenuates Myocardial Ischemia/Reperfusion-Induced Myocardial Apoptosis through Upregulation of 14-3-3θ, Oxid. Med. Cell. Longev. 2021 (2021) 1–15. https://doi.org/10.1155/2021/5541753.

[189] M. Naseroleslami, N.M. Niri, I. Akbarzade, M. Sharifi, N. Aboutaleb, Simvastatin-loaded nano-niosomes confer cardioprotection against myocardial ischemia/reperfusion injury, Drug Deliv. Transl. Res. 12 (2022) 1423–1432. https://doi.org/10.1007/s13346-021-01019-z.

[190] Z. Li, R. Dai, M. Chen, L. Huang, K. Zhu, M. Li, W. Zhu, Y. Li, N. Xie, J. Li, L. Wang, F. Lan, C.-M. Cao, p55γ degrades RIP3 via MG53 to suppress ischaemia-induced myocardial necroptosis and mediates cardioprotection of preconditioning, Cardiovasc. Res. 119 (2023) 2421–2440. https://doi.org/10.1093/cvr/cvad123.

[191] H. Chen, L.-J. Tang, H. Tu, Y.-J. Zhou, N.-S. Li, X.-J. Luo, J. Peng, Arctiin protects rat heart against ischemia/reperfusion injury via a mechanism involving reduction of necroptosis, Eur. J. Pharmacol. 875 (2020) 173053. https://doi.org/10.1016/j.ejphar.2020.173053.

[192] Y. Li, H. Hao, H. Yu, L. Yu, H. Ma, H. Zhang, Ginsenoside Rg2 Ameliorates Myocardial Ischemia/Reperfusion Injury by Regulating TAK1 to Inhibit Necroptosis, Front. Cardiovasc. Med. 9 (2022) 824657. https://doi.org/10.3389/fcvm.2022.824657.

[193] L. Li, L. Lin, S. Lei, S. Shi, C. Chen, Z. Xia, Maslinic Acid Inhibits Myocardial Ischemia–Reperfusion Injury-Induced Apoptosis and Necroptosis via Promoting Autophagic Flux, DNA Cell Biol. 41 (2022) 487–497. https://doi.org/10.1089/dna.2021.0918.

[194] X. Yuan, S. Jing, L. Wu, L. Chen, J. Fang, Pharmacological postconditioning with tanshinone IIA attenuates myocardial ischemia-reperfusion injury in rats by

activating the phosphatidylinositol 3-kinase pathway, Exp. Ther. Med. 8 (2014) 973–977. https://doi.org/10.3892/etm.2014.1820.
[195] J.-Z. Li, S.-Y. Yu, D. Mo, X.-N. Tang, Q.-R. Shao, Picroside II inhibits hypoxia/reoxygenation-induced cardiomyocyte apoptosis by ameliorating mitochondrial function through a mechanism involving a decrease in reactive oxygen species production, Int. J. Mol. Med. 35 (2015) 446–452. https://doi.org/10.3892/ijmm.2014.2009.
[196] G. Huber, S. Priest, T. Geisbuhler, Cardioprotective Effect of Hydroxysafflor Yellow A via the Cardiac Permeability Transition Pore, Planta Med. 84 (2018) 507–518. https://doi.org/10.1055/s-0043-122501.
[197] C. Zhang, Y. Cheng, D. Liu, M. Liu, H. Cui, B. Zhang, Q. Mei, S. Zhou, Mitochondria-targeted cyclosporin A delivery system to treat myocardial ischemia reperfusion injury of rats, J. Nanobiotechnology 17 (2019) 18. https://doi.org/10.1186/s12951-019-0451-9.
[198] X. Shi, G. Tao, L. Ji, G. Tian, Sappanone A alleviates hypoxia/reoxygenation-induced cardiomyocytes injury through inhibition of mitochondrial apoptosis and activation of PI3K–Akt–Gsk-3β pathway, Biosci. Rep. 40 (2020) BSR20192442. https://doi.org/10.1042/BSR20192442.
[199] Y. Zhao, Y. Guo, Y. Chen, S. Liu, N. Wu, D. Jia, Curculigoside attenuates myocardial ischemia-reperfusion injury by inhibiting the opening of the mitochondrial permeability transition pore, Int. J. Mol. Med. (2020). https://doi.org/10.3892/ijmm.2020.4513.

Fig. 1. Mechanisms of myocardial I/R injury. IMS: intermembrane space; IMM: inner membrane; RET: reverse electron transport; ΔP: protonmotive force; NHE: Na^+/H^+-exchanger; NCX: Na^+/Ca^{2+}-exchanger; L-VDCC: L-type voltage-dependent Ca^{2+} channel; MQC: mitochondrial quality control; mPTP: mitochondrial permeability transition pore; Cyt C: cytochrome C.

Fig. 2. Mitochondria-mediated pathological processes and the targets of Chinese herbal medicine in myocardial I/R. ① Increase antioxidant levels: emodin, oleanolic acid, kaempferol, tanshinone IIA and I, esculetin, geniposide, curcumin, tilianin, tetrahydrocurcumin, aqueous extract of *Cortex Dictamni* and Huangzhi oral liquid. ② Reduce succinate accumulation: ginsenoside Rb1. ③ Dissipate the proton gradient: ursolic acid. ④ Trigger low level of protective ROS and eliminate excess ROS: baicalein. ⑤ Inhibit calcium overload: (-)-epicatechin, (-)-epigallocatechin-3-gallate, eriodictyol, rhynchophylline, hirsutine, naringenin and Shenmai injection. ⑥ Inhibit CaMKII activation: tilianin and hirsutine. ⑦ Promote mitochondrial respiration: (-)-epicatechin, gypenoside and gypenoside XVII. ⑧ Promote glucose metabolism:

(-)-epicatechin and ginsenoside Rb1. ⑨ Reduce fatty acid β-oxidation: ginsenoside Rb1 and Zishen Huoxue recipe. ⑩ Increase ATP production: notoginsenoside NR1, tilianin, *QiShenYiQi pill* and *Zishen Huoxue recipe*. ⑪ Inhibit excessive mitochondrial fission: baicalin, salidroside, crocetin, vitexin, hirsutine, hydroxysafflor yellow A, *Shenmai injection*, *Tongmai formula* and *Shen Yuan Dan*. ⑫ Enhance mitochondrial fusion: gypenoside XVII, vitexin, hirsutine, *Shenmai injection*, *Tongmai formula*, and *Shen Yuan Dan*. ⑬ Promote mitophagy: gerontoxanthone I, macluraxanthone, asiatic acid, *Panax Notoginseng* saponins, hydroxysafflor yellow A, *Yiqi Huoxue prescription*, *Tongxinluo*, and *Shenmai injection*. ⑭ Reduce mitophagy: *Shuangshen Ningxin capsule* and *Shen Yuan Dan*. ⑮ Promote mitochondrial biogenesis: naringenin and *Shen Yuan Dan*. ⑯ Increase the level of Bcl-2: astragaloside IV, geniposide, eriodictyol, maslinic acid, ginsenosides Rd and Rc, *Panax quinquefolium* saponin, aqueous extract of *Cortex Dictamni*, hydroxytyrosol, asiatic acid, *YiQiFuMai powder injection* and *Huangzhi oral liquid*. ⑰ Reduce the activation of Bax: parishin J and B and schisandrol A. ⑱ Dowregulate the expression of RIPK1, RIPK3 and MLKL: arctiin, tanshinone I, ginsenoside Rg2 and maslinic acid. ⑲ Inhibit the excessive opening of mPTP: tanshinone IIA, picroside II, hydroxysafflor yellow A, rhynchophylline, curculigoside and sappanone A. NHE: Na^+/H^+-exchanger; NCX: Na^+/Ca^{2+}-exchanger; L-VDCC: L-type voltage-dependent Ca^{2+} channel; MFF: mitochondrial fission factor; MID49/51: mitochondrial dynamic protein 49/51 kDa; FIS1: mitochondrial fission 1 protein; DRP1: dynamin-related protein 1; MFN1/2: mitofusin 1/2; OPA1: optic atrophy 1; S-OPA1: short isomer of OPA1; L-OPA1: long isomer of OPA1; NRF1/2: nuclear respiratory factor 1/2; PPARs: peroxisome proliferator-activated receptors; ERRs: estrogen-related receptors; PGC1-α: peroxisome proliferator-activated receptor γ coactivator 1α; LC3II: microtubule-associated protein 1A/1B light chain 3 II; TNF-R1: tumor necrosis factor receptor type I; TRAIL-R: TNF-related apoptosis-inducing ligand receptor; TRADD: TNF receptor type 1 related death domain protein; FADD: Fas-related death domain protein; RIPK1/3: receptor-interacting serine/threonine-protein kinases 1/2;CaMKII: Ca^{2+}/calmodulin-dependent protein kinase II; MCU: mitochondrial Ca^{2+} uniporter; MLKL: mixed linear kinase domain like protein; Cyt C: Cytochrome C; Apaf-1: apoptotic protease activator factor-1; MOMP: mitochondrial outer membrane permeability; mPTP: Mitochondrial permeability transition pore; $\Delta\Psi m$ (MMP): mitochondrial membrane potential; ROS: reactive oxygen species; RET: reverse electron transport; ΔP: protonmotive force; CoQ: ubiquinone; UQ•-, ubisemiquinone; PINK1: PTEN-induced putative kinase 1; TBK1: TANK-binding kinase 1; FUNDC1: FUN14 domain containing 1; BNIP3: BCL2/adenovirus E1B 19kDa protein interacting protein 3; NIX: NIP3 like protein X; NDP52: nuclear dot protein; OPTN: optopurin.

20240426163610981274636548206592

ORIGINALITY REPORT

21%

SIMILARITY INDEX

PRIMARY SOURCES

1	www.hindawi.com<br>Internet	101 words — 1%
2	Jin Wang, Hao Zhou. "Mitochondrial quality control mechanisms as molecular targets in cardiac ischemia-reperfusion injury", Acta Pharmaceutica Sinica B, 2020<br>Crossref	72 words — 1%
3	www.frontiersin.org<br>Internet	61 words — 1%
4	www.science.gov<br>Internet	60 words — 1%
5	link.springer.com<br>Internet	55 words — 1%
6	www.mdpi.com<br>Internet	46 words — 1%
7	www.imrpress.com<br>Internet	42 words — 1%
8	Tianjiao Zhao, Wei Wu, Lihua Sui, Qiong Huang, Yayun Nan, Jianhua Liu, Kelong Ai. "Reactive oxygen species-based nanomaterials for the treatment of myocardial ischemia reperfusion injuries", Bioactive Materials, 2021	33 words — < 1%

Crossref

9	qmro.qmul.ac.uk<br>Internet	30 words — < 1%
10	biotm.cis.udel.edu<br>Internet	24 words — < 1%
11	www.researchgate.net<br>Internet	23 words — < 1%
12	"Modulation of Oxidative Stress in Heart Disease", Springer Science and Business Media LLC, 2019<br>Crossref	20 words — < 1%
13	Xin Su, Mingyang Zhou, Yingjian Li, Jianzhen Zhang et al. "Protective effects of natural products against myocardial ischemia/reperfusion: Mitochondria-targeted therapeutics", Biomedicine & Pharmacotherapy, 2022<br>Crossref	20 words — < 1%
14	www.annualreviews.org<br>Internet	20 words — < 1%
15	Cong Chen, Lin-Tong Yu, Bai-Ru Cheng, Jiang-Lin Xu et al. "Promising Therapeutic Candidate for Myocardial Ischemia/Reperfusion Injury: What Are the Possible Mechanisms and Roles of Phytochemicals?", Frontiers in Cardiovascular Medicine, 2022<br>Crossref	19 words — < 1%
16	Jia Li, Yi-Lin Yang, Lan-Zhu Li, Lei Zhang, Qun Liu, Kang Liu, Ping Li, Baolin Liu, Lian-Wen Qi. "Succinate accumulation impairs cardiac pyruvate dehydrogenase activity through GRP91-dependent and	19 words — < 1%

independent signaling pathways: Therapeutic effects of ginsenoside Rb1", Biochimica et Biophysica Acta (BBA) - Molecular Basis of Disease, 2017
Crossref

---

17 Thaise Boeing, Francislaine Aparecida dos Reis Lívero, Priscila de Souza, Danielle Ayr Tavares de Almeida et al. "Natural Products as Modulators of Mitochondrial Dysfunctions Associated with Cardiovascular Diseases: Advances and Opportunities", Journal of Medicinal Food, 2023 — 19 words — < 1%
Crossref

---

18 "Anti-inflammatory Nutraceuticals and Chronic Diseases", Springer Science and Business Media LLC, 2016 — 17 words — < 1%
Crossref

---

19 Yutong Zhou, Wendong Suo, Xinai Zhang, Jiaojiao Liang, Weizhe Zhao, Yue Wang, Hong Li, Qing Ni. "Targeting mitochondrial quality control for diabetic cardiomyopathy: Therapeutic potential of hypoglycemic drugs", Biomedicine & Pharmacotherapy, 2023 — 17 words — < 1%
Crossref

---

20 idoc.pub — 17 words — < 1%
Internet

---

21 Fuminori Tsuruta, Jun Sunayama, Yasunori Mori, Seisuke Hattori et al. "JNK promotes Bax translocation to mitochondria through phosphorylation of 14-3-3 proteins", The EMBO Journal, 2004 — 16 words — < 1%
Crossref

---

22 Shuangyu Lv, Xiaotian Li, Shizhen Zhao, Huiyang Liu, Honggang Wang. "The Role of the Signaling Pathways Involved in the Protective Effect of Exogenous — 16 words — < 1%

Hydrogen Sulfide on Myocardial Ischemia-Reperfusion Injury", Frontiers in Cell and Developmental Biology, 2021
Crossref

---

23 worldwidescience.org — 16 words — < 1%
Internet

---

24 Xing Chang, Yukun Li, Chen Cai, Feng Wu, Jing He, Yaoyuan Zhang, Jiankai Zhong, Ying Tan, Ruxiu Liu, Hang Zhu, Hao Zhou. "Mitochondrial quality control mechanisms as molecular targets in diabetic heart", Metabolism, 2022 — 15 words — < 1%
Crossref

---

25 Bin Zeng, Lei Liu, Xiaoting Liao, Caixia Zhang, Huaiyu Ruan. "Thyroid hormone protects cardiomyocytes from H2O2-induced oxidative stress via the PI3K-AKT signaling pathway", Experimental Cell Research, 2019 — 14 words — < 1%
Crossref

---

26 Jenq-Lin Yang, Sujira Mukda, Shang-Der Chen. "Diverse roles of mitochondria in ischemic stroke", Redox Biology, 2018 — 14 words — < 1%
Crossref

---

27 Xiang Ao, Wei Ding, Xiaoge Li, Qingling Xu, Xinhui Chen, Xuehao Zhou, Jianxun Wang, Ying Liu. "Non-coding RNAs regulating mitochondrial function in cardiovascular diseases", Journal of Molecular Medicine, 2023 — 14 words — < 1%
Crossref

---

28 Xin Tian, Ye Huang, Xiaofeng Zhang, Rong Fang, Yi Feng, Wanfang Zhang, Ling Li, Tian Li. "Salidroside attenuates myocardial ischemia/reperfusion injury via AMPK-induced suppression of endoplasmic reticulum stress and mitochondrial fission", Toxicology and Applied Pharmacology, 2022 — 14 words — < 1%
Crossref

29 aging-us.com
Internet
14 words — < 1%

30 Asish Dasgupta, Danchen Wu, Lian Tian, Ping Yu Xiong et al. "Mitochondria in the Pulmonary Vasculature in Health and Disease: Oxygen-Sensing, Metabolism, and Dynamics", Wiley, 2020
Crossref
13 words — < 1%

31 Youngil Lee, Insu Kwon, Yongchul Jang, Wankeun Song, Ludmila M. Cosio-Lima, Mark H. Roltsch. "Potential signaling pathways of acute endurance exercise-induced cardiac autophagy and mitophagy and its possible role in cardioprotection", The Journal of Physiological Sciences, 2017
Crossref
13 words — < 1%

32 Biomathematical and Biomechanical Modeling of the Circulatory and Ventilatory Systems, 2015.
Crossref
12 words — < 1%

33 www.alice.cnptia.embrapa.br
Internet
12 words — < 1%

34 Jinjin Chen, Qingxia Huang, Jing Li, Yao Yao et al. "Panax ginseng against myocardial ischemia/reperfusion injury: A review of preclinical evidence and potential mechanisms", Journal of Ethnopharmacology, 2022
Crossref
11 words — < 1%

35 Laura Doblado, Claudia Lueck, Claudia Rey, Alejandro K. Samhan-Arias, Ignacio Prieto, Alessandra Stacchiotti, Maria Monsalve. "Mitophagy in Human Diseases", International Journal of Molecular Sciences, 2021
Crossref
11 words — < 1%

36 Qian, Y.. "Maslinic acid, a natural triterpenoid compound from Olea europaea, protects cortical neurons against oxygen-glucose deprivation-induced injury", European Journal of Pharmacology, 20111116 — 11 words — < 1%
Crossref

37 Tie Hu, Fa-Jia Hu, Huang Huang, Ze-Yu Zhang, Ya-Mei Qiao, Wen-Xiong Huang, Yi-Cheng Wang, Xin-Yi Tang, Song-Qing Lai. "Epigallocatechin-3-gallate confers protection against myocardial ischemia/reperfusion injury by inhibiting ferroptosis, apoptosis, and autophagy via modulation of 14–3-3η", Biomedicine & Pharmacotherapy, 2024 — 11 words — < 1%
Crossref

38 circres.ahajournals.org — 11 words — < 1%
Internet

39 cmjournal.biomedcentral.com — 11 words — < 1%
Internet

40 medworm.com — 11 words — < 1%
Internet

41 pubs.kist.re.kr — 11 words — < 1%
Internet

42 www.scilit.net — 11 words — < 1%
Internet

43 www.spandidos-publications.com — 11 words — < 1%
Internet

44 Dan Qiao, Jingchun Jin, Jian Xing, Yingying Zhang et al. "Baicalein Inhibits Gastric Cancer Cell Proliferation and Migration through a FAK Interaction via — 10 words — < 1%

AKT/mTOR Signaling", The American Journal of Chinese Medicine, 2021
Crossref

---

45 Dexiang Xia, Yue Liu, Peng Wu, Dangheng Wei. "Current Advances of Mitochondrial Dysfunction and Cardiovascular Disease and Promising Therapeutic Strategies", The American Journal of Pathology, 2023 — 10 words — < 1%
Crossref

---

46 Tao Zhang, Yingli Nie, Jiliang Wang. "The emerging significance of mitochondrial targeted strategies in NAFLD treatment", Life Sciences, 2023 — 10 words — < 1%
Crossref

---

47 Yi-Han Lin, Maryann P. Platt, Ryan P. Gilley, David Brown, Peter H. Dube, Yanbao Yu, Norberto Gonzalez-Juarbe. "Influenza Causes MLKL-Driven Cardiac Proteome Remodeling During Convalescence", Circulation Research, 2021 — 10 words — < 1%
Crossref

---

48 www.physiology.org — 10 words — < 1%
Internet

---

49 Epiphani C. Simmons, Natalie E. Scholpa, Rick G. Schnellmann. "Mitochondrial biogenesis as a therapeutic target for traumatic and neurodegenerative CNS diseases", Experimental Neurology, 2020 — 9 words — < 1%
Crossref

---

50 Fatemeh Babaei, Armita Moafizad, Zahra Darvishvand, Mohammadreza Mirzababaei, Hossein Hosseinzadeh, Marjan Nassiri-Asl. "Review of the effects of vitexin in oxidative stress-related diseases", Food Science & Nutrition, 2020 — 9 words — < 1%
Crossref

51 He Tai, Xiao-lin Jiang, Nan Song, Hong-he Xiao et al. "Tanshinone IIA Combined With Cyclosporine A Alleviates Lung Apoptosis Induced by Renal Ischemia-Reperfusion in Obese Rats", Frontiers in Medicine, 2021 — 9 words — < 1%
Crossref

52 Hong Wu, Haixia Gao, Shuibo Gao, Zhen Lei, Liping Dai, Xinzhou Wang, Yongjun Han, Zhentao Wang, Lihua Han. "A Chinese 4-herb formula, Yiqi-Huoxue granule, alleviates H2O2-induced apoptosis by upregulating uncoupling protein 2 in H9c2 cells", Phytomedicine, 2019 — 9 words — < 1%
Crossref

53 Je~ek, P.. "Distinctions and similarities of cell bioenergetics and the role of mitochondria in hypoxia, cancer, and embryonic development", International Journal of Biochemistry and Cell Biology, 201005 — 9 words — < 1%
Crossref

54 Jing-Yan Han, Quan Li, Zhi-Zhong Ma, Jing-Yu Fan. "Effects and mechanisms of compound Chinese medicine and major ingredients on microcirculatory dysfunction and organ injury induced by ischemia/reperfusion", Pharmacology & Therapeutics, 2017 — 9 words — < 1%
Crossref

55 Pasquale Pagliaro, Francesca Moro, Francesca Tullio, Maria-Giulia Perrelli, Claudia Penna. "Cardioprotective Pathways During Reperfusion: Focus on Redox Signaling and Other Modalities of Cell Signaling", Antioxidants & Redox Signaling, 2011 — 9 words — < 1%
Crossref

56 Yaping Yang, Ye Tian, Xiaosu Guo, Shiping Li, Weiping Wang, Jiong Shi. "Ischemia Injury induces mPTP opening by reducing Sirt3", Neuroscience, 2021 — 9 words — < 1%
Crossref

57 Yong Luo, Qing Wan, Min Xu, Qing Zhou, Xuepiao Chen, Dong Yin, Huan He, Ming He. "Nutritional preconditioning induced by astragaloside IV on isolated hearts and cardiomyocytes against myocardial ischemia injury via improving Bcl-2-mediated mitochondrial function", Chemico-Biological Interactions, 2019
Crossref
9 words — < 1%

58 Yueyang Li, Zhenyu Xiong, Yufan Jiang, Hao Zhou, Li Yi, Yingyun Hu, Xiaofeng Zhai, Jie Liu, Feng Tian, Yundai Chen. "Klf4 deficiency exacerbates myocardial ischemia/reperfusion injury in mice via enhancing ROCK1/DRP1 pathway-dependent mitochondrial fission", Journal of Molecular and Cellular Cardiology, 2023
Crossref
9 words — < 1%

59 Zhuhua Zhang, Mingxue Zhou, Hongxu Liu, Wei Liu, Jiaping Chen. "Protective effects of Shen Yuan Dan on myocardial ischemia-reperfusion injury via the regulation of mitochondrial quality control", Cardiovascular Diagnosis and Therapy, 2023
Crossref
9 words — < 1%

60 Zong-Jie Fu, Zhi-Yu Wang, Lian Xu, Xiao-Hui Chen et al. "HIF-1α-BNIP3-mediated mitophagy in tubular cells protects against renal ischemia/reperfusion injury", Redox Biology, 2020
Crossref
9 words — < 1%

61 estudogeral.sib.uc.pt
Internet
9 words — < 1%

62 tessera.spandidos-publications.com
Internet
9 words — < 1%

63 www.biolifesas.org
Internet
9 words — < 1%

64 www.nature.com
Internet
9 words — < 1%

65 Anthony R. Anzell, Rita Maizy, Karin Przyklenk, Thomas H. Sanderson. "Mitochondrial Quality Control and Disease: Insights into Ischemia-Reperfusion Injury", Molecular Neurobiology, 2017
Crossref
8 words — < 1%

66 B Das. "Mitochondrial KATP channel activation is important in the antiarrhythmic and cardioprotective effects of non-hypotensive doses of nicorandil and cromakalim during ischemia/reperfusion: a study in an intact anesthetized rabbit model", Pharmacological Research, 2003
Crossref
8 words — < 1%

67 Bednarczyk, P., A. Koziel, W. Jarmuszkiewicz, and A. Szewczyk. "Large-conductance Ca2+-activated potassium channel in mitochondria of endothelial EA.hy926 cells", AJP Heart and Circulatory Physiology, 2013.
Crossref
8 words — < 1%

68 Bin Zhang, Jiachang Yang, Xiayun Li, Hanzhao Zhu et al. "Tetrahydrocurcumin ameliorates postinfarction cardiac dysfunction and remodeling by inhibiting oxidative stress and preserving mitochondrial function via SIRT3 signaling pathway", Phytomedicine, 2023
Crossref
8 words — < 1%

69 Binwu Hu, Shuo Zhang, Weijian Liu, Peng Wang et al. "Inhibiting Heat Shock Protein 90 Protects Nucleus Pulposus-Derived Stem/Progenitor Cells From Compression-Induced Necroptosis and Apoptosis", Frontiers in Cell and Developmental Biology, 2020
Crossref
8 words — < 1%

70	Carles Cantó. "Mitochondrial Dynamics: Shaping Metabolic Adaptation", Elsevier BV, 2018<br>Crossref	8 words — < 1%
71	Dong Li, Mi Liu, Tian-Qi Tao, Dan-Dan Song, Xiu-Hua Liu, Da-Zhuo Shi. " Saponin Attenuates Cardiomyocyte Apoptosis and Opening of the Mitochondrial Permeability Transition Pore in a Rat Model of Ischemia/Reperfusion ", Cellular Physiology and Biochemistry, 2014<br>Crossref	8 words — < 1%
72	Gerd Heusch, Ioanna Andreadou, Robert Bell, Edoardo Bertero et al. "Health position paper and redox perspectives on reactive oxygen species as signals and targets of cardioprotection", Redox Biology, 2023<br>Crossref	8 words — < 1%
73	Hao Wu, Huifang Wei, Sheikh Arslan Sehgal, Lei Liu, Quan Chen. "Mitophagy receptors sense stress signals and couple mitochondrial dynamic machinery for mitochondrial quality control", Free Radical Biology and Medicine, 2016<br>Crossref	8 words — < 1%
74	Jianpeng Zhang, Lin Sun, Weiqiang Li, Yanyu Wang, Xinzhen Li, Yang Liu. "Overexpression of macrophage stimulating 1 enhances the anti-tumor effects of IL-24 in esophageal cancer via inhibiting ERK-Mfn2 signaling-dependent mitophagy", Biomedicine & Pharmacotherapy, 2019<br>Crossref	8 words — < 1%
75	Jin-Fu Peng, Oluwabukunmi Modupe Salami, Cai Lei, Dan Ni, Olive Habimana, Guang-Hui Yi. "Targeted mitochondrial drugs for treatment of Myocardial ischemia-reperfusion injury", Journal of Drug Targeting, 2022<br>Crossref	8 words — < 1%

76	Jingjing Zhang, Jianan Qian, Wei Zhang, Xiangfan Chen. "The pathophysiological role of receptor-interacting protein kinase 3 in cardiovascular disease", Biomedicine & Pharmacotherapy, 2023<br>Crossref	8 words — < 1%
77	Jinhui Wang, Junbo Zou, Yajun Shi, Nan Zeng, Dongyan Guo, He Wang, Chongbo Zhao, Fei Luan, Xiaofei Zhang, Jing Sun. "Traditional Chinese medicine and mitophagy: A novel approach for cardiovascular disease management", Phytomedicine, 2024<br>Crossref	8 words — < 1%
78	Martínez-Reyes, Inmaculada, and José M. Cuezva. "The H+-ATP synthase: A gate to ROS-mediated cell death or cell survival", Biochimica et Biophysica Acta (BBA) - Bioenergetics, 2014.<br>Crossref	8 words — < 1%
79	Mi Xiang, Xin Zhao, Yingdong Lu, Yang Zhang, Fan Ding, Lifei Lv, Yuling Wang, Zihuan Shen, Li Li, Xiangning Cui. "Modified Linggui Zhugan Decoction protects against ventricular remodeling through ameliorating mitochondrial damage in post-myocardial infarction rats", Frontiers in Cardiovascular Medicine, 2023<br>Crossref	8 words — < 1%
80	S. Zhang, H. Liu, H. Yu, G. J.S. Cooper. "Fas-Associated Death Receptor Signaling Evoked by Human Amylin in Islet -Cells", Diabetes, 2007<br>Crossref	8 words — < 1%
81	Zheng Li, Jihong Xing. "Contribution and therapeutic value of mitophagy in cerebral ischemia-reperfusion injury after cardiac arrest", Biomedicine & Pharmacotherapy, 2023<br>Crossref	8 words — < 1%

82	coek.info<br>Internet	8 words — < 1%
83	digitalcommons.library.uab.edu<br>Internet	8 words — < 1%
84	kups.ub.uni-koeln.de<br>Internet	8 words — < 1%
85	portlandpress.com<br>Internet	8 words — < 1%
86	research.vu.nl<br>Internet	8 words — < 1%
87	www.benthamscience.com<br>Internet	8 words — < 1%
88	www.em-consulte.com<br>Internet	8 words — < 1%
89	www.openaccessjournals.com<br>Internet	8 words — < 1%
90	www.science.org<br>Internet	8 words — < 1%
91	zir.nsk.hr<br>Internet	8 words — < 1%
92	Boyu Xia, Qi Li, Koulong Zheng, Jingjing Wu, Chao Huang, Kun Liu, Qingsheng You, Xiaomei Yuan. "Down-regulation of Hrd1 protects against myocardial ischemia-reperfusion injury by regulating PPARα to prevent oxidative stress, endoplasmic reticulum stress, and cellular apoptosis", European Journal of Pharmacology, 2023<br>Crossref	7 words — < 1%

93 Chunling Huang, Hao Yi, Ying Shi, Qinghua Cao, Yin Shi, Delfine Cheng, Filip Braet, Xin-Ming Chen, Carol A. Pollock. "KCa3.1 Mediates Dysregulation of Mitochondrial Quality Control in Diabetic Kidney Disease", Frontiers in Cell and Developmental Biology, 2021 — 7 words — < 1%
Crossref

94 Feifan Jia, Yuanyuan Chen, Gaojie Xin, Lingmei Li et al. "Shuangshen Ningxin capsule alleviates myocardial ischemia–reperfusion injury in miniature pigs by modulating mitophagy: network pharmacology and experiments in vivo", Chinese Medicine, 2023 — 7 words — < 1%
Crossref

95 "Mitochondrial Biology and Experimental Therapeutics", Springer Science and Business Media LLC, 2018 — 6 words — < 1%
Crossref

96 Chunzhu Wei, Jingbo Wang, Jintao Yu, Qing Tang et al. "Therapy of traumatic brain injury by modern agents and traditional Chinese medicine", Chinese Medicine, 2023 — 6 words — < 1%
Crossref

97 Liang, Qiangrong, and Satoru Kobayashi. "Mitochondrial quality control in the diabetic heart", Journal of Molecular and Cellular Cardiology, 2015. — 6 words — < 1%
Crossref

98 Lin Li, Lei Lin, Shaoqing Lei, Si Shi, Chun Chen, Zhongyuan Xia. "Maslinic Acid Inhibits Myocardial Ischemia–Reperfusion Injury-Induced Apoptosis and Necroptosis via Promoting Autophagic Flux", DNA and Cell Biology, 2022 — 6 words — < 1%
Crossref

99 Lin Li, Yunfeng Zhou, Yanlin Li, Lili Wang, Lan Sun, Lidong Zhou, Hiderori Arai, Yun Qi, Yang Xu. "Aqueous extract of Cortex Dictamni protects H9c2 cardiomyocytes from hypoxia/reoxygenation-induced oxidative stress and apoptosis by PI3K/Akt signaling pathway", Biomedicine & Pharmacotherapy, 2017 — 6 words — < 1%
Crossref

100 Mingjie Zhou, Huanhuan Ren, Jichun Han, Wenjuan Wang, Qiusheng Zheng, Dong Wang. " Protective Effects of Kaempferol against Myocardial Ischemia/Reperfusion Injury in Isolated Rat Heart via Antioxidant Activity and Inhibition of Glycogen Synthase Kinase-3 ", Oxidative Medicine and Cellular Longevity, 2015 — 6 words — < 1%
Crossref

101 Qin Wang, Ping Lin, Peng Li, Li Feng, Qian Ren, Xiaofeng Xie, Jing Xu. "Ghrelin protects the heart against ischemia/reperfusion injury via inhibition of TLR4/NLRP3 inflammasome pathway", Life Sciences, 2017 — 6 words — < 1%
Crossref

102 Rui Chen, Mengwen Niu, Xin Hu, Yuquan He. "Targeting mitochondrial dynamics proteins for the treatment of doxorubicin-induced cardiotoxicity", Frontiers in Molecular Biosciences, 2023 — 6 words — < 1%
Crossref

103 Ruxiu Liu, Xing Chang, Jie Li, Yao Shunyu. "Zishen Huoxue Recipe Protecting Mitochondrial Function of Hypoxic/Reoxygenated Myocardial Cells through mTORC1 Signaling Pathway", Evidence-Based Complementary and Alternative Medicine, 2020 — 6 words — < 1%
Crossref

104 Shiwani Kumari, Ambica Singh, Abhinay Kumar Singh, Yudhishthir Yadav et al. "Circulatory GSK-3β: — 6 words — < 1%

Blood-Based Biomarker and Therapeutic Target for Alzheimer’s Disease", Journal of Alzheimer's Disease, 2022
Crossref

105 Wulf Dröge. "Free Radicals in the Physiological Control of Cell Function", Physiological Reviews, 2002
Crossref
6 words — < 1%

106 Yao Li, Hao Hao, Haozhen Yu, Lu Yu, Heng Ma, Haitao Zhang. "Ginsenoside Rg2 Ameliorates Myocardial Ischemia/Reperfusion Injury by Regulating TAK1 to Inhibit Necroptosis", Frontiers in Cardiovascular Medicine, 2022
Crossref
6 words — < 1%

107 Ying Tan, David Mui, Sam Toan, Pingjun Zhu, Ruibing Li, Hao Zhou. "SERCA Overexpression Improves Mitochondrial Quality Control and Attenuates Cardiac Microvascular Ischemia-Reperfusion Injury", Molecular Therapy - Nucleic Acids, 2020
Crossref
6 words — < 1%

EXCLUDE QUOTES	OFF	EXCLUDE SOURCES	OFF
EXCLUDE BIBLIOGRAPHY	ON	EXCLUDE MATCHES	OFF
